# Novel C15 Triene Triazole, D-A Derivatives Anti-HepG2, and as HDAC2 Inhibitors: A Synergy Study

**DOI:** 10.3390/ijms19103184

**Published:** 2018-10-16

**Authors:** Zhiwen Qi, Chengzhang Wang, Jianxin Jiang, Caie Wu

**Affiliations:** 1 Institute of Chemical Industry of Forest Products, China Academy of Forestry, Nanjing 210042, China; angelkissgod@163.com; 2 Key and Open Laboratory on Forest Chemical Engineering, State Forestry Administration, Nanjing 210042, China; 3 College of Material Science and Technology, Beijing Forestry University, Beijing 100083, China; jiangjx@bjfu.edu.cn; 4 College of Light Industry and Food Engineering, Nanjing Forestry University, Nanjing 210000, China; wucaie@njfu.edu.cn

**Keywords:** urushiol, triazole, diels-alder, pechmann, anti-tumor, HDAC2

## Abstract

A series of novel C15 urushiol derivatives were designed by introducing a pechmann structure and F-, Cl-, and Br-nitro substituents with different electronic properties into its alkyl side chain, as well as a triazolyl functional group in its aromatic oxide. Their chemical structures were determined based on the analysis of the NMR (nuclear magnetic resonance) spectroscopic and mass spectrometric data. The results showed that compound **4** exhibited a strong inhibition of the HepG2 cell proliferation (half maximal inhibitory concentration (IC50): 2.833 μM to human hepatocellular carcinoma (HepG2), and 80.905 μM to human normal hepatocytes (LO2)). Furthermore, it had an excellent synergistic effect with levopimaric acid. The nitrogen atom of the triazole ring formed a hydrogen-bonding interaction with Gly103, Gly154, and Tyr308, which made compound **4** bind to histone deacetylase (HDAC)2 more tightly. One triazole ring and His33 formed a π–π stacking effect; the other, whose branches were deep into the pocket, further enhanced the interaction with HDAC2. Meanwhile, compound **4** involved a hydrophobic interaction with the residues Phe210 and Leu276. The hydrophobic interaction and π–π stacking provided powerful van der Waals forces for the compounds.

## 1. Introduction

Recently, an increasing number of anti-tumor drugs are being derived from plants. Chemotherapeutic agents such as paclitaxel [1], cisplatin [2], and adriamycin [3] are relevant to tumor apoptosis. Urushiol [4,5], the main constituent of lacquer, stimulates the activity of certain cells in mice and reduces their risk of fatty liver disease [6]. It modulates the activity of SIRT (sirtuin) inhibitors [7], and is effective in inhibiting cancer progression, tumor angiogenesis, and nuclear transcription factor-κB. Moreover, urushiol works well in scavenging, inhibiting free radicals, resisting streptococcus mutans [8], and suppressing food corruption and microbial growth [9]. However, urushiol’s sensitization combined with keratin or proteins in the cell membrane forms intact antigens, which attack the 4th-, 5th-, and 6th-position C atoms of benzene rings [10,11,12,13]. Novel urushiol derivatives have an anti-platelet agglutination activity. When tested in mice, they markedly reduce the levels of TNF-α and IL-1β [14], as well as the anti-tumor virus in liver, lung, and breast cancer, by preventing tumor cell proliferation and inducing tumor cell differentiation [15,16,17,18], in addition to the degree of alcohol-induced steatosis; they also contribute to improving the immune capacity and potentially treat alcoholic liver disease (ALD) [19]. In addition, combined with triple therapy, urushiol increases the eradication rate of *Helicobacter pylori* to 100% [6,20], while synergistic drugs (2% chlorhexidine (CHX), 6% NaOCl, and 0.01% urushiol solution) decrease the growth rate of *Streptococcus mutans* [8]. As another main constituent of turpentine, levopimaric acid and its derivatives display activity on renal cancer, leukemia, colon cancer, and breast cancer cell lines at a concentration of 10^−5^ M [21]. Some levopimaric derivatives act against respiratory viruses, the papilloma virus, and the hepatitis B and C viruses [22] (Figure S1).

Notably, our research has indicated that the urushiol derivatives have a remarkable binding affinity with good scoring of histone deacetylase (HDAC)2 and HDAC8 [23,24]. Histone deacetylases (HDACs) are a group of zinc metalloenzymes that regulate chromatin remodeling and gene transcription by catalyzing the removal of an acetyl group moiety from the Ɛ-amino groups of the lysine residues on the amino terminal tails of the core histones [25]. They are critical for controlling gene expression, aiding cell growth, and for proliferation [26]. The overexpression of HDACs has been linked to the development of different cancers in humans [27]. Thus, HDAC has been applied as a valuable target enzyme for anticancer therapies.

As a chemical bond linker, triazole compounds can inhibit tumor cell proliferation, and can induce leukemia and breast cancer cell apoptosis [28,29]. Triazole acts as a variety of anionic, neutral molecules, and even as a bio-macromolecular receptor compound, because the triazole electron-rich complex easily and strongly combines with metal ions and cations. Triazole rings form supramolecules with anions through hydrogen bonds. Due to the lack of electronic properties, it reacts with the anion complexation after quaternization [28,29]. Moreover, the pechmann structure [30,31,32] and maleic anhydride unit structure [33,34] show a high biological activity, especially in sterilization.

On account of the continuation of the discovery of new antitumor compounds from natural products, it is of great interest to us to synthesize and synergize product-based antitumor agents. Considering urushiol’s anticancer activities and its structural similarities to Suberoylanilide hydroxamic acid (SAHA), an HDAC inhibitor, we sought to find some novel urushiol derivatives as potential HDAC2-selective inhibitors, in order to detect more valuable candidates for antitumor therapy [23,24]. Herein, we designed and synthesized a series of novel derivatives from readily available C15 triene urushiol, and tested their antitumor activities against human hepatocellular carcinoma (HepG2). The best bioactive compound to suppress HepG2 was first evaluated using flow cytometry (FCM) and a molecule docking analysis. Then, we studied their enzymatic bioactivity against HDAC2 (Western blot) and analyzed the probable binding-modes of the most active compound using molecular docking algorithms.

## 2. Results

### 2.1. Synthesis of 3-((8Z,11E,13Z)-Pentadeca-8,11,13-Trien-1-yl) Benzene-1,2-Diol Derivatives

The C15 triene urushiol with a catechol structure, a long C15 alkyl, and a conjugated diene group, is one of the most important bioactive natural resource compounds [7,8,9]. As a result, it is meaningful to exploit urushiol’s potential application in natural medicine. To bring out an efficient synthesis of the urushiol derivatives, we triggered these reactions in various simple or mild conditions. For example, we did a D–A reaction under 160 °C (compounds **15**–**20** reaction temperature) for 6 h, and added a pechmann structure (compounds **8**–**12**) with 4-methane benzene mono sulfonic acid and modifying hydroxyl with K_2_CO_3_. Interestingly, when the reaction time was prolonged, the D–A products were formed in hydrolysis. However, we did not obtain any products when using strong acid or alkali like H_2_SO_4_/HCl or NaOH. This was the result of urushiol’s easy polymerization characteristic. After investigation, we found that compounds **3** and **6** formed aromatic oxide at position 2 more easily than phenolic hydroxyl group at position 1, whose hydrogen atom was harder to remove [35]. Therefore, we increased the molar concentration of K_2_CO_3_ to 0.5 M, which would help gain a higher yield of product (75–99%).

### 2.2. Anti-Tumor Bioactivity

**Figure 1:**
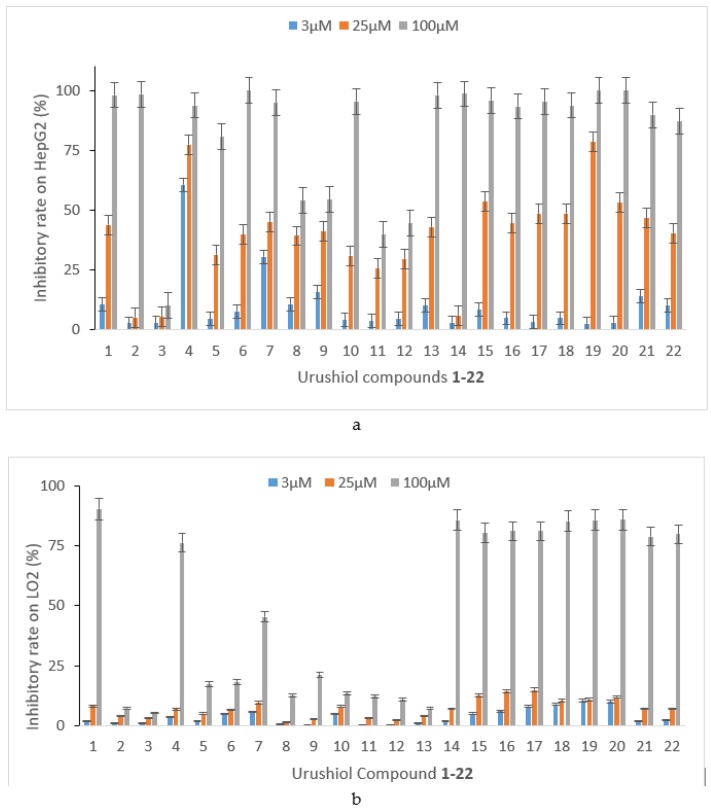
Urushiol compounds’ (**1**–**22**) inhibitory effect on (**a**) human liver hepatocellular carcinoma (HepG2) and (**b**) liver normal hepatocellular (LO2) cell proliferation. Three groups were very different from each other (*p* < 0.05) (details show in Supplementary Materials). (**a**) The inhibitory effect of compounds **1**–**22** on the HepG2 cell proliferation; and (**b**) the inhibitory (toxicity) effect of compounds **1**–**22** on the LO2 cell proliferation.

**Figure 2:**
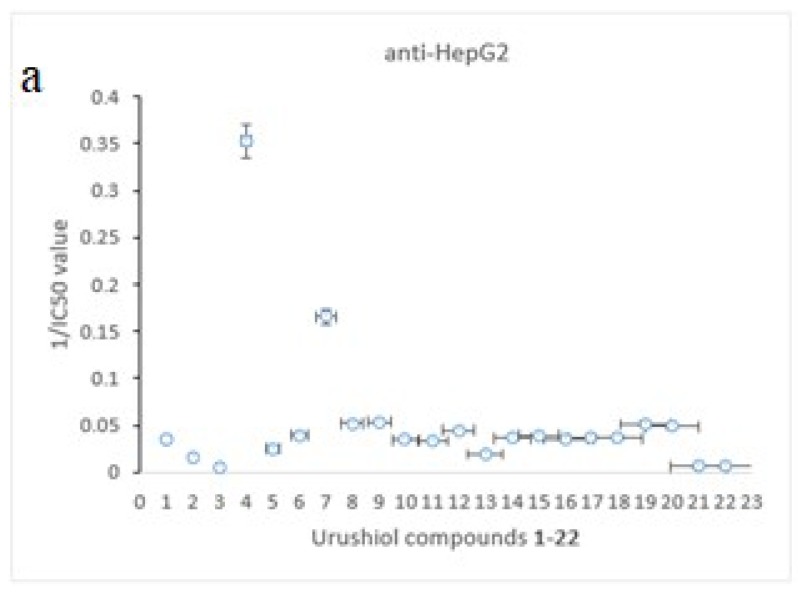

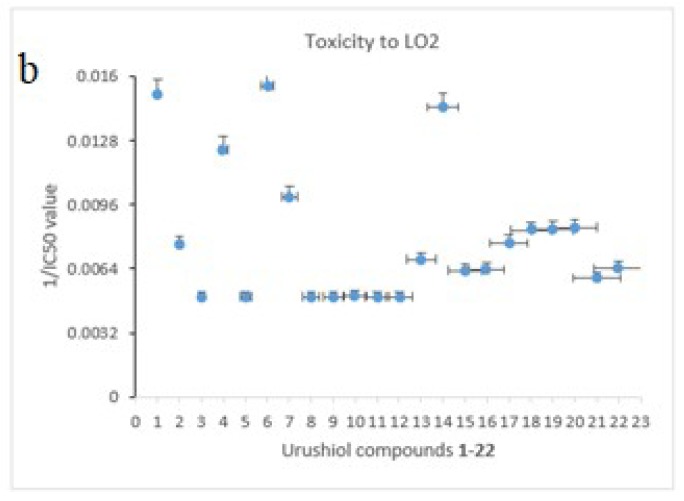
The relationship between compounds’ **1**–**22** anti-HepG2 or toxicity to LO2 and 1/IC50 value (*p* < 0.05) (details are in the Mupplementary Materials). (**a**) Relationship between compounds **1**–**22** and the anti-HepG2 1/IC50 value, and (**b**) relationship between compounds **1**–**22** and toxicity to LO2 1/IC50 value.

Preliminary in vitro screening results of the title compounds for antitumor activity against HepG2 (human hepatocellular carcinoma) and human normal hepatocytes (LO2) were determined by MTT assay (thiazolyl blue salt colorimetry) (see Figure 1 and Figure 2). The results indicate that half of the synthetic compounds had up to almost 50% inhibition rates against the HepG2 growth at concentrations of 25 μM.

As shown in Figure 1 and Figure 2, all of the compounds (**1**–**22**) were evaluated for the inhibitory effects of the proliferation on HepG2 and LO2 cells using MTT assay. The quality structure–activity relationship (QSAR) of the above-mentioned urushiol derivatives proved that urushiol with the D–A structure in its alkyl chain, the pechmann structure; phenylboronic group [36]; and F-, Cl-, and Br-nitro substituents played an effective role in attenuating and increasing the efficiency; the urushiol triazole derivative also presented a same effect. However, unlike the ether bond, introducing alkyne was not beneficial for urushiol’s bioactivity. This finding provided the experimental basis for continually attenuating and improving the urushiol derivatives in the subsequent syntheses.

The bioassays of the urushiol derivatives indicated that the C-2, C-7/C-8, and C-19/C-22 bonds are important for activating antitumor properties [36]. To further evaluate the inhibitory potencies of some of the synthesized compounds, we determined the half maximal inhibitory concentration (IC50) values of the compounds with high inhibition rate (>75%). As summarized in Table 1, some tested compounds presented different antitumor activities. Specifically, triazole compound **4**, which bears a triazole plentiful electronic structure, had high antitumor activity against HepG2 with IC50 reaching 2.833 μM. Triazole derivatives **4** and **7** displayed a better antitumor function and protected normal cell as well. Noted that, compound **4** and **7** significantly decreased the inhibition activity of the human normal liver cells (LO2) than other tested compounds; the inhibitory effect of these two on LO2 was dramatically lower than that of compound **6** and **10**. In addition, inhibition of LO2 cells of the compounds, especially compound **6** (15% or lower), was a lot weaker than that of urushiol. Anti-HepG2 IC50 value of compounds **1**, **2**, **4**, **7**, **10**, **22** and other compounds were almost less than 50 μM, and they performed well on restraining human liver cancer cells (HepG2). The findings above demonstrated that the anti-cancer activity of the target compounds referred to their dose.

Introducing the triazole and pechmann modification of urushiol enhanced the antitumor activity, whereas increasing the chain length of the linear alkyne substituents (**3**, **6**, and **22**) led to a decreased activity. Moreover, the triazole-containing compounds showed the highest antitumor effect on HepG2 and the low hepatic lesion, being superior to paclitaxel, the positive control (LO2, 24 h, IC50 = 28.5 μg/mL). [37] Among them, compound **10**, the lowest inhibitor of LO2 (IC50 = 198 μM), was superior to compounds **4** and **7** in its toxicity on LO2. In addition, compounds **15**–**20** (anti-HepG2 IC50 was 20–30 μM) had a stronger anti-tumor bioactivity when they were added with groups like maleic anhydride.

### 2.3. QSAR (Quantitative Structure Activity Relationship)

Paclitaxel can specifically bind to the β-protein position for promoting its own stability, so that cancer cells stagnate in the mitosis period, which prevents the normal division of cancer cells [38,39,40,41]. In our study, compound **4**’s inhibitory activity against the HepG2 cells was 2.833 μM, and its IC50 value was closest to paclitaxel’s. In particular, the ratio of compound **4**’s value on LO2 was 20 times higher than its value on HepG2, and the ratio of compound **7** was 15 times higher than its value on HepG2. As compounds **4** and **7**’s C5 position had a long chain of alkanes, we hypothesized that the reason the pyrimidine compounds restrained the human hepatocellular carcinoma cells’ proliferation is be related to the triazole nitrogen heterocyclic chains and the lipid-soluble alkane side chain.

In addition, compound **1** had a greater toxicity on the liver cancer HepG2 cells and on the normal liver cells LO2 than compound **2**. Compound **3** was less effective at inhibiting HepG2, probably due to the fact that the alkyne was in a state of electronic shortage, which resulted in an electron homogeneity and in reducing its inhibitory activity [12]. Furthermore, although compound **10** had a strong inhibitory effect on HepG2, it was toxic to LO2 in normal hepatocytes.

As illustrated in Figures S2 and S3, as the drug concentration of compound **4** increased, the growth of the HepG2 cells was gradually suppressed. The negative control group showed uniform cytoplasm, clear nucleoli, full cells, and a good spindle shape. When the concentration of compound **4** increased, the HepG2 cells’ morphology obviously changed, and the number of cells went down significantly. The cells became round, the volume became smaller, and the refraction decreased. In a high concentration of compound **4**, the cells were suspended and were shedding. It was noted that the cells did not adhere to the wall anymore; they slowly became round in shape with the membrane rupturing, shrinking, and finally losing vitality.

### 2.4. Molecular Docking

Our research results have suggested that the binding pattern of urushiol derivative ligands such as ligand compound **4** to the HDAC2 receptor [23,24] could be detected by the molecular docking technique. We attained the HDAC2 X-ray crystal structure (PDB no. 4LXZ) from the RCSB Protein Data Bank (http://www.rcsb.org) with a resolution of 1.85 Å, and used the UCSF Chimera software [42] to build the three-dimensional structure of the ligand and perform the energy optimization.

Firstly, we employed the Dock Prep module and the AMBER ff14SB force field to introduce hydrogen atoms, and added an AM1-BCC charge [43,44]. Secondly, we applied the DMS tool in Chimera to form the molecular surface of the receptor with a 1.4 Å radius probe. The X-ray crystal structure demonstrated a suitable binding site, where the sphgen module was used to generate the spheres surrounding the active position, and a grid file was used to generate a grid file for the grid-based energy rating evaluation. Thirdly, we performed semi-flexible docking using the DOCK 6.8 program [45,46], producing 10,000 different conformational orientations and obtaining electrostatic and van der Waals interactions between the ligand molecules and the binding sites. Then, we calculated the grid score, and achieved the best scoring conformation from the cluster analysis (RMSD threshold of 2.0 Å). Finally, we utilized PyMOL (PyMOL molecular graphics system, version 1.8, 2015.) to create the images.

#### 2.4.1. Conjunction Conformation Score

As seen in the calculation results, we found that the binding sites had multiple docking conformations after using the DOCK 6.8 program to forecast the mode of compound **4** binding in HDAC2 (Table 2 represents the scoring conditions). According to the scoring and combining mode (Table 3), we chose the second docking conformation to analyze the binding mode.

#### 2.4.2. Binding Pattern Analysis

**Figure 3:**
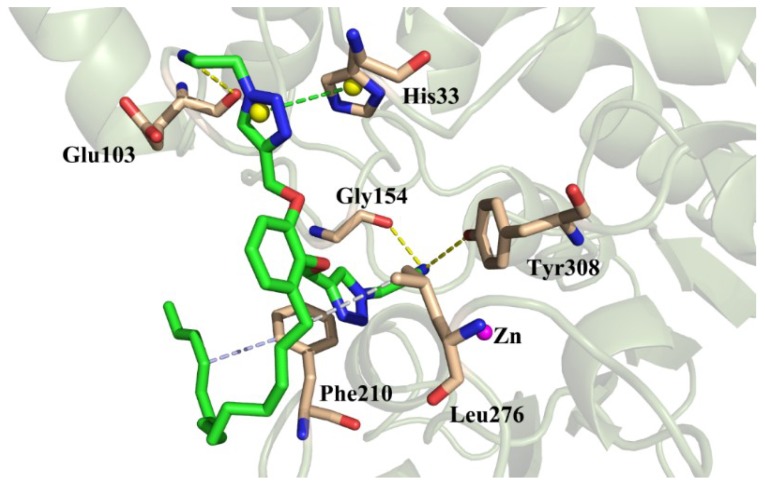
Compound **4**’s molecular docking modeling in the active site of histone deacetylase (HDAC)2. As seen in the picture, the green stick represents the ligands, the wheat stick stands for the key residues, the cartoon denotes the protein smudge color, and the yellow dotted line indicates a hydrogen bond.

As shown in Figure 3, the nitrogen atom of the aminoethyl group on the triazole ring formed a hydrogen-bonding interaction with Gly103, Gly154, and Tyr308, respectively, to make the binding tighter. One triazole ring formed a π–π stacking effect with His33, the other, whose branches went deep into the pocket, further enhanced the interaction with HDAC2. At the same time, compound **4** formed a hydrophobic interaction with the residues Phe210 and Leu276. The hydrophobic interaction and π–π stacking provided a strong van der Waals forces for the compounds (Grid_vdw = −56.1389 kcal/mol).

Based on the theory of molecule dock, the C15 urushiol triazole was introduced as the potential HDAC2 inhibitor. The binding affinity toward HDAC2 of all of the designed compounds was screened by Glide docking. The results showed that six compounds had excellent grid scores (−41.8 to −70.5). The grid docking studies revealed that introducing triazole could increase the binding affinity significantly. The molecular docking studies signified that the Zn^2+^ coordination, hydrogen bonding, and hydrophobic interaction contributed to the high binding affinity of these compounds toward HDAC2. In addition, Gly103, Gly154, Tyr308, His33, Phe210, and Leu276 contributed favorably to the binding between the enzyme and the compounds. On the basis of the binding free energy calculations, all of the complexes had a good stability with the moderate hydrogen bonding, and the Zn^2+^ coordination had low values of binding free energies. Moreover, as we know from the results of the binding energy decomposition, the interactions between the van der Waals and electrostatics guaranteed these complexes’ stability availably. This study has implied that the urushiol derivatives have the potential to be potent HDAC2 binding agents. Further studies on the synthesis and determination of their HDAC2 inhibitory properties will be done in the future.

### 2.5. Western Blot Results

We have found that the urushiol derivatives have a better inhibitory effect on the HepG2 cells than the urushiol mother compounds, after screening their anti-HepG2 activity. This inspired us to select compound **4** as the research object. After all of the protein was extracted from the logarithmic growth phase of the HepG2 cell line, we detected compound **4**’s expression of HDAC2 using the Western blot method.

**Figure 4:**
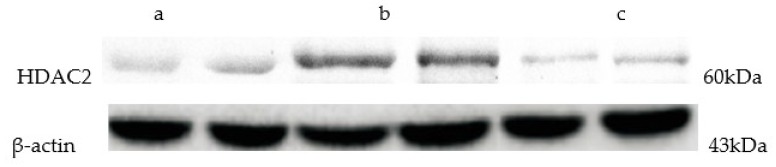
The protein expression of HDAC2 in different groups ((**a**) LO2 cells; (**b**) HepG2 cells; (**c**) compound **4** on HepG2 cells).

As showed in Figure 4, there were clear bands with the expected molecular weight. Compared with LO2, the expression of the HDAC2 protein in the compound **4** group was significantly lower than that in the HepG2 group (*p* < 0.05). It indicated that compound **4** could down-regulate the expression of the HepG2 cells and alleviate the pathological changes of HCC. The mechanism was presumably associated with the inhibition of the HDAC2 expression. In short, compound **4** had a good inhibitory effect on the expression of enzyme HDAC2, and the calculated results were better than that of SAHA [23,24]. This study has explained why compound **4** inhibits HDAC2, but the specific inhibitory effect of urushiol on enzyme, such as HDACs, needs more extensive research.

### 2.6. Flow Cytometry Analysis

A flow cytometry analysis is a biological particle of rapid quantitative measurement and biological analysis method of the high speed linear flow of cells. On the basis of the preliminary screening results, the HepG2 cells were incubated with various concentrations of compound **4** so as to investigate the effect of the propidium iodide (PI) single staining assay for cell cycle, Annexin-V FITC/PI double staining for apoptosis, JC-1 staining assay mitochondrial membrane potential, and the calcium content detection (details of these methods are in the Supplementary Materials).

#### 2.6.1. PI Single Staining Assay for Cell Cycle

**Figure 5:**
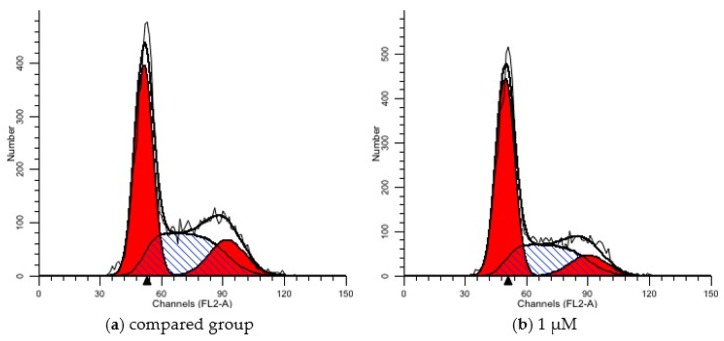

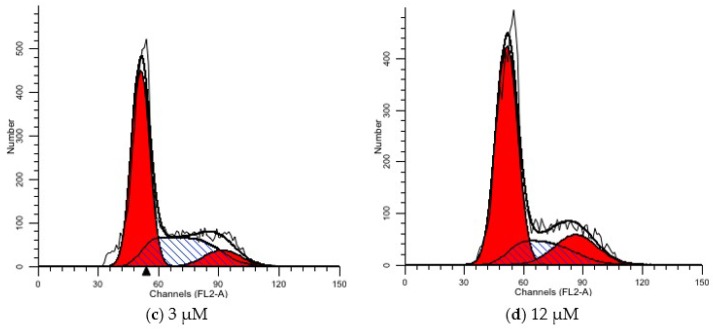
Different concentration of compound **4** of (**a**) compared group, (**b**) 1 μM, (**c**) 3 μM and (**d**) 12 μM effect on the HepG2 cell cycle of humans. *x*-axis: Channels (FL2-A); *y*-axis: Number. Red zone from left to right: Dip G1 and G2; middle blue cross-court zone: Dip S.

Pyridine iodide (Propidium) is a fluorescent dye for double stranded DNA. After the intracellular DNA was stained with pyridine iodide, the content of the DNA was measured by flow cytometry, and then the cell cycle and apoptosis were analyzed according to the distribution of the DNA content of compound **4** on the cell cycle of human hepatocellular carcinoma HepG2. After the HepG2 cells were treated with 1 μM, 3 μM, and 12 μM of compound **4** for 72 h, we measured the number of cells and detected them using flow cytometry (see Table 4 and Figure 5). With the increase of the drug concentration, the HepG2 cells’ distribution changed significantly in the cell cycle. Compared with the blank control group, the proportion of cells in the G0/G1 phase of the compound **4** group increased gradually, but decreased in the S and G2/M phase. The compound **4** dose groups varied from each other (*p* < 0.05). It was seen that compound **4** could block the cells in the G0/G1 phase and S phase.

#### 2.6.2. Annexin-V FITC/PI Double Staining for Apoptosis

**Figure 6:**
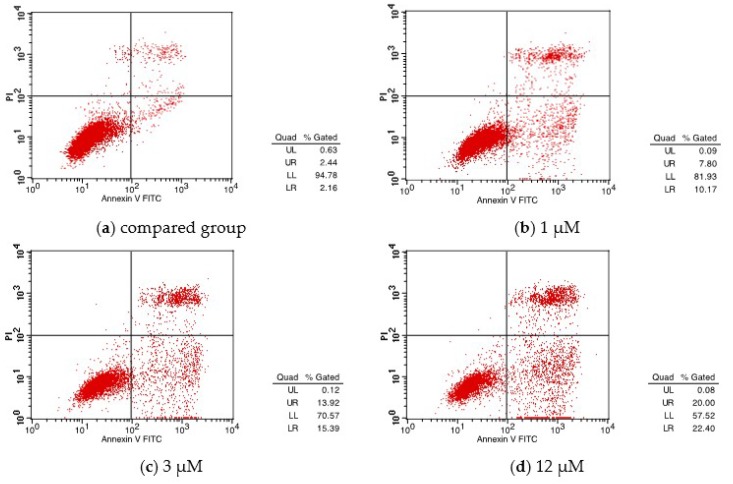
Different concentration of compound **4** of (**a**) compared group, (**b**) 1 μM, (**c**) 3 μM and (**d**) 12 μM on the HepG2 cell apoptosis rate. *x*-axis: Annexin V FITC; *y*-axis: PI.

Annexin V is a Ca^2+^ dependent phospholipid binding protein that has a high affinity for phosphatidylserine. Therefore, Annexin V is regarded as one of the sensitive indexes for the detection of early cell apoptosis. Annexin V is labeled with fluorescein (EGFP, FITC) and propidium iodide (PI), which is used as a fluorescence probe to detect apoptosis using a fluorescence microscope or flow cytometry. After 1 μM, 3 μM, and 12 μM of compound **4** were treated on the HepG2 cells for 72 h, the flow cytometry was used to detect the change of the apoptosis rate. The apoptosis rate of the 12 μM group was obviously higher than that of the control group in a dose-dependent manner. The apoptosis rates of the three concentrations were 17.97%, 29.31%, and 42.40%, respectively (see Table 5 and Figure 6).

#### 2.6.3. JC-1 Staining Assay Mitochondrial Membrane Potential

**Figure 7:**
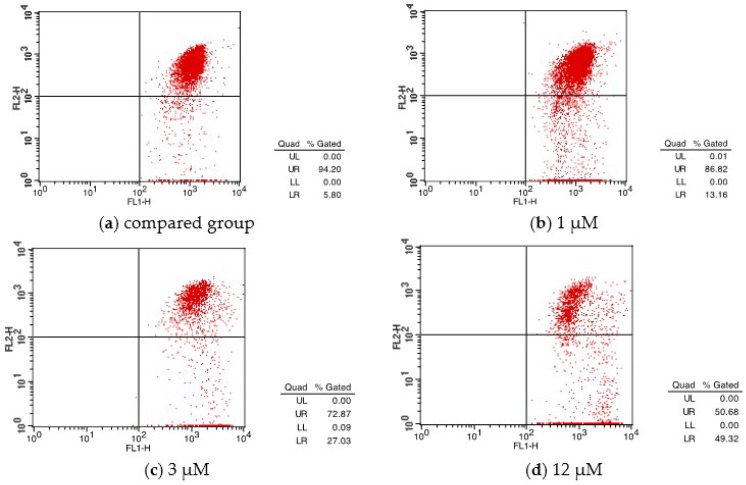
Different concentration of compound **4** of (**a**) compared group, (**b**) 1 μM, (**c**) 3 μM and (**d**) 12 μM on HepG2 effect of the mitochondrial membrane potential. *x*-axis: FL1-H; *y*-axis: FL2-H.

As a fluorescent probe, JC-1 can quickly and sensitively detect the changes of the mitochondrial membrane potential in cells, tissues, or purified mitochondria. It is a landmark event in the early stage of cell apoptosis, where the mitochondrial membrane potential is destroyed. The release of cytochrome C is accompanied by the complete loss of the membrane potential, which leads to the cascade effect of the apoptotic enzymes. From Table 6 and Figure 7, the differences between the cells in the control group (UR: 94.20%; LR: 5.8%) and 12 μM experimental group (UR: 50.68%; LR: 49.32%) reflected that the membrane potential and cell apoptosis are decreased by the compound **4** stimulation.

#### 2.6.4. Calcium Ion Content Detection

**Figure 8:**
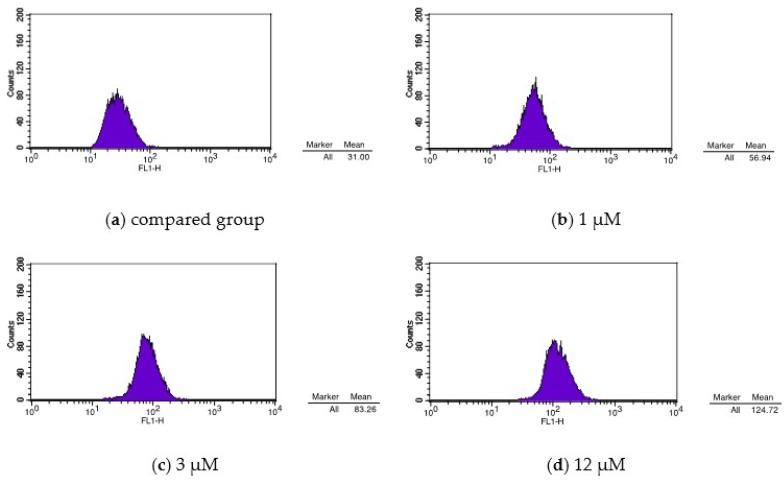
Different concentration of compound **4** of (**a**) compared group, (**b**) 1 μM, (**c**) 3 μM and (**d**) 12 μM effect on the cellular calcium of HepG2. *x*-axis: FL1-H; *y*-axis: Counts.

The correlation between the cell apoptosis and the increase of the Ca^2+^ concentration in the cytoplasm has been confirmed in the experiment [28]. Table 7 and Figure 8 show the Fluo-3 staining test results of the determination of the calcium concentration. In contrast with the blank control group, the cells’ calcium ion concentration presented a right-shifted peak after they were treated with compound **4**, by inducing apoptosis. It signified that the intracellular calcium concentration became higher, which may have caused the activation of the calcium-dependent endonuclease, and induced apoptosis [28].

### 2.7. Synergy

**Figure 9:**
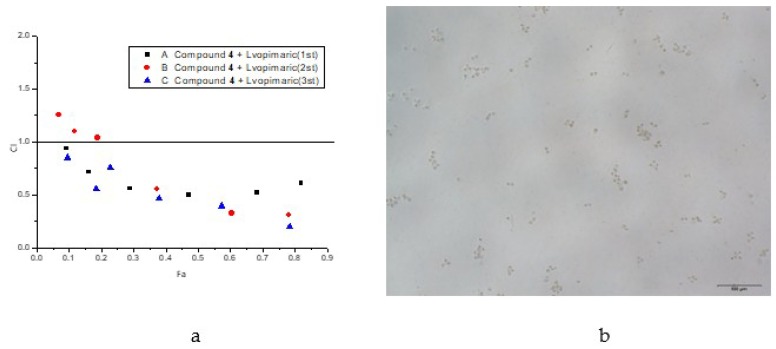
Confidence interval CI–Fa relationship (**a**) (CI = 0.211) and HepG2 apoptosis (**b**). Statistical analysis: A *p* < 0.05 was considered significant.

Levopimaric acid was introduced as the new natural bioactivity molecule with a higher inhibitory effect on HepG2 cell and a good protective effect on the human normal liver cells (LO2). After combining compound **4** with the levopimaric acid, we screened for its inhibitory effects on liver cancer cells (HepG2) (Figure 9). With the increasing drug concentration (*p* < 0.05), the inhibition effect of the cell proliferation of the HepG2 cells dramatically increased after they were treated with compounds **4** and levopimaric acid at different concentrations, for 72 h. It indicated that the inhibitory effects of compound **4** and levopimaric acid on the proliferation of the HepG2 cells were dose-dependent (Table S1). Based on the results above, we did a synergistic experiment [20].

As shown in Figure 9, compound **4** had a synergistic effect with levopimaric acid. When the ratio of compound **4** to levopimaric acid was 7.32 μM to 7.9 μM, the synergistic effect was enhanced, and the confidence interval (CI) value reached 0.308. The CI value decreased as the Fa increased, which suggested that the synergism was more pronounced. When the ratio was 13.88 μM to 7.9 μM, their synergistic effect became remarkable, and CI value was 0.211.

## 3. Experimental

### 3.1. Chemistry

**Scheme 1:**
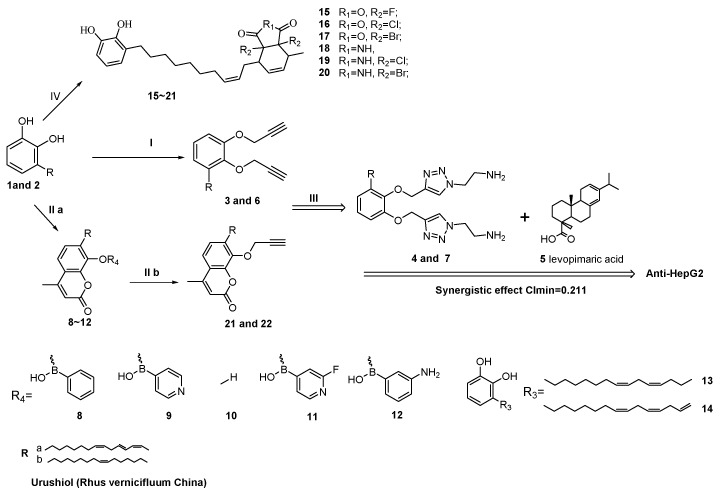
Novel C15 triene urushiol derivatives **1**–**22** (the synthesis details are shown in Scheme S1 and MS, ^1^H/^13^C NMR spectrogram are shown in the supporting information).

The synthetic route of target compounds **1**–**22** and the synergism are outlined in Scheme 1. We used C15 triene/diene urushiol and levopimaric acid, which were separated and purified by us, as the starting materials for the product syntheses.

As for the compound **4** and **7** reactants, compounds **3**, **6**, and **22** were formed by urushiol reacting with bromo propyne under BrØnsted alkaline conditions (K_2_CO_3_) at 60 °C, for 24 h [47,48]. The urushiol pechmann derivative, compound **8**, was formed under an anhydrous solvent acetate/MeCN/EA, SiO_2_/NaHSO_4_ for 4 h at room temperature, and the yield reached more than 90% [30,31,32,49]. For the intermediates, under the azide propylamine and H_2_O/tBuOH-CuSO_4_, compounds **3** and **6** reacted in a ring-closing reaction so as to form triazole products **4** and **7** [50]. The mixture was easily separated by normal column chromatography with petroleum (PE): ethyl acetate (EA) at a ratio of 1:1. The structures of the synthetic compounds were confirmed by ^1^H and ^13^C NMR (Bruker Am-400; CDCl_3_ or DMSO-*d_6_* as the solvent; TMS as the internal standard substance) and GC-MS (Agilent 5977B, Santa Clara, CA, USA) spectroscopic data.

### 3.2. Materials

All of the chemicals were purchased from Aladdin Reagent Co., Ltd. (Shanghai, China). The reagent-grade solvents were purchased from Sino-pharm Chemical Reagent Co., Ltd., Shanghai, China. The anhydrous solvents were dried using anhydrous Na_2_SO_4_ or Mg_2_SO_4_ (the triene/mono-olefins urushiol and levopimaric acid were prepared by our own team). The HepG2 and LO2 cells were treated with different concentrations of compound **4** for 72 h. The morphological changes of the cells were observed and we took pictures later under an inverted microscope (details are in the Supplementary Materials).

### 3.3. Extraction and Synthesis

The isolation and purification of compound **1** (triene urushiol) and compound **2** (mono-olefine urushiol) were as follows: under a vacuum and in nitrogen, fresh, raw lacquer (the triene urushiol mass content was about 50%) was dissolved with methanol; then, it was separated and purified using the chromatographic column. After that, the ratio between the lacquer and methanol (mass ratio) reached 50:200~400 (g:mL); the mass ratio of the silica gel to the concentrated urushiol was 3~4:1, and the column was packed using the wet method. The eluent (EA and PE; total volume of 500 mL) flow was controlled within 10~15 mL/min. With the eluent polarity changing from high to low, the column silica gel color ribbon changed slowly. The colors of the chromatography column were successively black, yellow, and red. Under pressure, the concentrated light red ribbon area of the eluent was concentrated in order to obtain a high purity wine red viscous triene and diene urushiol viscous liquid. They would be further purified by preparative HPLC after we used MeOH:H_2_O (0.25:0.05, *v/v*) as the eluent to yield triene urushiol (brown liquid, 900 mg) and mono-olefins urushiol (brown liquid, 200 mg). The synthetic process is shown in the Supplementary Materials.

## 4. Conclusions

For the first time, urushiol, triazole, and other drug groups were combined together by chemistry bonds. All of the derivatives were first reported as an anti-HepG2 product. It should be noted that the synthetic approach for these category compounds used in the current study was a simple, efficient, low-cost, and high-yielding method. Compared with the parent compounds, not all of the compounds presented a strong anticancer activity, although they had less cytotoxic potential compared with normal LO2 cells.

The effects of C15 urushiol and its triazole derivatives on the apoptosis of tumor cells were verified qualitatively and quantitatively. And, as a result, we found that compounds’ **4** and **7** triazole modifications of urushiol alkyne were able to enhance the anti-HepG2 activity, but the alkyne introduction in the urushiol phenolic hydroxyl group reduced the activity. In addition, some compounds were reported, for the first time, as potential inhibitors of HDAC2. For example, compounds **4** and **7** were found to be the strongest HDAC2 inhibitors in our study. The results indicated that compound **4** is a hopeful adjuvant drug for hepatocellular carcinoma, and it could become a prospective new promising lead as an HDAC2-targeted anticancer drug; compound **10** has great prospects for being a drug. Meanwhile, combining levopimaric acid with compound **4** creates a synergism function on anti-HepG2, which provides experimental evidence for the clinical treatment of hepatocellular carcinoma.

## Figures and Tables

**Table 1:** Some of urushiol derivatives’ inhibitory activity against HepG2 and LO2 in vitro (half maximal inhibitory concentration (IC50), μM, 72 h).

Cpd.	1	2	3	4	5	6	7	10	15	16	22
HepG2 ^a^	28.6	63.2	>200	2.8	39.5	25.0	6.0	28.9	29.4	23.9	65.8
LO2 ^b^	66.1	130.6	>200	80.9	>200	64.0	100.1	198.0	>200	>200	150.5

^a^ HepG2: human liver hepatocellular carcinoma cells; ^b^ no inhibitory activity at 200 µM; Cpd. = compound. Inactive: IC50 > 100 μM; weak activity: IC50 > 30 μM; moderate activity: IC50 < 30 μM; potent activity: IC50 < 10 μM.

**Table 2:** Docking of ligand with receptor histone deacetylase (HDAC)2 (unit: kcal/mol).

Compound	Pose	Grid Score	Grid_vdw	Grid_es	Int_energy
1	1	−46.492264	−40.641602	−5.850661	4.597153
2	1	−44.553402	−40.142418	−4.410985	9.792090
	2	−41.821983	−36.967918	−4.854066	7.414151
3	1	−47.741497	−46.867558	−0.873939	11.230281
4	1	−70.507187	−55.308952	−15.198232	19.825512
	2	−68.278595	−56.138966	−12.139633	18.417746
	3	−66.865288	−53.072853	−13.792435	11.982115
5	1	−50.337765	−44.250916	−6.086848	96.381271
6	1	−45.127968	−42.331528	−2.796439	9.279520
7	1	−72.713211	−51.605087	−21.108124	15.293597
10	1	−44.027946	−34.921326	−9.106619	8.631289
	2	−42.797626	−35.534809	−7.262817	7.740744
22	1	−46.489151	−41.222374	−5.266776	9.209332

Grid_vdw uses the grid method to calculate the van der Waals force contribution; Grid_es uses grid method to calculate the contribution of electrostatic force; Grid Score = grid_vdw + Grid_es; total score Int_energy intramolecular energy.

**Table 3:** Docking of ligand with receptor HDAC2 (unit: kcal/mol).

Compound	Pose	Grid Score	Grid_vdw	Grid_es	Int_energy
Ligand2	1	−67.1829	−44.7886	−22.3944	13.1161
	2	−62.4968	−48.8023	−13.69444	16.3890
	3	−62.2309	−37.3300	−24.90084	35.4859

**Table 4:** Compound **4** effect on HepG2 cell cycle of human.

Group	G1 (%)	S (%)	G2 (%)
CON	48.06	35.8	16.14
1 μM	55.92	32.46	11.62
3 μM	58.06	32.36	9.57
12 μM	63.84	18.76	17.4

*p* < 0.01, compared with control group.

**Table 5:** Compound **4** on the HepG2 cells’ apoptosis rate.

Group	UL (%)	UR (%)	LL (%)	LR (%)	Apoptosis (%)
CON	0.63	2.44	94.78	2.16	4.6
1 μM	0.09	7.80	81.93	10.17	17.97
3 μM	0.12	13.92	70.57	15.39	29.31
12 μM	0.08	20.00	57.52	22.40	42.40

**Table 6:** Compound **4** effect on HepG2 mitochondrial membrane potential.

Group	Green Fluorescence (%)
CON	5.80
1 μM	13.16
3 μM	27.03
12 μM	49.32

**Table 7:** Compound **4** effect on the cellular calcium of HepG2.

Group	Mean
CON	31.00
1 μM	56.94
3 μM	83.26
12 μM	124.72

## Supplementary Materials

Supplementary materials can be found at http://www.mdpi.com/1422-0067/19/10/3184/s1.

## References

[1] Jiang X. (2006). Effects of Docetaxel on HepG2 Proteome in Human Hepatoma Cell.

[2] Fraser M., Leung B., Jahani-Asl A., Yan X., Thompson W.E., Tsang B.K. (2003). Chemoresi stance in human ovarian cancer: The role of apoptotic regulators. Reprod. Biol. Endocrinol..

[3] Mizutani H., Tada-Oikawa S., Hiraku Y., Kojima M., Kawanishi S. (2005). Mechanism of apoptosis induced by doxorubicin through the generation of hydrogen peroxide. Life Sci..

[4] Watanabe H., Fujimoto A., Takahara A. (2013). Characterization of catechol-containing natural thermosetting polymer “urushiol” thin film. J. Polym. Sci. Part A Polym. Chem..

[5] Xie Y., Zhang J., Liu W., Xie N., Feng F., Qu W. (2016). New urushiols with platelet aggregation inhibitory activities from resin of Toxicodendron vernicifluum. Fitoterapia.

[6] Hong S.H., Suk K.T., Choi S.H., Lee J.W., Sung H.T., Kim C.H., Kim E.J., Kim M.J., Han S.H., Kim M.Y. (2013). Anti-oxidant and natural killer cell activity of Korean red ginseng (*Panax ginseng*) and urushiol (*Rhus vernicifera* Stokes) on non-alcoholic fatty liver disease of rat. Food Chem. Toxicol..

[7] Ryckewaert L., Sacconnay L., Carrupt P.A., Nurisso A., Simoes-Pires C. (2014). Non-specific SIRT inhibition as a mechanism for the cytotoxicity of ginkgolic acids and urushiols. Toxicol. Lett..

[8] Cha H.S., Shin D.H. (2016). Antibacterial capacity of cavity disinfectants against Streptococcus mutans and their effects on shear bond strength of a self-etch adhesive. Dent. Mater. J..

[9] Cho J.Y., Park K.Y., Kim S.J., Oh S., Moon J.H. (2015). Antimicrobial activity of the synthesized non-allergenic urushiol derivatives. Biosci. Biotechnol. Biochem..

[10] Halloran L. (2014). Developing Dermatology Detective Powers: Allergic Contact Dermatitis. J. Nurse Pract..

[11] Hachisuka J., Ross S.E. (2014). Understanding the switch from pain-to-itch in dermatitis. Neurosci. Lett..

[12] Lu R., Miyakoshi T. (2015). Lacquer Chemistry and Applications.

[13] Coifman R., Yang C. (2014). Poster 1019: Novel allergy vaccine delivery system for poison ivy urushiol (PI) and peanut (PN). World Allergy Organ. J..

[14] Hong M., Kim S.W., Han S.H., Kim D.J., Suk K.T., Kim Y.S., Kim M.J., Kim M.Y., Baik S.K., Ham Y.L. (2015). Probiotics (*Lactobacillus rhamnosus* R0011 and acidophilus R0052) reduce the expression of toll-like receptor 4 in mice with alcoholic liver disease. PLoS ONE.

[15] Lin Y.C., Wang F.F. (2008). Mechanisms underlying the pro-survival pathway of p53 in suppressing mitotic death induced by adriamycin. Cell Signal.

[16] Jantova S., Letasiova S., Ovadekova R., Muckova M. (2006). Cytotoxic/antiproliferative effects of new [1,2,4]triazolo[4,3-c] quinazolines in tum or cell lines H eLa and B16. Neoplasma.

[17] Deprez-Poulain R., Hennuyer N., Bosc D., Liang W.G., Enee E., Marechal X., Charton J., Totobenazara J., Berte G., Jahklal J. (2015). Catalytic site inhibition of insulin-degrading enzyme by a small molecule induces glucose intolerance in mice. Nat. Commun..

[18] Marzano C., Pellei M., Colavito D., Alidori S., Lobbia G.G., Gandin V., Tisato F., Santini C. (2006). Synthesis, characterization, and invitro antitumor properties of tris(hydroxymethyl) phosphine copper(I) complexes containing the new bis(1,2,4-tri-azol-1-yl) acetate ligand. J. Med. Chem..

[19] Bang C.S., Hong S.H., Suk K.T., Kim J.B., Han S.H., Sung H., Kim E.J., Kim M.J., Kim M.Y., Baik S.K. (2014). Effects of Korean Red Ginseng (*Panax ginseng*), urushiol (*Rhus vernicifera* Stokes), and probiotics (*Lactobacillus rhamnosus* R0011 and *Lactobacillus acidophilus* R0052) on the gut-liver axis of alcoholic liver disease. J. Ginseng Res..

[20] Suk K.T., Baik S.K., Kim H.S., Park S.M., Paeng K.J., Uh Y., Jang I.H., Cho M.Y., Choi E.H., Kim M.J. (2011). Antibacterial Effects of the Urushiol Component in the Sap of the Lacquer Tree (*Rhus verniciflua* Stokes) on Helicobacter pylori. Helicobacter.

[21] Tretyakova E.V., Smirnova I.E., Kazakova O.B., Tolstikov G.A., Yavorskaya N.P., Golubeva I.S., Pugacheva R.B., Apryshko G.N., Poroikov V.V. (2014). Synthesis and anticancer activity of quinopimaric and maleopimaric acids’ derivatives. Bioorg. Med. Chem..

[22] Tretyakova E.V., Smirnova I.E., Salimova E.V., Odinokov V.N. (2015). Synthesis and antiviral activity of maleopimaric and quinopimaric acids’ derivatives. Bioorg. Med. Chem..

[23] Zhou H., Wang C., Ye J., Chen H., Tao R. (2017). Design, virtual screening, molecular docking and molecular dynamics studies of novel urushiol derivatives as potential HDAC2 selective inhibitors. Gene.

[24] Zhou H., Wang C., Deng T., Tao R., Li W. (2018). Novel urushiol derivatives as, H.D.AC8 inhibitors: Rational design, virtual screening, molecular docking and molecular dynamics studies. J. Biomol. Struct. Dyn..

[25] Ning C., Bi Y., He Y., Huang W., Liu L., Li Y., Zhang S., Liu X., Yu N. (2013). Design, synthesis and biological evaluation of di-substituted cinnamic hydroxamic acids bearing urea/thiourea unit as potent histone deacetylase inhibitors. Bioorg. Med. Chem. Lett..

[26] Hildmann C., Riester D., Schwienhorst A. (2007). Histone deacetylases—An important class of cellular regulators with a variety of functions. Appl. Microbiol. Biotechnol..

[27] Drummond D.C., Noble C.O., Kirpotin D.B., Guo Z., Scott G.K., Benz C.C. (2005). Clinical development of histone deacetylase inhibitors as anticancer agents. Annu. Rev. Pharmacol. Toxicol..

[28] Ewa Augustin a A.M.-R. (2006). Induction of G2/M phase arrest and apoptosis of human leukemia cells by potent antitumor triazoloacridinone C-1305. Biochem. Pharmacol..

[29] Guo L., Li Z.S., Wang H.L. (2006). Carboxyamido-triazole inhibits proliferation of human breast cancer cells via G2/M cell cycle arrest and apoptosis. Eur. J. Pharmacol..

[30] Rezayati S., Sheikholeslami-Farahani F., Rostami-Charati F., Abad S.A. (2015). One-pot synthesis of coumarine derivatives using butylenebispyridinium hydrogen sulfate as novel ionic liquid catalyst. Res. Chem. Intermed..

[31] Potdar M.K., Mohile S.S., Salunkhe M.M. (2001). Coumarin syntheses via Pechmann condensation in Lewis acidic chloroaluminate ionic liquid. Tetrahedron Lett..

[32] Khandekar A.C., Khadilkar B.M. (2002). Pechmann Reaction in Chloroaluminate Ionic Liquid. Synlett.

[33] Futagawa M., Wedge D.E., Dayan F.E. (2002). Physiological factors influencing the antifungal activity of zopfiellin. FEBS Lett..

[34] Hosoe T., Fukushima K., Itabashi T. (2004). The abeolute structures of dihydroepiheveadride, as characteristic antifungal agent filamentous fimi, and its related compounds from unidentified fungus, I.F.M52672. Eterocycles.

[35] Dörwald F.Z. (2014). Side Reactions in Organic Syntheis, I.I.: Aromatic Substitutions.

[36] Qi Z., Wang C., Jiang J. (2018). Synthesis and Evaluation of C15 Triene Urushiol Derivatives as Potential Anticancer Agents and, H.D.AC2 Inhibitor. Molecules.

[37] Xu Y. (2015). Study of the influence of taxol on hepatocytes of rats in vitro. J. Jinan Univ..

[38] Schiff P.B., Fant J., Horwitz S.B. (1979). Promotion of microtubule assembly in vitro by taxol. Nature.

[39] Parness J., Horwitz S.B. (1981). Taxol binds to polymerized tubulinin vitro. J. Cell Biol..

[40] Manfredi J.J., Parness J., Horwitz S.B. (1982). Taxol binds to cellular microtubules. J. Cell Biol..

[41] Schiff P., Horwitz S.B. (1980). Taxol stabilizes microtubules in mouse fibmblaSt cells. Proc. Natl. Acad. Sci. USA.

[42] Pettersen E.F., Goddard T.D., Huang C.C., Couch G.S., Greenblatt D.M., Meng E.C., Ferrin T.E. (2004). UCSF Chimera—A visualization system for exploratory research and analysis. J. Comput. Chem..

[43] Jakalian A., Bush B.L., Jack D.B., Bayly C.I. (2000). Fast, Efficient Generation of High-Quality Atomic Charges. AM1-BCC Model: I. Method. J. Comput. Chem..

[44] Jakalian A., Jack D.B., Bayly C.I. (2002). Fast, efficient generation of high-quality atomic charges. AM1-BCC model: II. Parameterization and validation. J. Comput. Chem..

[45] Lang P.T., Brozell S.R., Mukherjee S., Pettersen E.F., Meng E.C., Thomas V., Rizzo R.C., Case D.A., James T.L., Kuntz I.D. (2009). DOCK 6: Combining techniques to model, RNA—Small molecule complexes. RNA.

[46] Mukherjee S., Balius T.E., Rizzo R.C. (2010). Docking Validation Resources: Protein Family and Ligand Flexibility Experiments. J. Chem. Inf. Model..

[47] Tret’yakova E.V., Salimova E.V., Parfenova L.V., Odinokov V.N. (2016). Synthesis and modifications of alkyne derivatives of dihydroquinopimaric, maleopimaric, and fumaropimaric acids. Russ. J. Organ. Chem..

[48] Shen Q.-K., Liu C.-F., Zhang H.-J., Tian Y.-S., Quan Z.-S. (2017). Design and synthesis of new triazoles linked to xanthotoxin for potent and highly selective anti-gastric cancer agents. Bioorg. Med. Chem. Lett..

[49] Zareyee D., Serehneh M. (2014). Recyclable CMK-5 supported sulfonic acid as an environmentally benign catalyst for solvent-free one-pot construction of coumarin through Pechmann condensation. J. Mol. Catal. A Chem..

[50] Chen D.Z., Jing C.X., Cai J.Y., Wu J.B., Wang S., Yin J.L., Li X.N., Li L., Hao X.J. (2016). Design, Synthesis, and Structural Optimization of Lycorine-Derived Phenanthridine Derivatives as Wnt/beta-Catenin Signaling Pathway Agonists. J. Nat. Prod..

