# Supplementary material for: Novel C15 Triene Triazole, D-A Derivatives Anti-HepG2, and as HDAC2 Inhibitors: A Synergy Study
Source: Int J Mol Sci. 2018 Oct 16;19(10):3184. doi: 10.3390/ijms19103184 (PMC6214004; doi:10.3390/ijms19103184)

Novel C15 Triene Triazole, D-A Derivatives Anti-HepG2 and as HDAC2 Inhibitors, Synergy Study

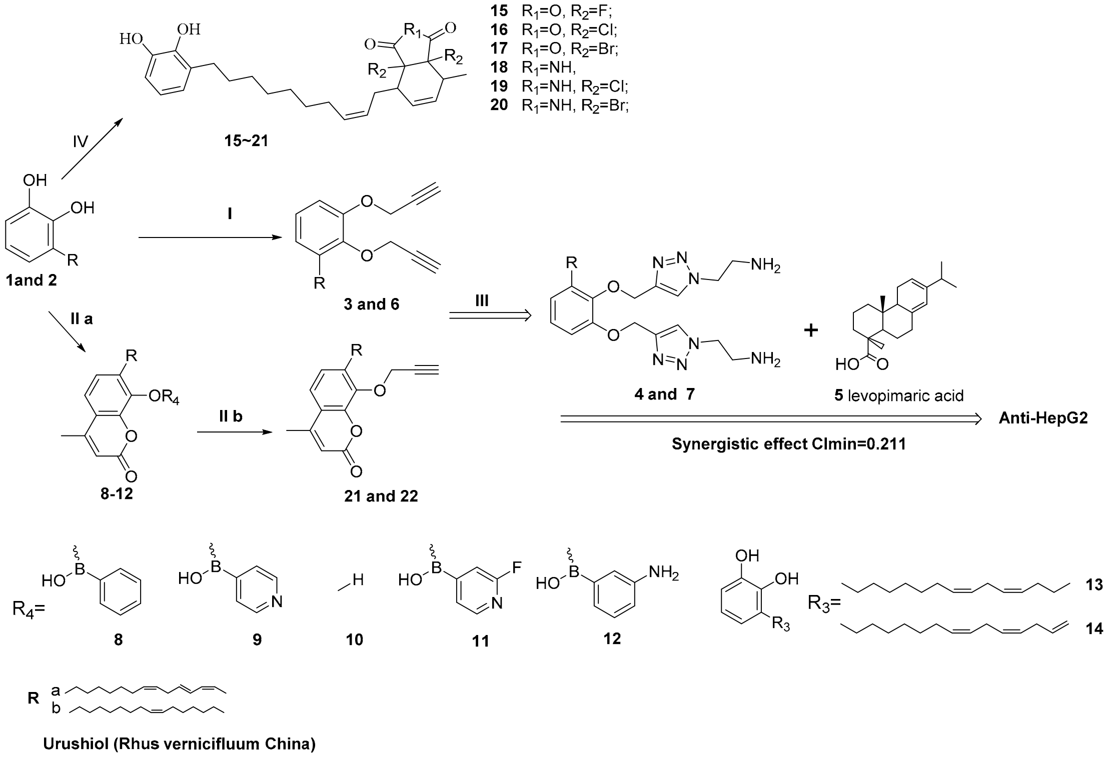

Scheme S1. I. 4.0 eq K_2CO_3, 2.5 eq propargyl bromide, dry acetone, 60℃, 24 h; II. a. EA, p-toluene sulfonic acid, r.t. 12 h; b. as I condition; III. 1.3 eq 2-Azidoethyl- amine, $CuSO_4$, sodium ascorbate solution, H_2O/*t*-BuOH solution; IV. Reactants (ratio 0.5:1) were mixed in the high temperature environment and stirred at 160°C for 2 h, stopped the reaction and cooled to room temperature to obtain the target organic synthesis product (GCMS,^1H/^{13}C NMR in Supporting information).

1. Synergy

Material

DMSO and MTT were purchased from Sigma, USA, and levopimaric acid was dissolved for injection and stored at 4℃ darkness for use. DMEM medium and fetal bovine serum were purchased from Gibco, USA. HepG2 cells were purchased from Jiangsu Keygentec Biotechnology Co., Ltd.

Method

Cell culture: Human hepatoma cell line HepG2 cells were cultured in DMEM medium that contained 10% inactivated fetal bovine serum, and sub-cultivated at 37°C, 5% CO_2 atmosphere. Logarithmic growth cells were used for the following work.

Statistical method

*SPSS 17.0 Statistical software were used for data processing and statistical scoring analysis. Display the experimental data as the mean ± standard deviation (x ± s). Satisfied the normal distribution and variance by the test of normality and variance homogeneity. Applied Univariate ANOVA to compare the homogeneity.

Joint index determination

CI-Fa relationship and HepG2 apoptosis (CI=0.211) Statistical analysis: A p<0.05 was considered significantly. All analyses were carried out with SPSS 19.0 software (SPSS, Chicago, IL, USA). (1) Joint index determination: the index of cooperation was calculated by using CalcuSyn software (Combi-nation index, CI) The CI < 1 represented that they have synergistic effect while CI ≈ 1 and CI > 1 indicated additive and antagonism respectively. (2) Statistical method SPSS 17.0 statistical software was used for statistical analysis. The measurement data material of the experimental were described by x±s. Two samples were compared with the t test. The difference was significant statistically ($p < 0.05$).

MTT assay detect cell proliferation

MTT assay was used to determine the inhibitory effect on the proliferation of HepG2 cells of levopimaric acid. Compound **4** and levopimaric acid were combined with three groups below. The cells in the logarithmic growth phase were inoculated into 96-well culture plates at 3.5×10^3 cells/well, 0.2 ml per well, and incubated for 72 h with different concentrations of levopimaric acid. Then, we continually cultured it for 4 h after mixing fresh MTT working solution 20 μL (final concentration was 0.5 mg/mL), and carefully discarded the supernatant, added 1.5 mL of DMSO per well and shook up. The absorbance (A) at the wavelength of 490 nm was measured by a microplate reader. The cell proliferation inhibition rate was calculated according to the following formula=[(negative control group A value - blank group A value) - (experimental group A value - blank group A value)]/ (Negative control group A value - blank group A value) × 100%.

Table S1 Compound 4 and levopimaric acid anti-HepG2 by single drug.

**Concentration** /μM	**4** Inhibitory rate/%	**Concentration** /μM	**Levopimaric acid** Inhibitory rate/%
200	99.99	150	91.45
100	99.25	75	73.41
50	98.35	37.5	47.21
25	73.88	18.75	44.58
12.5	68.96	9.375	32.14
6.25	65.22	4.687	13.75
3.13	56.85	2.343	9.45
1.56	39.36	1.171	5.45
0.78	21.19	0.585	4.12

Combination therapy group

(Levopimaric acid + compound **4** (1st)) **A** group: (7.9, 3.95, 1.98, 0.988, 0.494, 0.247) μM of levopimaric were incubated with (29, 14.65, 7.32, 3.66, 1.83, 0.916) μM of compound **4** for 72 h, respectively.

(Levopimaric acid + compound **4** (2st)) **B** group: the concentrations of levopimaric above were incubated with (14.65, 7.32, 3.66, 1.83, 0.916, 0.458) μM of compound **4** for 72 h respectively;

(Levopimaric acid + compound **4** (3st)) **C** group: the concentrations of levopimaric above were incubated with (7.32, 3.66, 1.83, 0.916, 0.458 and 0.229) μM of compound **4** for 72 h, respectively.

Detection of cell proliferation by MTT

MTT assay was used to determine the inhibitory effect of the sample on the proliferation of HepG2 cells. The tumor cells of logarithmic growth stage were inoculated with 3.5×10^3/well in 96 well culture plate. 0.2 ml/pore system was cultured for 72 h with different concentrations of samples. Then 20 μL (final concentration 0.5 mg/mL) of MTT were added to each pore for 4 h. The supernatant was carefully adsorbed, and 0.15 mL DMSO was added to each pore, and oscillatory mixing was carried out. The absorbance (A) value at 490 nm wavelength was measured by enzyme labeling instrument. The inhibition rate of cell proliferation was calculated as follows: [(A value of negative control group - A value of blank group) - (A value of experimental group A value of blank group)]/(negative pair

group A-A value of blank group) × 100. The statistical analysis was carried out by SPSS 17.0 statistical software. The measured data were described by x±s, and the mean of the two samples was compared with t test (P < 0. 05).

2. HDAC2 expression

Materials

HDAC2, with a molecular weight of 60 kD (1 KD=0.9921 Ku), was purchased from Santa Cruz Company, Santa Cruz, USA. The 60 kD HDAC2 standard was diluted with buffer solution and 1% SDS at 2 μg/ml, then stored at -20℃. ECL membrane and Polyacrylamide gel were purchased from AmershamLife Science Company, Amersham, USA. ECL membrane elution buffer was purchased from PIERCE Company, Colorado, USA. The cell culture medium and its additive were purchased from Gibco Company, New York, USA. Full-range rainbow Markers and a full molecular weight protein standard were purchased from Amersham Biosciences, USA. 10% SDS polyacrylamide gel was prepared by myself. FACS Calibur flow cytometry (BD Company, New Jersey, USA). The samples spotted or dotted with 20 μL of each sample, 10 μL of HDAC2 standard sample with 60 kD. Full molecular weight standard protein sample spotted or dotted with 10 μL, electrophoresis for 1.5 h under 100 V voltage. The white matter was transferred to ECL film (100 V, 1 h), blocked with PBST containing 5% skim milk powder (PBS solution containing 0.5% Tween-20) at room temperature (r.t) for 2 h, then diluted with compounds **4** (dilute concentration for 1: 100, 1: 200, 1: 500 by PBST containing 2% skim milk powder) at 4℃ overnight with 100 r/min. Above mixture solution was washed by PBST solution for 3 times, then reacted with horseradish peroxidase labeled goat anti-rat Ig (1: 7500) for 1.5 h at room temperature. The chemiluminescence kit's chromogenic reaction and exposure to detect the expression of HDAC2. The used ECL membrane was washed with eluent at room temperature for 15 min, then stained with Actin antibody (1: 10000) and detected.

3. Flow cytometry

The HepG2 cells were cultured in complete medium (90% DMEM + 10% FBS) with saturated humidity incubator at 37℃, 5% CO_2, which were provided by Jiangsu Kaiji Biotechnology Co., Ltd, China.

Main reagents and consumables: Cell culture bottle (FALCON 353014, USA), Penicillin/streptomycin solution (Jiangsu Kaiji Biotechnology Co., Ltd., China), 0.25% Tripsin-EDTA (Jiangsu Kaiji Biotechnology Co., Ltd., China), DMEM (GIBCO 11965-084, USA), MEM (GIBCO 12571-063, USA), FBS (Gibco 10082147, USA), 6 well cell culture plate (Corning Incorporated 3516), MTT cell proliferation and cytotoxicity test kit (KGA311, Jiangsu Kaiji Biotechnology Development Co., Ltd., China) Cell cycle detection kit (KGA512, Jiangsu Kaiji Biotechnology Development Co., Ltd., China), Apoptotic mitochondrial membrane potential detection kit JC-1 (KGA604, Jiangsu Kaiji Biotechnology Development Co., Ltd., China), Annexin V-FITC/PI double staining cell apoptosis detection kit (KGA108, Kaiji Biotechnology Development Co., Ltd., Jiangsu, China), Ca^{2+} GPCR analysis-calcium ion indicator probe Fluo-3, AM (KGAF023, Kaiji Biotechnology Development Co., Ltd., Jiangsu, China).

Cell resuscitation

Firstly, removed the cryopreservation tube from the liquid nitrogen, and immediately plunged it into the prepared water that was between 37℃ and 40℃, until the cryopreservation liquid completely dissolved. Then, transferred cell cryopreservation suspension to a centrifuge tube, added about 5 mL medium, and mix them gently. After that, centrifuged cell suspension at 800 ~ 1000 r/min for 5 min, and abandoned the above liquor. Finally, joined the cell pellet into culture medium and stir them lightly. After transferring the cell suspension to culture flask, replenished the medium.

Cell passage

Firstly, sucked out the original medium when the cell coverage in the culture bottle had reached 80% ~ 90%. Secondly, added appropriate trypsin (0.25%) to digest the cells for 1~2 min until they became round. Mixed the equal volume of serum-containing medium to terminate the digestion. Thirdly, use pipette to percuss cells and make them suspend. Then, sucked the cells into a 15 mL centrifuge tube and centrifuge them for 5 min. At last, dropped the supernatant, add 1~2 mL medium to suspend the cells, and cultivated them in the culture bottle.

Cell cryopreservation

The first step was to add suitable trypsin digestion cells and collected the cell suspension. Centrifuged the suspension at 1000 rpm for 5 min in the tube, and then abandoned the supernatant. Secondly, added cryoprotectant to the cell sediment and mixed them lightly until the cell density was 1×10^6~1×10^7/ml. Later, divided them into 1~1.5 ml per tube, tightened the cap and made a mark on surface including the cell code and the freezing date. In the end, cooled the cells in following order: room temperature→ 4℃ (20 min) → the freezer (30 min) → low temperature refrigerator (-30℃ for 1 h) →gaseous nitrogen (30 min) →the liquid nitrogen.

Cell proliferation measured by MTT

First of all, digested the cells and counted them. Added 100 μL cell suspension (3×10^4 /ml) into each hole of the 96 holes cell culture plate. Secondly, placed the 96 holes cell culture plate in a 5% CO_2 culture box for 24 h at 37℃. Then, used the medium to dilute the drug into the desired working fluid concentration, added 100 μL per hole to the corresponding drug medium, and established a negative control group as well as a positive control group (paclitaxel, 20 ug/ml). Next, placed 96 holes cell culture plate at 37℃ and cultivated the cells in a 5% CO_2 incubator for 72 h. After treating 96 holes board with MTT staining, estimated the OD value:

a. Added 20 μL MTT (5 mg/ml) per hole, and cultivated cells in the box for 4 h.

b. Discarded the supernatant, added 150 μL DMSO in each hole, and mixed them gently after treatment with shaker for 10 min.

c. Under 490nm ultraviolet wavelength, read the OD value from ELISA.

Finally, figured up the inhibition rate of each group

*The inhibition rate (%) = (negative control group OD value - experimental group OD value) / negative control group OD value ×100%.

Detection of cell cycle by PI single staining

Firstly, digested the cells of logarithmic growth phase and inoculated them into six-well plates. The next day, joined the corresponding drug-containing medium after the cells adhered. And established the negative control group at the same time. After 72 h, use 0.25% pancreatin (not contain EDTA) to digest the cells. Thirdly, applied PBS to scrub the cells (centrifuge them at 2000 rpm for 5min), and collected 5×10^5 cells. Then, used a volume fraction of 70% ethanol to fix the single cell suspension for 2 h (or overnight), and stored it at 4℃. Before washing, employed PBS to remove the fixative solution (if needed, filter cell suspension through 200 mesh). Added 100 mL RNase A, and made it in water bath at 37℃ for 30 min. Later, placed it at 4℃ for 30 min without light. The final step was to check the machine, recorded the red fluorescence at the excitation wavelength of 488 nm.

Detection of apoptosis by Annexin-V FITC/PI double staining

Firstly, inoculated the logarithmic growth cells into the six-hole plate. The next day, after the cells adhered to the wall, joined the corresponding medicine-containing medium, and meanwhile set up the negative control group. After 72 h treatment, the cells were digested by using 0.25% trypsin (excluding EDTA) and then collected. Then, used PBS to wash the cells twice (centrifuge them at 2000 rpm for 5 min), and collected 5×10^5 cells. The next step was to add 500 μL Binding Buffered to make cells suspend. After cells were mixed with 5 μL Annexin V-FITC, stirred them again with 5 μL PI. At

last, treated the mixture at room temperature for 5~15 min without light, and utilized flow cytometry to detect the apoptosis.

Detection of mitochondrial membrane potential by JC-1 staining

First of all, inoculated the logarithmic growth cell digestion into the six-hole plate. The next day, added the corresponding drug-containing medium after the cells adhered to the wall, and built up a negative control group as well. Secondly, collected the cell which had been washed by PBS (centrifuge them at 2000 rpm for 5 min), and changed the cell concentration into 1×10^6/ml. Added 900 μL sterilized deionized water to 100 μL 10×Incubation Buffer, and diluted them into 1×Incubation buffer. Stirred well and then preheated them to 37℃. Absorbed the 500 μL 1×Incubation buffer, added 1 μL JC-1 to prepare the JC-1 working fluid. Next, incubated the cells which had been treated with the above fluid in a 5% CO_2 incubator at 37℃ for 15~20 min. Then, collected the cells by centrifugation at room temperature for 5 min, and washed them twice with 1×Incubation Buffer. 500μL 1×Incubation Buffer of resuspension cells were absorbed, and did the computer test to attain the final result.

Calcium content detection

Firstly, inoculated the logarithmic growth cells into the six-hole plate. The next day, after the cells adhered to the wall, added the corresponding drug-containing medium and set up the negative control group at the same time. After 72 h of treatment, digested the cells with 0.25% trypsin (excluding EDTA), and collected 5×10^5 cells which had been washed twice by PBS (centrifuge them at 2000 rpm for 5 min). Added 5 μM Fluo-3 probe and mix them. After treatment at 37℃ for 30 min without light, did the computer testing to detect calcium content.

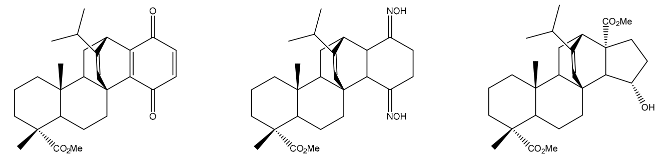

a b c

Figure S1 Levopimaric acid derivatives.

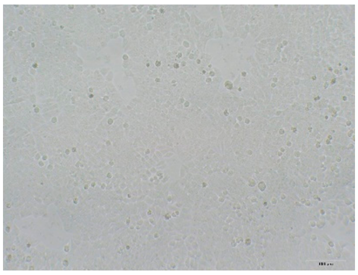

a

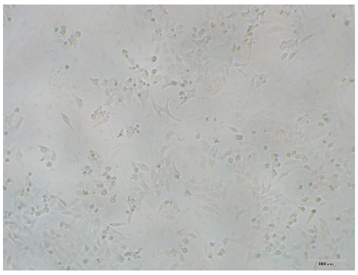

b

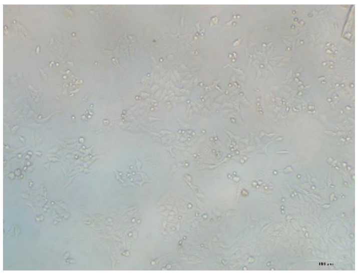

c

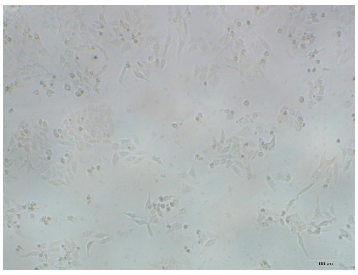

d

a HepG2 negative control； b 6.25 μM； c 25 μM; d 50 μM;

Figure S2 Proliferation of HepG2 cells by compound 4 at different concentrations.

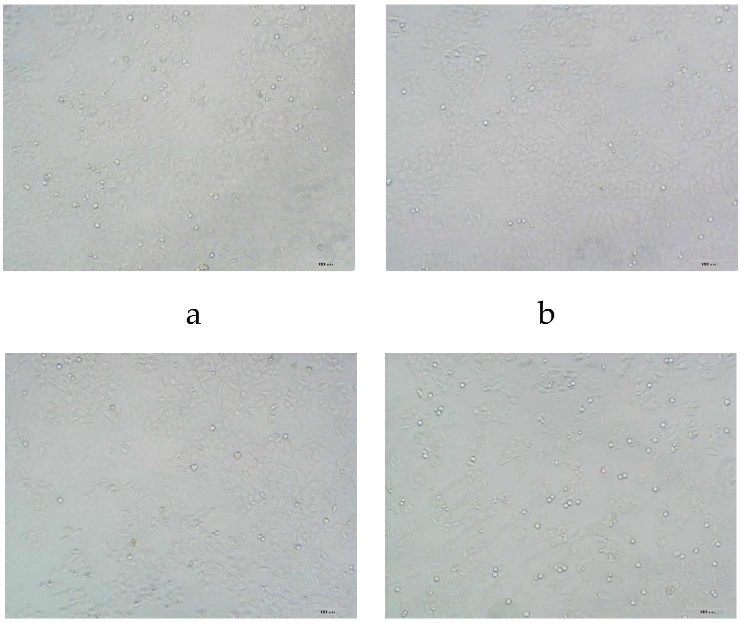

a LO2 negative control; b 25 μM； c 50 μM; d 200 μM;

Figure S3 Proliferation of LO2 cells by compound 4 at different concentrations.

A series of novel C15 triene urushiol (3-((8Z,11E,13Z)-pentadeca-8,11,13- trien-1-yl) benzene-1,2-diol) derivatives were designed by introducing pechmann structure and -F, -Cl, -Br, nitro substituents with different electronic properties into its alkyl side chain, triazolyl functional group in its aromatic oxide.(Scheme S1) Their chemical structures were determined based on the analysis of NMR spectroscopic and mass spectrometric data as follows:

Compound **1:** Triene Urushiol (3-((8Z,11E,13Z)-pentadeca-8,11,13-trien-1-yl) benzene-1,2- diol) ^{1}H NMR (400 MHz, $CDCl_3$) δ 7.00 (t, J = 7.6 Hz, 1H), 6.63 (d, 1H), 6.55 (d, 1H), 6.25 (t, 1H), 5.88 (t, 1H), 5.53 (m, 1H), 5.33–5.26 (m, 3H) 2.74(Ar-CH_2), 2.42, 1.92, 1.63, 1.47, 1.23(-CH_2-), 1.20(-CH_2-), 1.17(-CH_2-), 1.15(-CH_2-). ^{13}C NMR (101 MHz, $CDCl_3$) δ 155.13, 144.33, 131.83, 130.59, 128.91, 128.84, 126.34, 125.07, 123.87, 120.27, 114.89, 112.07. ESIMS m/z 314.2 $[M]^+$.

Compound **2:** Mono-olefins Urushiol ((Z)-3-(hexadec-9-en-1-yl) benzene-1,2-diol) ^{1}H NMR (400 MHz, $CDCl_3$) δ 6.71 (t, 3H), 5.35(t, 1H), 5.09(d, 1H), 2.60 (t, 2H), 1.61 (m, 2H), 1.31-1.28(16H), 0.88(-CH_3). ^{13}C NMR (101 MHz, $CDCl_3$) δ 142.86, 141.72, 132.20, 131.00, 129.29, 126.68, 125.38, 124.22, 121.98, 120.04, 112.82, 30.47, 29.60, 29.46, 29.36, 29.27, 29.08, 27.08, 27.01, 13.15(-CH_3). ESIMS m/z 318.3 $[M]^+$.

The compound **5** crude levopimaric acid was obtained by using previous amine salt method [51]. The novel extraction optimum condition was at 100% ethanol concentration, 0℃ crystallization with, 20:1 (mL/g) volume ratio of ethanol to crystallization mass and 2 times recrystallizations. Some fine crystal shape and uniform rectangular transparent crystal were generated, and the levopimaric acid purity was above 96%.

Compound **5:** ^{1}H NMR (400 MHz, $CDCl_3$) δ7.26 (s, 1H), 5.53 (s, 1H), 5.14 (s, 1H), 2.43–2.35 (m, 1H), 2.33 (s, 2H), 2.29 (dd, J = 4.7, 2.1 Hz, 1H), 2.14 (s, 3H), 1.94 (s, 1H), 1.91 (s, 1H), 1.80 (s, 1H), 1.76 (s, 1H), 1.73 (d, J = 5.3 Hz, 1H), 1.64 (s, 1H), 1.61 (d, J = 3.2 Hz, 1H), 1.57 (s, 3H), 1.38 (s, 1H), 1.35 (s, 1H), 1.17 (s, 1H), 0.98 (s, 1H), 0.96 (s, 3H), 0.91 (s, 3H). ^{13}C NMR (101 MHz, $CDCl_3$) δ 184.99, 145.26, 135.41, 122.22, 120.36, 50.74, 46.29, 44.73, 38.14, 37.07, 34.79, 34.34, 27.23, 25.52, 22.38, 21.28, 20.70, 17.96, 16.60, 13.91. ESIMS m/z 301.2 $[M-H]^+$.

Syntheses of "Model Molecules". Syntheses of Compounds **3** and **6** [47, 48]. Triene or mono-olefins uruhsiol (0.5 mmol) was dissolved in dry acetone (3 mL), and K_2CO_3 (0.5 mmol) and propargyl bromide (100 μL, 5.25 mmol) were added. The mixture was stirred at 60°C for 24 h and quenched with H_2O (50 mL) in 0℃ bath. The solution was evaporated to remove acetone and extracted with

DCM (2 × 30 mL). The organic layer was washed with saturated $NaHCO_3$ and brine, dried over $MgSO_4$, filtered, and finally concentrated. The residue was purified by column chromatography with $CHCl_3$-MeOH (100:5) to offer compounds **3** and **6** respectively.

Compound **3:** (1-((8Z,11E,13Z)-pentadeca-8,11,13-trien-1-yl)-2,3-bis (prop-2-yn-1-yloxy) benzene). Yellow liquid. Yield: 77 mg, 42%. ^{1}H NMR (400 MHz, $CDCl_3$) δ 6.92 (t, 1H), 6.78 (dd, 2H), 6.27 (t, 1H), 5.90 (t, 1H), 5.56 (m, 1H), 5.33(m, 3H), 4.65 (t, 4H, -*CH*$_2$-HC≡C), 2.03 (2H, -C-O-), 1.28. ^{13}C NMR ($CDCl_3$, 101 MHz) δ 158.69 (2C), 129.96 (CH), 107.84 (2CH), 102.39 (CH), 78.49 (2-C), 75.73 (2≡*CH*), 55.82 (2-*CH*$_2$); ESIMS m/z 429.0 [M + K]$^+$.

Compound **6:** ((Z)-1-(hexadec-9-en-1-yl)-2,3-bis(prop-2-yn-1-yloxy) benzene). Yellow liquid. Yield: 40 mg, 26%. ^{1}H NMR (400 MHz, $CDCl_3$) δ7.06 (d, J = 4.7 Hz, 1H), 6.94-6.92(dd,2H), 5.70 (m, 1H), 5.48 (m, 1H), 4.80 (d, J = 2.6 Hz, 4H), 2.87(t,2H), 1.39. ^{13}C NMR (101 MHz, $CDCl_3$) δ 131.14, 129.38, 126.79, 125.55, 124.08, 123.08, 112.18, 75.60(*2C, -C≡C-*), 74.85(*2C, -C≡C-*), 60.15(*1C,-C-O-*), 56.59(*1C,-C-O-*), 31.78, 30.61, 30.59, 30.14, 29.69, 29.63, 29.54, 29.38, 29.22, 28.94, 27.22, 27.17, 25.54, 22.66, 13.26. ESIMS m/z 374.2 [M-C_3H_7+Na]$^+$.

Syntheses of Compounds **4** and **7**. Compounds **3** (77 mg, 0.2 mmol) or compound **6** (40 mg, 0.1 mmol) was added to compound azide propylamine (22 μL, 0.25 mmol) in H_2O/*t*-BuOH (2 mL, 1:1), followed by adding $CuSO_4$ (3.0 mg) and a sodium ascorbate solution (50 μL, 1 M solution). The mixture was stirred for 15 h at room temperature (r.t) and concentrated in vacuum; the resultant residue was purified by column chromatography with $CHCl_3$-MeOH (50:1) to yield compounds **4** and **7**, respectively.

Compound **4:** (2,2'-((((3-((8Z,11E,13Z)-pentadeca-8,11,13-trien-1-yl)-1,2-phenylene) bis (oxy)) bis(meth-ylene)) bis(1H-1,2,3-triazole-4,1-diyl)) bis(ethan-1-amine)) Yellow liquid. Yield: 33 mg, 27%. ^{1}H NMR (400 MHz, $CDCl_3$) δ 7.47-7.45(*trizole, -CH=*),6.58-6.62(Ar, m, 3H), 6.28(t, 1H), 5.90(t, 1H), 5.56(m, 1H), 5.32(m,3H), 5.27(*s, 4H, -O-CH*$_2$*-*), 4.69(*-CH*$_2$*-*), 4.06, 4.04, 3.87, 3.85, 1.54(t, 4H, -NH_2). ^{13}C NMR (101 MHz, $CDCl_3$) δ 143.71, 142.71, 132.35, 131.18, 129.94, 129.93, 126.89, 125.63, 121.62, 119.56, 112.69, 77.42, 76.90, 60.45(*-C-N*), 31.86, 30.68, 29.90, 29.81, 29.76, 29.64, 29.54, 29.36, 29.05, 27.28, 22.74, 21.03. ESIMS m/z 429.0 [M– $C_{10}H_{16}$]$^+$

Compound **7:** ((Z)-2,2'-((((3-(hexadec-9-en-1-yl)-1,2-phenyl ene)bis(oxy))bis(methylene)) bis(1H-1,2,3-triazole-4,1-diyl))bis (ethan-1-amine)) Yellow liquid. Yield: 31 mg, 73%. ^{1}H NMR (400 MHz, $CDCl_3$) δ7.54-7.52 (*trizole, -CH=, 2H*), 7.00 (m, 1H), 6.89-6.84 (dd, 2H), 5.34 (t, 2H), 5.26 (-O-CH2-), 4.73-4.72(*-CH2-N*), 3.67, 2.62, 2.24, 1.54(*t, 4H, -NH2*), 0.88 *(t, -CH3*). ^{13}C NMR (101 MHz, $CDCl_3$) δ 143.20, 142.07, 131.76, 130.32, 129.36, 128.82, 123.57, 120.99, 118.97, 112.05, 59.86, 31.27. ESIMS, m/z 441.2 [M-C_9H_{18}]$^+$.

2-Azidoethylamine-2-Bromoethylamine hydrobromide (500 mg, 2.44 mmol) and sodium azide (475.9 mg, 7.32 mmol) were dissolved in H_2O (2 mL); the solution was heated to 75°C, stirred for 21 h, and cooled to 0°C. To this mixture were added KOH (800 mg) and Et_2O (2 mL), and the solution was extracted with Et_2O (2×10 mL) and concentrated in vacuum. The resultant residue was purified by column chromatography with $CHCl_3$-MeOH (20:1) to afford 2-Azidoethylamine (171 mg, 82% yield). Colorless liquid (from ethyl ether). ^{1}H NMR (300 MHz, $CDCl_3$) δ 2.79 (s, 2H), 2.04 (s, 2H), 1.58 (m, 2H).13C NMR ($CDCl_3$, 101 MHz) δ 56.14 (CH2N3), 41.03 (CH2NH2); ESIMS m/z 123.9 [M+H]$^+$.

Compound **8**: yield 28%; Yellow liquid; R_f = 0.90; ^{1}H NMR (400 MHz, $CDCl_3$) δ 7.53 (d, J = 8.5 Hz, 1H), 7.52 – 7.05 (m, 3H), 6.16 (m, 1H), 5.90 (m, 1H), 5.72 (m, 1H), 5.69-5.64(m, 3H), 3.67 (s, 1H). ^{13}C NMR (101 MHz, $CDCl_3$) δ 203.94, 202.90, 193.34, 183.15, 154.44, 154.00, 141.35, 128.62, 128.14, 128.09, 127.56, 124.51, 120.22, 119.18, 118.24, 111.08, 110.11. ESIMS m/z 367.1 [M-$C_6H_5BO_2$-CH_3]$^+$.

Compound **9**: yield 25%; Yellow liquid; R_f = 0.85; ^{1}H NMR (400 MHz, CDCl3) δ 7.72 (t, J = 8.6 Hz, 1H), 7.53 (d, J = 5.13Hz, 1H), 7.36 (s, 1H), 7.07 (s, 1H), 6.70 (m, 8H), 6.10 (m, 1H), 5.84 (m, 1H), 5.60 (m, 1H), 5.40-5.34(m, 3H), 3.67 (s, 2H), 2.40. ^{13}C NMR (101 MHz, $CDCl_3$) δ 205.10, 195.12, 184.93, 156.22, 155.78, 143.14, 130.40, 129.93, 129.88, 129.34, 126.30, 122.00, 120.96, 120.02, 112.86, 111.90. ESIMS m/z 405.2 [M+H]$^+$.

Syntheses of Compound **10**. Triene uruhsiol (0.5 mmol) was dissolved in dry MeCN (3 mL), SiO_2/$NaHSO_4$ (1 mmol) and ethyl acetoacetate (100 μL, 1.25 mmol) were added. The mixture was stirred at r.t for 4 h and centrifuged with H_2O (50 mL). The solution was extracted with EA and

evaporated to remove EA. The organic layer was washed with saturated $NaHCO_3$ and brine, dried over $MgSO_4$, filtered, and finally concentrated. The residue was purified by column chromatography with PE-EA (100:10) to produce compound **10**.

Compound **10:** (8-hydroxy-4-methyl-7-((8Z,11E,13Z)-penta deca-8,11,13-trien-1-yl)-2H -chromen-2-one) Yellow liquid. Yield: 38 mg, 20%. ^{1}H NMR (400 MHz, $CDCl_3$) δ 6.75 (d, 5H), 6.68 (d, J = 10.0 Hz, 1H) 6.22 (s, 1H), 6.02-5.88 (5H),2.94 (m,2H), 2.68(s,2H), 2.38(t,4H), 2.11((d, J=67.7Hz, 2H)), 1.94(m, 3H), 1.50(m,3H),1.36(m,3H),1.27(m,3H). ^{13}C NMR (101 MHz, $CDCl_3$) δ 167.46, 165.57, 164.44, 161.42, 155.03, 143.34,142.18, 124.42, 122.44, 120.20, 119.95, 112.86, 112.07, 110.26, 106.90, 50.10, 30.13, 28.45, 28.28, 27.07, 25.15, 21.19, 19.51, 15.53, 14.11, 14.04. ESIMS m/z 374.3 [M-$2CH_3$+Na]$^+$.

Compound **11**: yield 23%; Yellow liquid; R_f = 0.87; ^{1}H NMR (400 MHz, $CDCl_3$) δ 7.79 (m, 1H), 7.52 (d, J = 7.85Hz, 1H), 6.71 (s, 7H), 5.87 (m, 1H), 5.63 (m, 1H), 5.16 (m, 1H), 5.05-4.98(m, 3H), 3.67 (s, 3H). ^{13}C NMR (101 MHz, $CDCl_3$) δ 32.23, 31.43, 29.74, 29.27, 27.46, 22.67, 14.10, -0.02. ESIMS m/z 421.4 [M-C_5H_8-F+2H]$^+$.

Compound **12**: yield 29%; Yellow liquid; R_f = 0.85; ^{1}H NMR (400 MHz, $CDCl_3$) δ 7.53 (m, 1H), 7.38 (d, J = 7.15Hz, 1H), 6.98 (s, 7H), 5.81 (m, 1H), 5.64 (m, 1H), 5.15 (m, 1H), 5.07-5.01(m, 3H), 3.66 (s, 3H). ^{13}C NMR (101 MHz, $CDCl_3$) δ 207.12, 206.08, 196.52, 186.33, 157.62, 157.18, 144.53, 131.80, 131.32, 131.27, 130.73, 127.69, 123.40, 122.35, 121.41, 114.26, 113.29, 78.71. ESIMS m/z 501.2 [M+2H]$^+$.

Compound **13**: 3-((8Z,11Z)-pentadeca-8,11-dien-1-yl) benzene-1,2-diol. Viscous liquid (purity 93.01%) ^{1}H NMR (400 MHz, $CDCl_3$) δ 6.71 (s, 3H), 5.37-5.36 (4H), 5.34, 2.78 (t, 1H), 2.60 (t, 1H), 2.05, 2.04 (dd, J = 7.2 Hz, 3H), 2.03, 1.59, 1.43 – 1.30 (m, 10H), 0.91 (t, 1H). ^{13}C NMR (101 MHz, $CDCl_3$) δ 142.99, 141.84, 130.13, 129.93, 129.32, 128.16, 128.00, 122.12, 120.12, 112.89, 29.75, 29.74, 29.65, 29.48, 29.40, 29.30, 29.24, 27.22, 25.65, 22.80, 13.79. ESIMS m/z 316.72 [M]$^+$.

Compound **14**: 3-((8Z,11Z)-pentadeca-8,11,14-trien-1-yl) benzene-1,2-diol. Viscous liquid (purity 97.98%) ^{1}H NMR (400 MHz, $CDCl_3$) δ 6.71 (d, J = 4.0 Hz, 3H), 5.84, 5.80, 5.42, 5.35, 5.12, 4.99, 2.83, 2.81 (m, J = 16.0 Hz, 2H), 2.79, 2.60 (s, 1H), 2.05, 2.04 (d, J = 6.6 Hz, 1H), 2.03, 1.61, 1.36–1.30 (m, 10H). ^{13}C NMR (101 MHz, $CDCl_3$) δ 142.99, 141.85, 136.85, 130.41, 129.31, 127.59, 126.83, 122.13, 120.13, 114.70, 112.90, 31.53, 29.76, 29.74, 29.62, 29.48, 29.40, 29.23, 27.23, 25.59. ESIMS m/z 314.26 [M]$^+$.

Syntheses of Compound **15-20**. Maleic anhydride was added to the dried pressure tube and stirred evenly in an oil bath. Refined lacquer(C15 triene urushiol purity > 95%) and maleic anhydride molar ratio was 0.5:1; Temperature kept at 160℃; refined C15 triene urushiol and maleic anhydride were mixed in the high temperature environment and stirred at 160°C for 6 h, stopped the reaction and cooled to room temperature to obtain the target organic synthesis product.

Compound **15**: (Z)-4-(10-(2,3-dihydroxyphenyl) dec-2-en-1-yl)-3a,7a-difluoro-7-methyl-3a,4, 7,7a -tetrahydroisobenzofuran-1,3-dione. Brown solid (purity 95%). ^{1}H NMR (400 MHz, $CDCl_3$) δ 6.99, 6.42, 6.40, 6.39, 5.26, 5.22, 5.12, 5.10, 3.44, 3.34, 2.33, 2.20, 1.72, 0.79, 0.00. ^{13}C NMR (101 MHz, $CDCl_3$) δ 172.52, 170.41, 141.27, 140.10, 130.50, 130.41, 129.96, 129.86, 124.46, 119.74, 117.87, 110.96. ESIMS m/z 503.2 [M+C_4H_7]$^+$. Anal. Calcd for $C_{25}H_{30}F_2O_5$: C66.95, H6.74, F8.47, O17.84. Found C66.83, H6.71, F8.36, O18.10. (Compounds **7-12** ware analyzed by CHNS pattern)

Compound **16**: (Z)-3a,7a-dichloro-4-(10-(2,3-dihydroxyphe nyl) dec-2-en-1-yl)-7-methyl-3a,4, 7,7a-tetrahydroisobenzofuran-1,3-dione. Yield 41%; Brown solid. Brown solid (purity 95%). ^{1}H NMR (400 MHz, $CDCl_3$) δ 7.17, 6.61, 6.59, 6.58, 5.45, 5.41, 5.30, 5.28, 3.63, 3.52, 1.91, 0.97. ^{13}C NMR (101 MHz, $CDCl_3$) δ 171.26, 169.15, 140.01, 138.84, 129.24, 129.15, 128.70, 128.60, 123.20, 118.48, 116.61, 109.70. ESIMS m/z 503.2 [M+$2CH_3$]$^+$. Anal. Calcd for $C_{25}H_{30}Cl_2O_5$: C62.37, H6.28, Cl14.73, O16.62. Found C62.33, H6.19, Cl4.39, O17.09.

Compound **17**: (Z)-3a,7a-dibromo-4-(10-(2,3-dihydroxyphen yl) dec-2-en-1-yl)-7-methyl- 3a,4, 7,7a-tetrahydroisobenzofuran-1,3-dione. Yield 42%; Brown solid. Brown solid (purity 95%). ^{1}H NMR (400 MHz, $CDCl_3$) δ 7.06, 6.49, 6.47, 6.46, 5.33, 5.29, 5.19, 5.17, 3.51, 3.41, 2.40, 2.27, 1.79, 0.85, 0.00. ^{13}C NMR (101 MHz, $CDCl_3$) δ 172.83, 170.71, 141.58, 140.41, 130.81, 130.72, 130.27, 130.16, 124.76, 120.05, 118.17, 111.27. ESIMS m/z 526.4 [M-CH_3CHNH_2]$^+$. Anal. Calcd for $C_{25}H_{30}Br_2O_5$: C52.65, H5.30, Br28.02, O14.03. Found C51.39, H5.26, Br28.00, O15.35.

Compound **18**: (Z)-4-(10-(2,3-dihydroxyphenyl) dec-2-en-1-yl)-7-methyl-3a,4,7,7a-tetrahydro -1H -isoindole-1,3(2H)-dione. Yield 36%; Brown solid. ^{1}H NMR (600 MHz, $CDCl_3$) δ 7.41, 7.06, 6.66, 6.65, 6.64, 6.35, 5.94, 5.63, 5.36, 3.71, 3.62, 2.25, 2.19, 0.88. ^{13}C NMR (101 MHz, CDCl3) δ 143.43, 142.38, 133.69, 125.81, 124.36, 121.71, 119.72, 112.74, 71.65, 58.45, 37.40, 36.90, 18.34. Brown solid (purity 95%). ESIMS m/z 429.1 $[M+CH_3+H]^+$. Anal. Calcd for $C_{25}H_{33}NO_4$: C72.96, H8.08, N3.40, O15.55. Found C72.85, H7.65, N3.29, O16.21.

Compound **19**: (Z)-3a,7a-dichloro-4-(10-(2,3-dihydroxyphen yl) dec-2-en-1-yl)-7-methyl-3a,4, 7,7a -tetrahydro-1H-isoindole-1,3(2H)-dione. Yield 50%; Brown solid. ^{1}H NMR (600 MHz, $CDCl_3$) δ 7.31, 6.96, 6.56, 6.55, 6.54, 6.25, 5.84, 5.54, 5.26, 3.62, 3.52, 2.16, 2.09, 0.78. ^{13}C NMR (101 MHz, $CDCl_3$) δ 144.14, 143.10, 134.41, 126.53, 125.08, 122.43, 120.44, 113.46, 72.36, 59.17, 38.11, 37.62, 19.06. Brown solid (purity 95%). ESIMS m/z 503.1 $[M+2CH]^+$. Anal. Calcd for $C_{25}H_{31}Cl_2NO_4$: C62.50, H6.50, Cl: 14.76, N2.92, O13.32. Found C62.30, H6.36, Cl: 14.71, N2.98, O13.65.

Compound **20**: (Z)-3a,7a-dibromo-4-(10-(2,3-dihydroxyphen yl) dec-2-en-1-yl)-7-methyl -3a,4, 7,7a-tetrahydro-1H-isoindole-1,3(2H)-dione. Yield 54%; Brown solid (purity 95%). ^{1}H NMR (600 MHz, $CDCl_3$) δ 9.12, 6.67, 6.66, 6.33, 5.96, 5.64, 5.38, 2.59. ^{13}C NMR (151 MHz, CDCl3) δ 140.84, 139.79, 131.10, 123.22, 121.78, 119.12, 117.13, 110.15, 69.06, 55.86, 34.81, 34.31, 15.75. ESIMS m/z 503.1 $[M+2CH_3\text{-}C_6H_5O]^+$. Anal. Calcd for $C_{25}H_{31}Br_2NO_4$: C52.74, H5.49, Br:28.07, N2.46, O11.24. Found C52.66, H5.40, Br: 28.28, N2.59, O11.07.

Synthesis of Compounds **21** and **22**. The method was as the above synthesis of compounds **3** and **6**.

Compound **21** (Z)-4-methyl-7-(pentadec-8-en-1-yl)-8-(prop-2-yn-1-yloxy)-2H-chrome-2- one Yellow liquid. Yield: 21 mg, 64%. ^{1}H NMR (400 MHz, $CDCl_3$) δ6.70(s, 1H), 6.31(dd, J = 17.1 Hz, J = 21.9 Hz,1H), 6.01(dd, J = 10.9 Hz, J = 18.1 Hz,1H), 3.5(s,2H), 2.60(t, J=7.4Hz,2H), 2.40(m,2H). ^{13}C NMR (101 MHz, $CDCl_3$) δ 168.85, 140.61, 139.50, 129.78, 128.55, 127.42, 126.85, 124.28, 123.01, 121.81, 119.44, 117.45, 110.30. ESIMS m/z 406.6 $[M–H_2O]^+$.

Compound **22** (4-methyl-7-((8Z,11E,13Z)-pentadeca-8,11, 13-trien-1-yl)-8-(prop-2 –yn -1-yloxy)-2H-chro men-2-one) Yellow liquid. Yield: 301 mg, 73%. ^{1}H NMR (400 MHz, $CDCl_3$) δ6.70(s, 1H), 6.03(dd, J = 21.1 Hz, J = 27.9 Hz,2H), 5.44–5.17(6H), 4.12(m,2H), 2.53(t J=7.4Hz,2H), 2.40(s,3H), 2.05(d,3H), 1.73(d, J=4.4Hz,2H), 1.60(m,2H), 1.33 (m,2H), 1.26(m,2H), 0.88(m,2H). ^{13}C NMR (101 MHz, $CDCl_3$) δ 171.43, 143.17, 142.05, 132.34, 131.04, 130.02, 129.92, 129.40, 129.37, 126.84, 124.36, 121.99, 120.01, 112.85, 61.77, 60.52, 50.89,31.98, 31.81, 30.64, 29.79, 29.52, 27.23, 22.68, 21.08, 14.20, 13.32. ESIMS m/z 402.1 $[M–H_2O]^+$.

^{1}H/^{13}C NMR Compound 1 Triene Urushiol

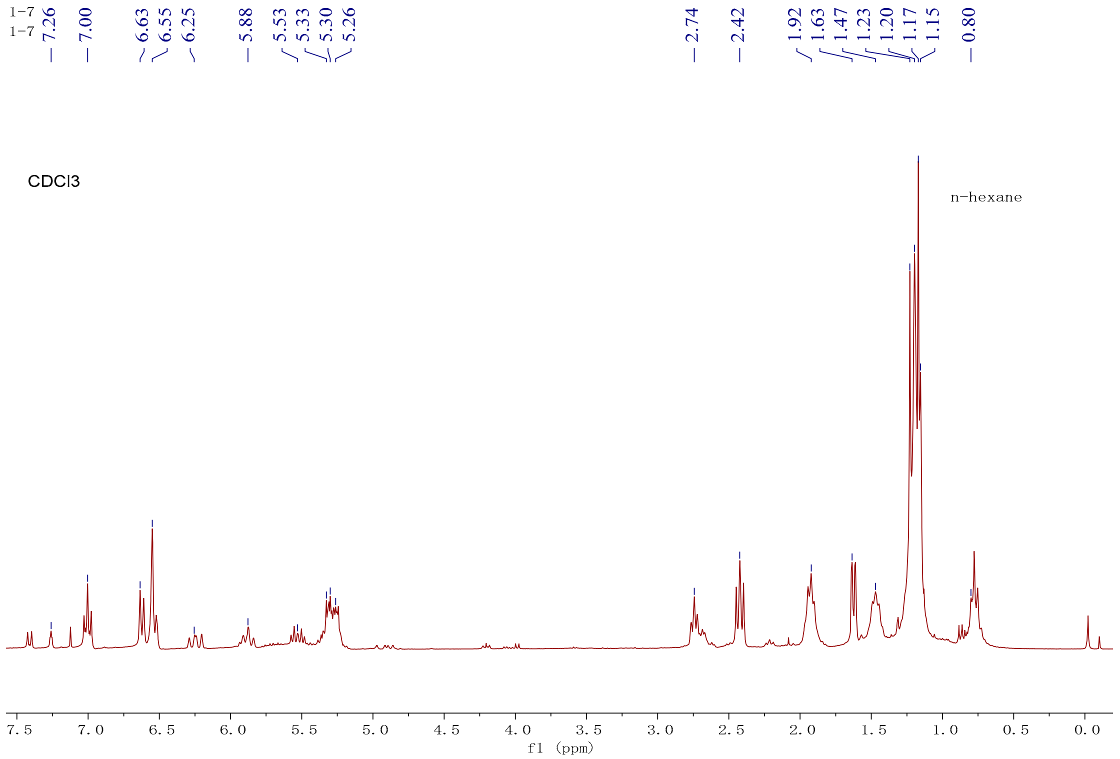

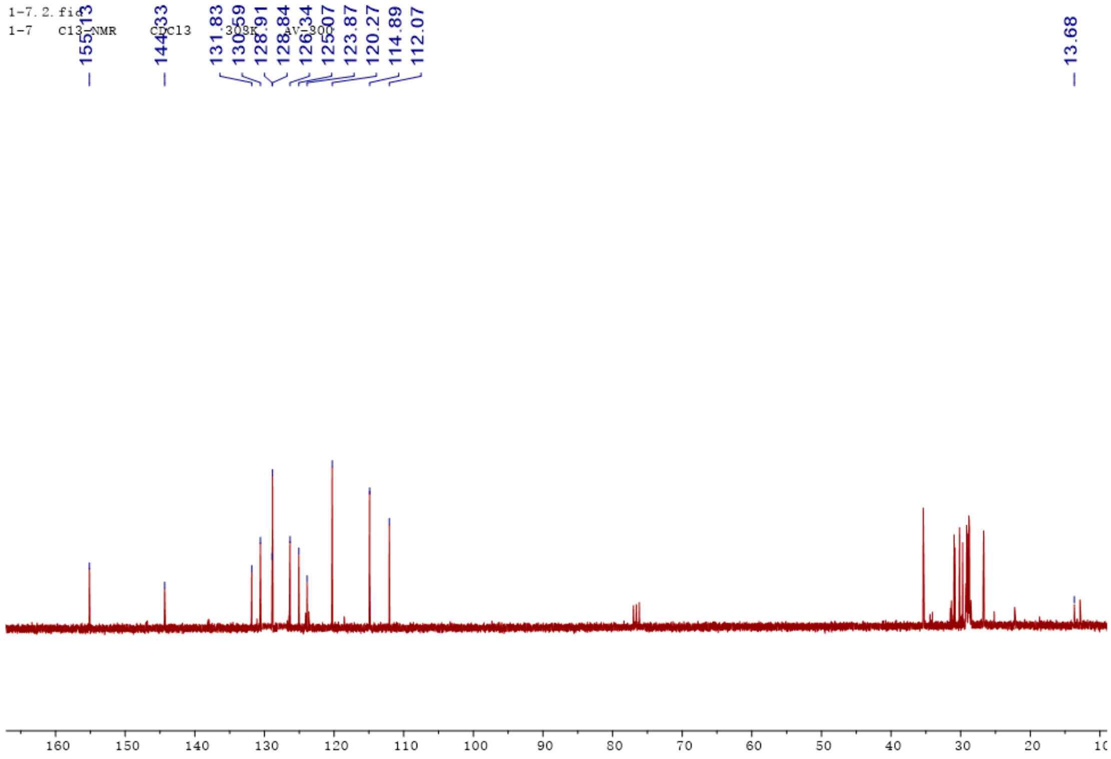

^{1}H/^{13}C NMR Compound 2 Mono-olefins Uruhsiol

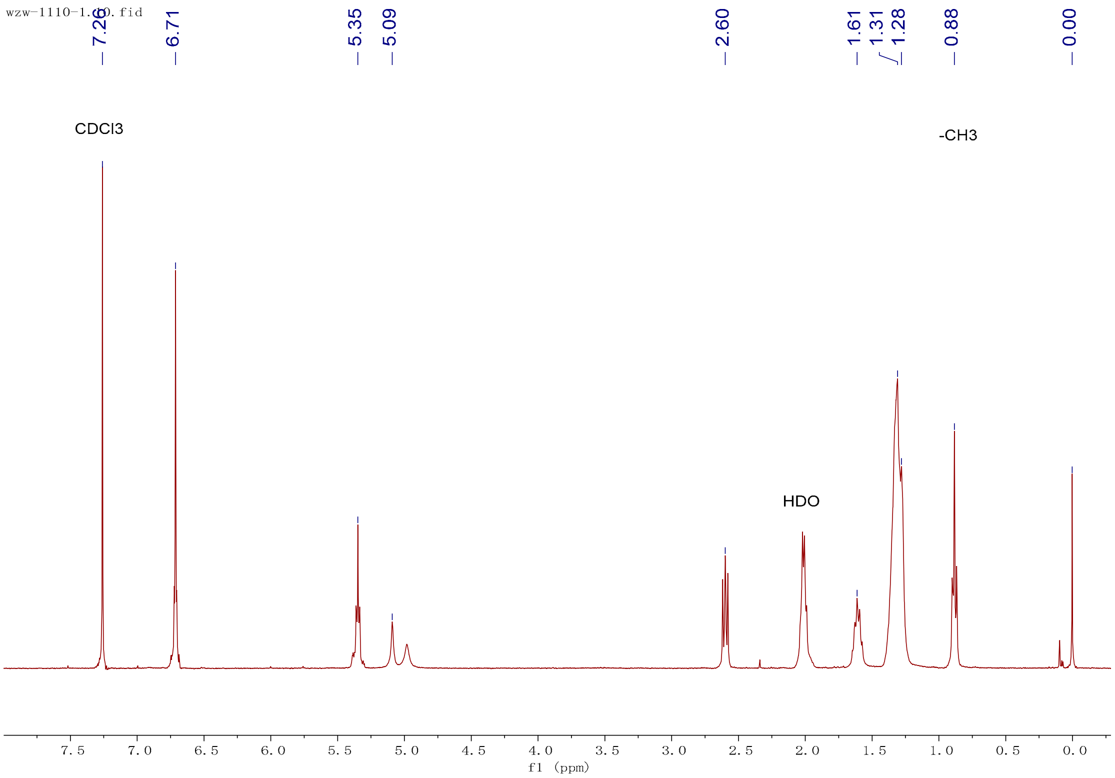

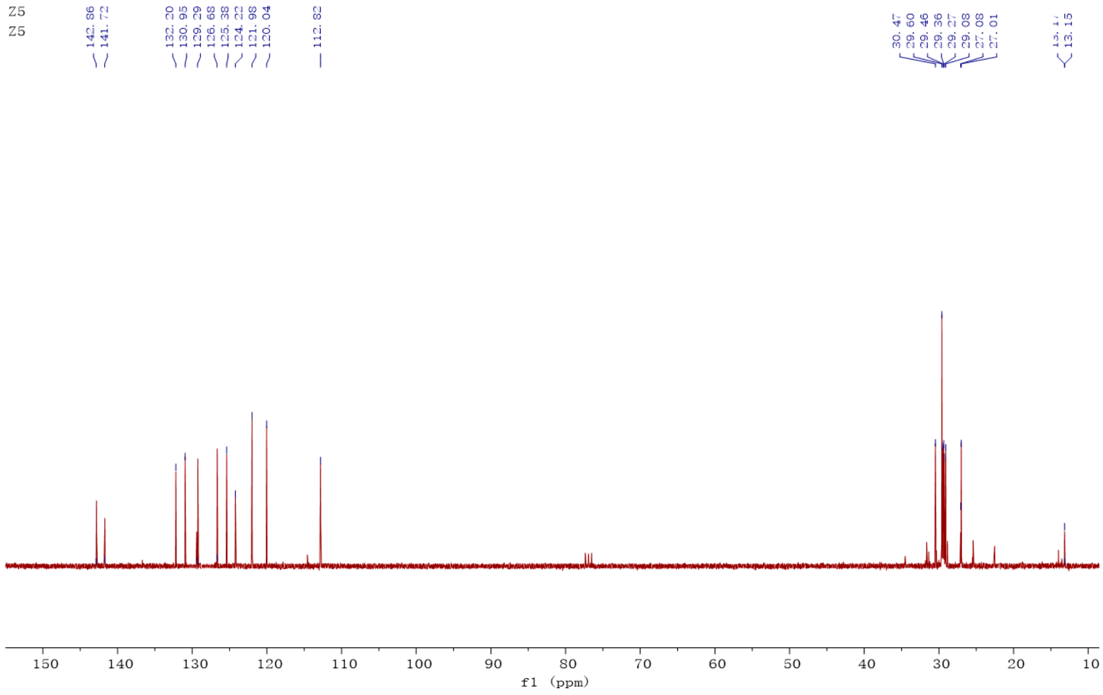

$^{1}H/^{13}C$ NMR Compound 3 ((Z)-1-(hexadec-9-en-1-yl)-2,3-bis(prop-2-yn-1-yloxy)benzene)

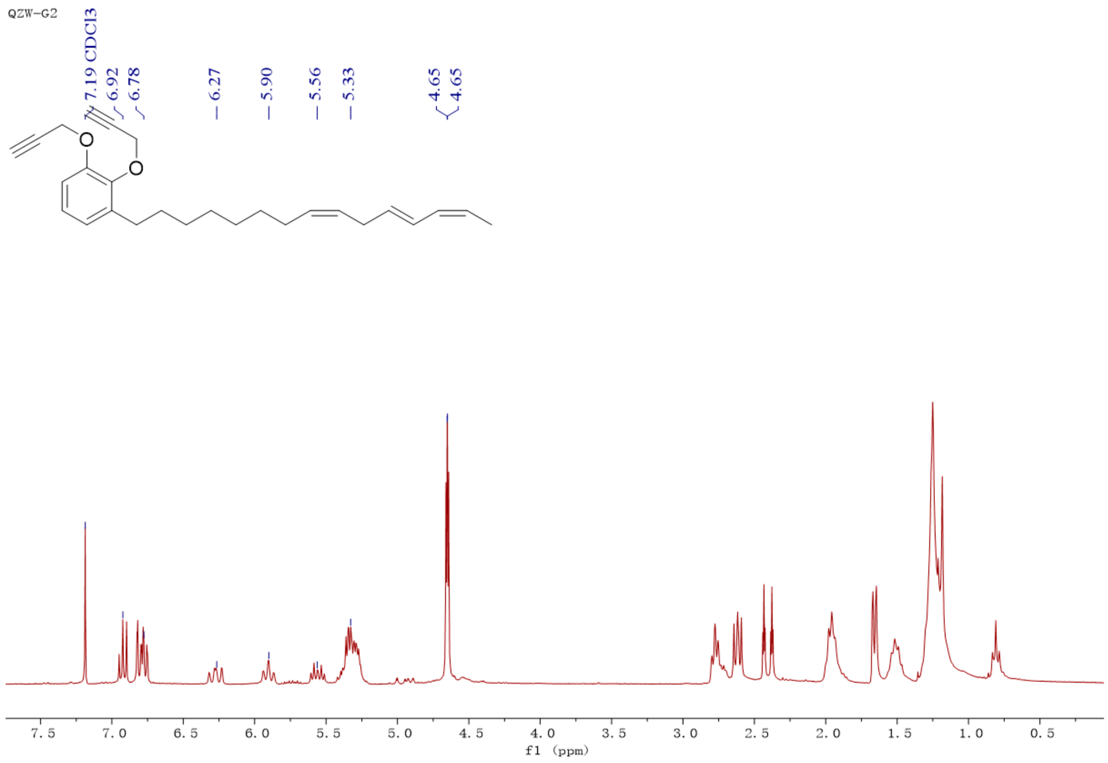

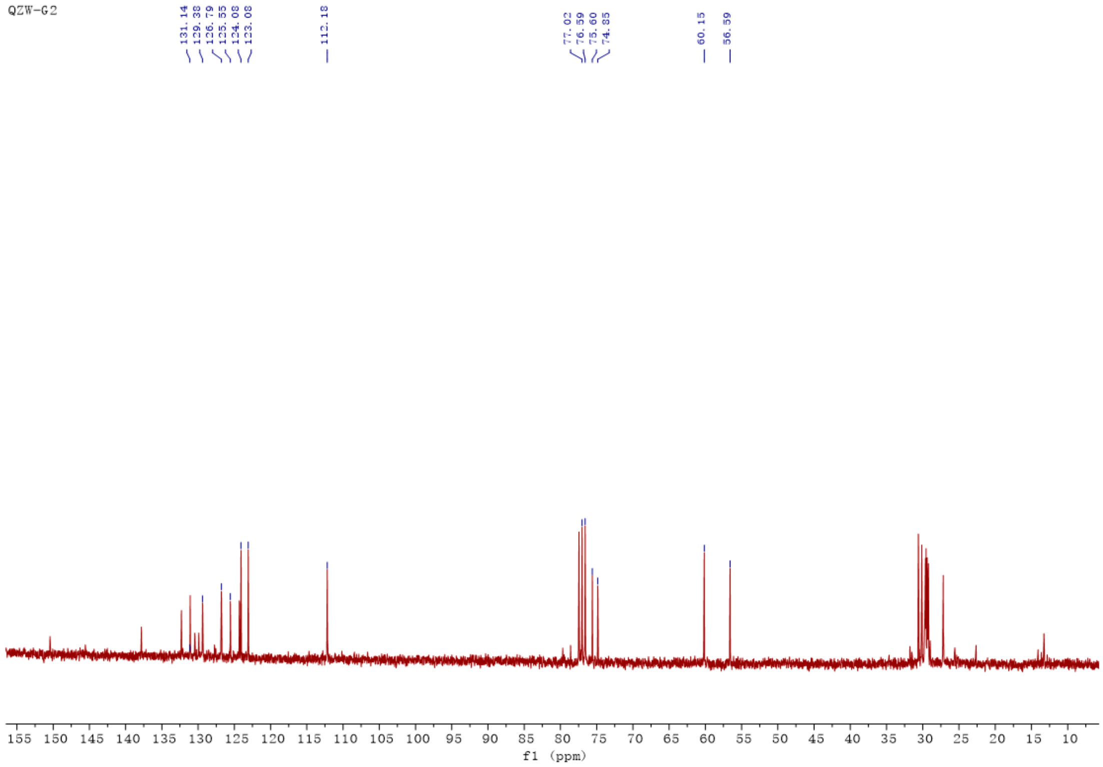

$^1H/^{13}C$ NMR Compound 4

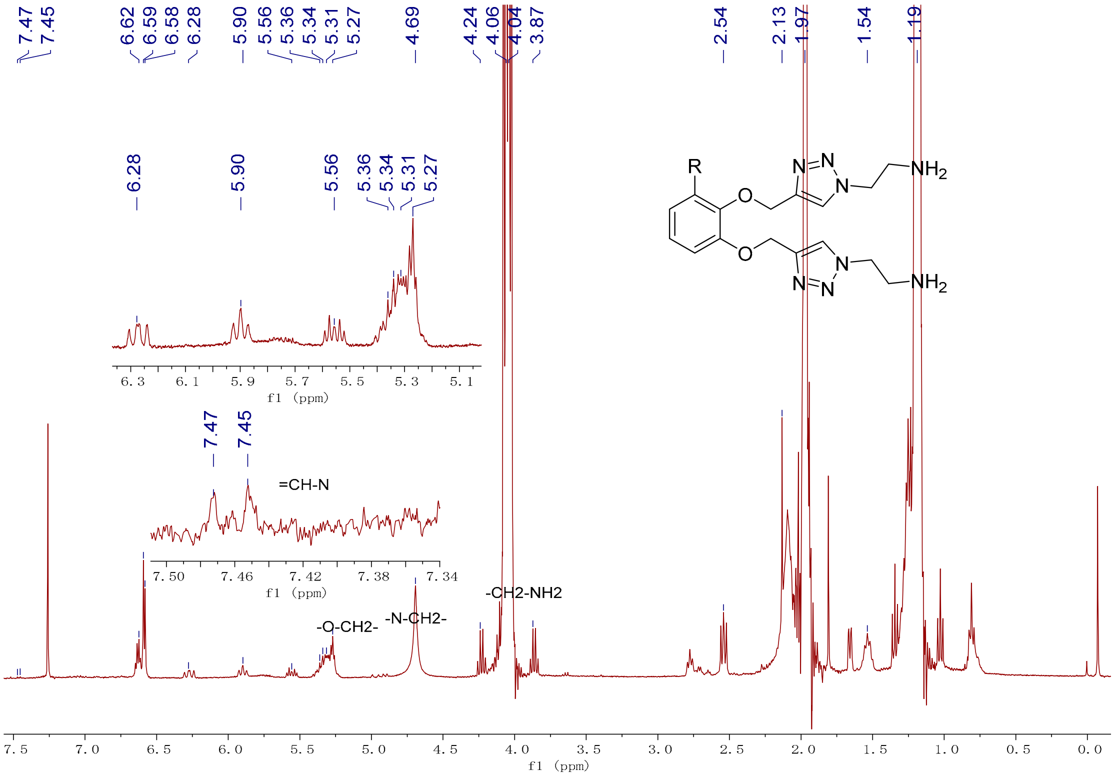

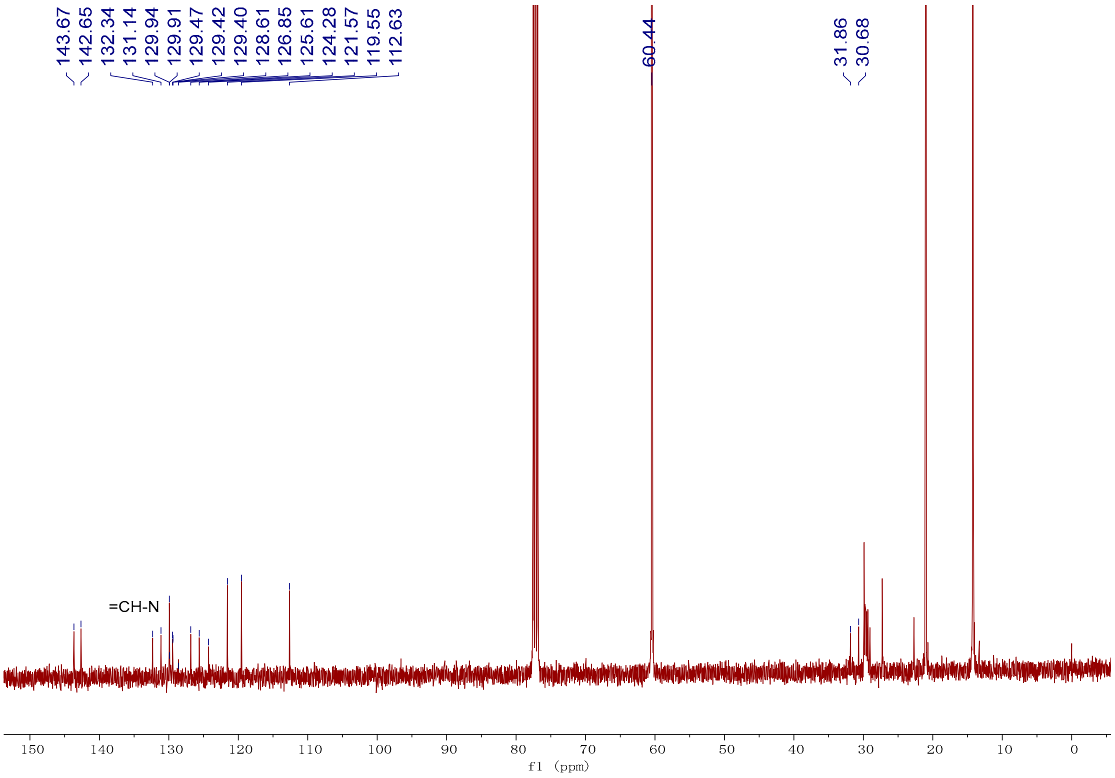

$^{1}H/^{13}C$ NMR Compound 5 Levopimaric Acid

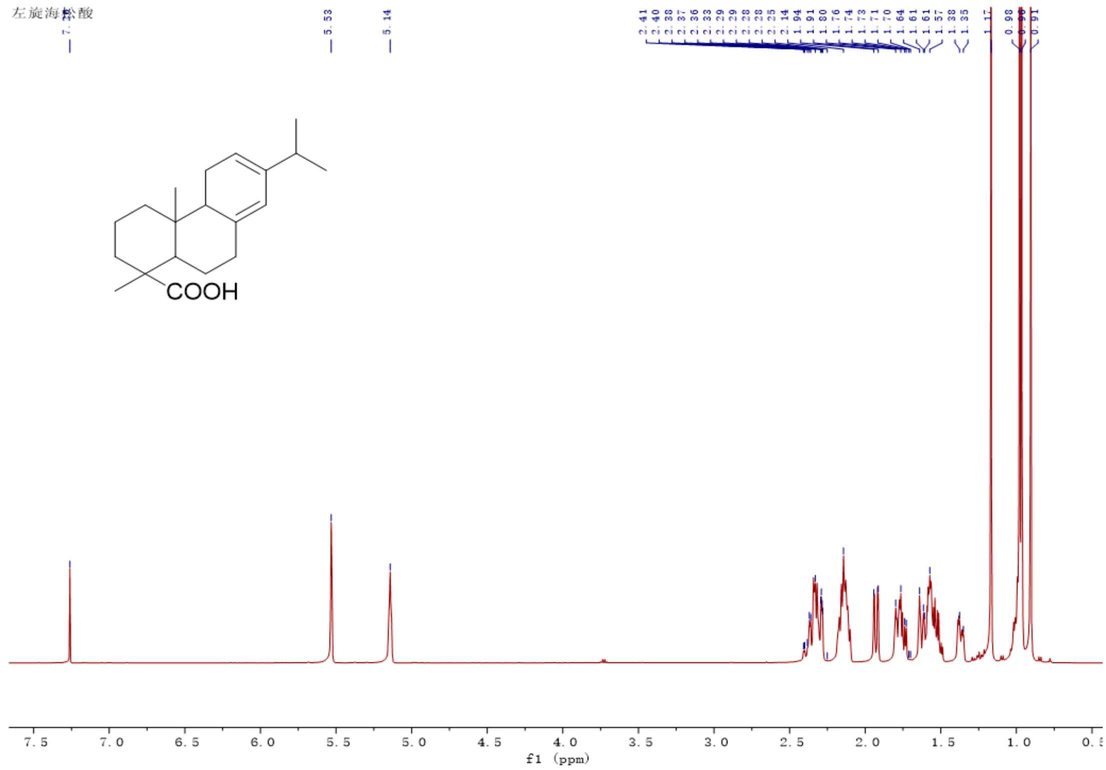

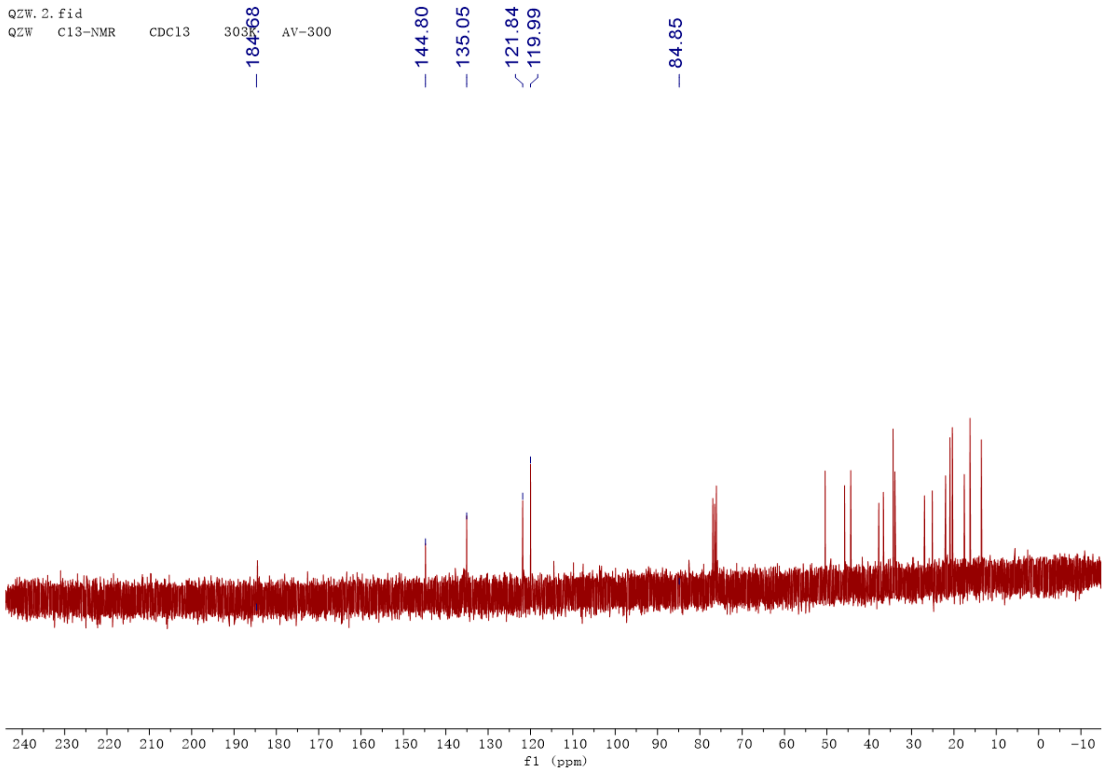

$^{1}H/^{13}C$ NMR Compound 6

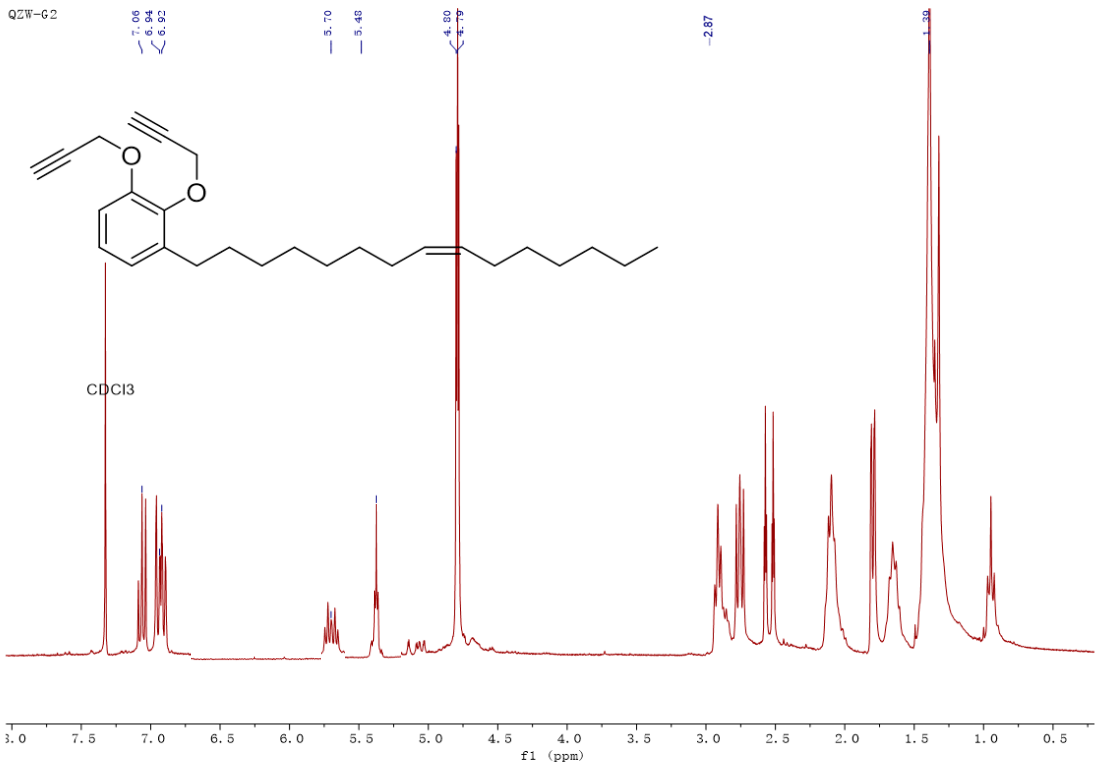

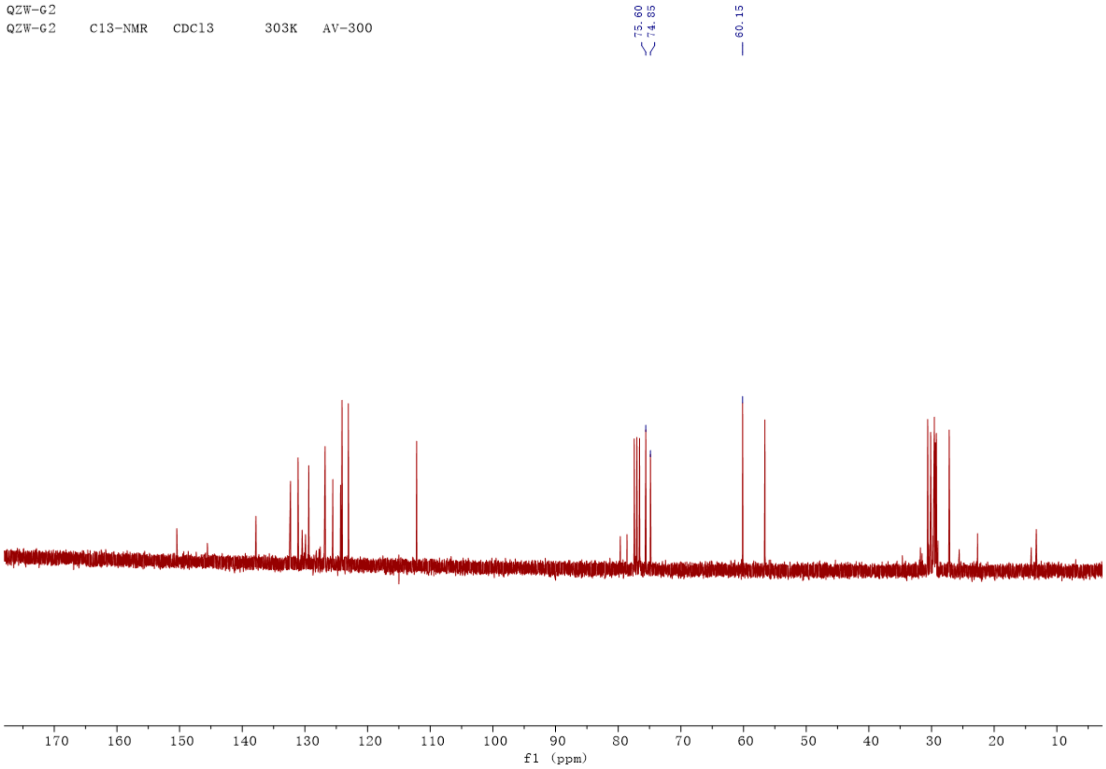

^{1}H/^{13}C NMR Compound 7

(1-((8Z,11E,13Z)-pentadeca-8,11,13-trien-1-yl)-2,3-bis(prop-2-yn-1-yloxy) benzene).

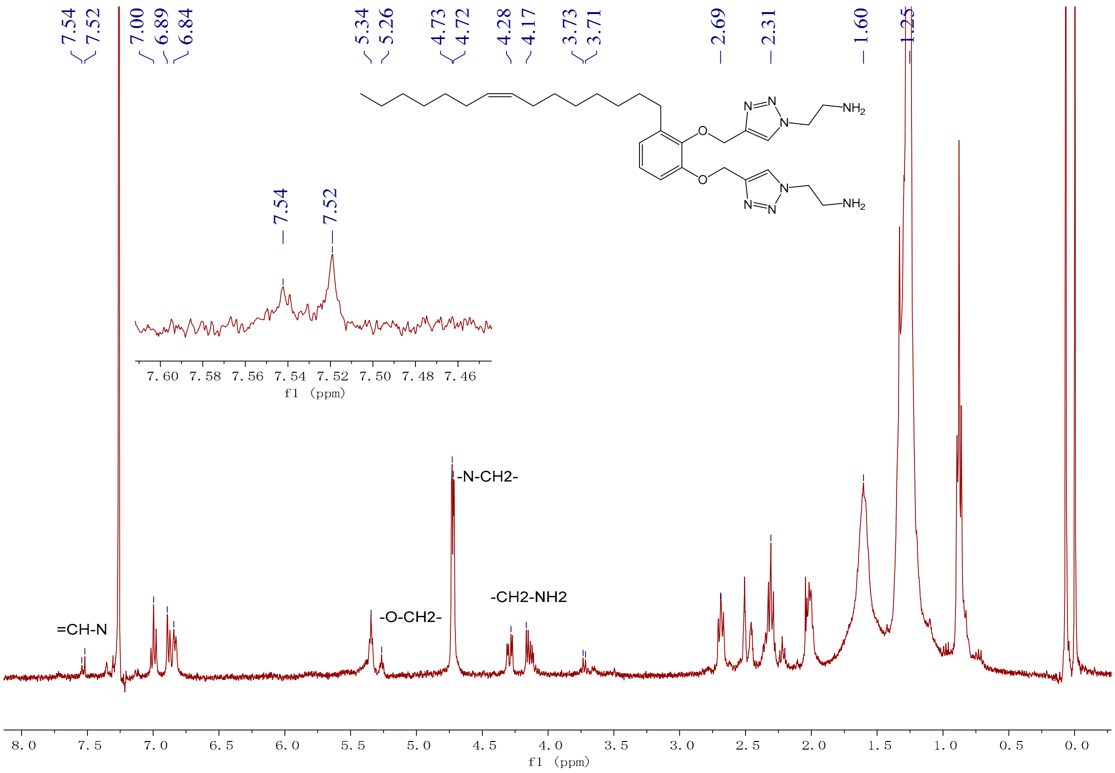

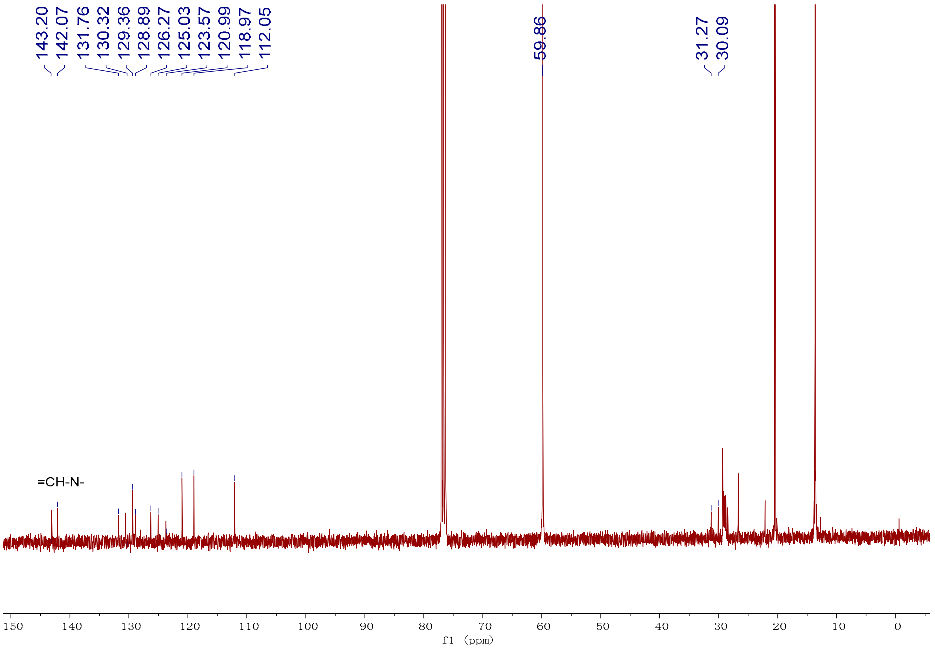

^{1}H NMR Compound 8

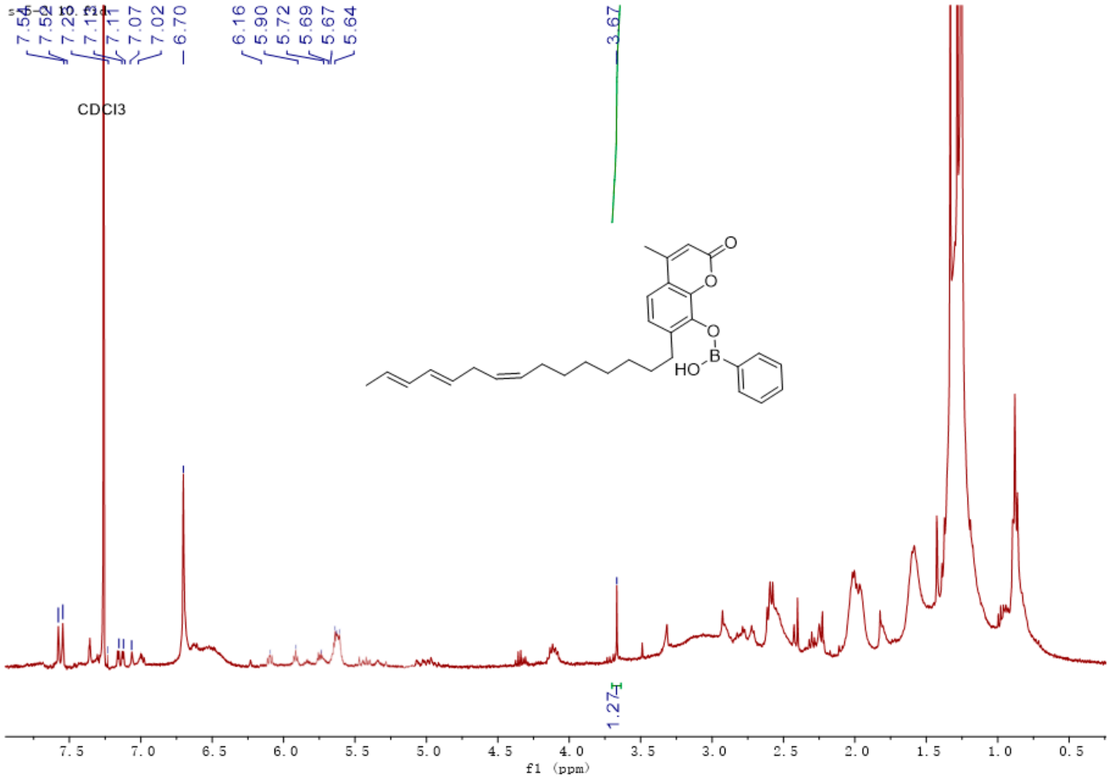

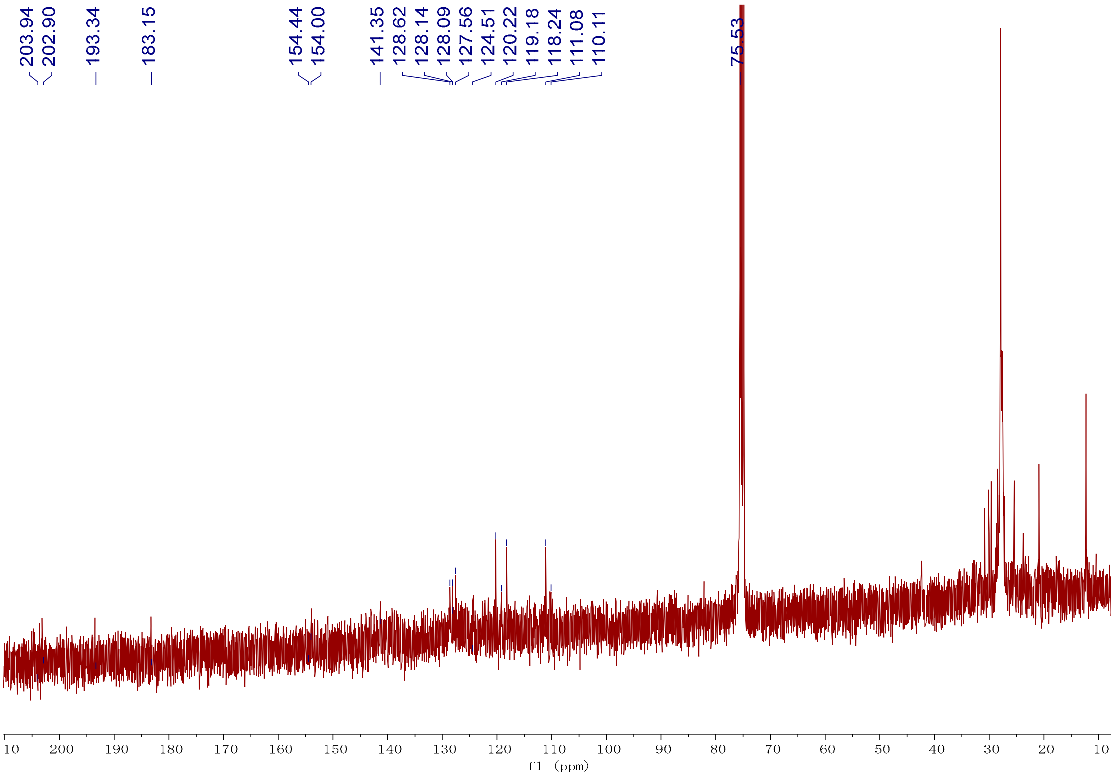

^{1}H NMR Compound 9

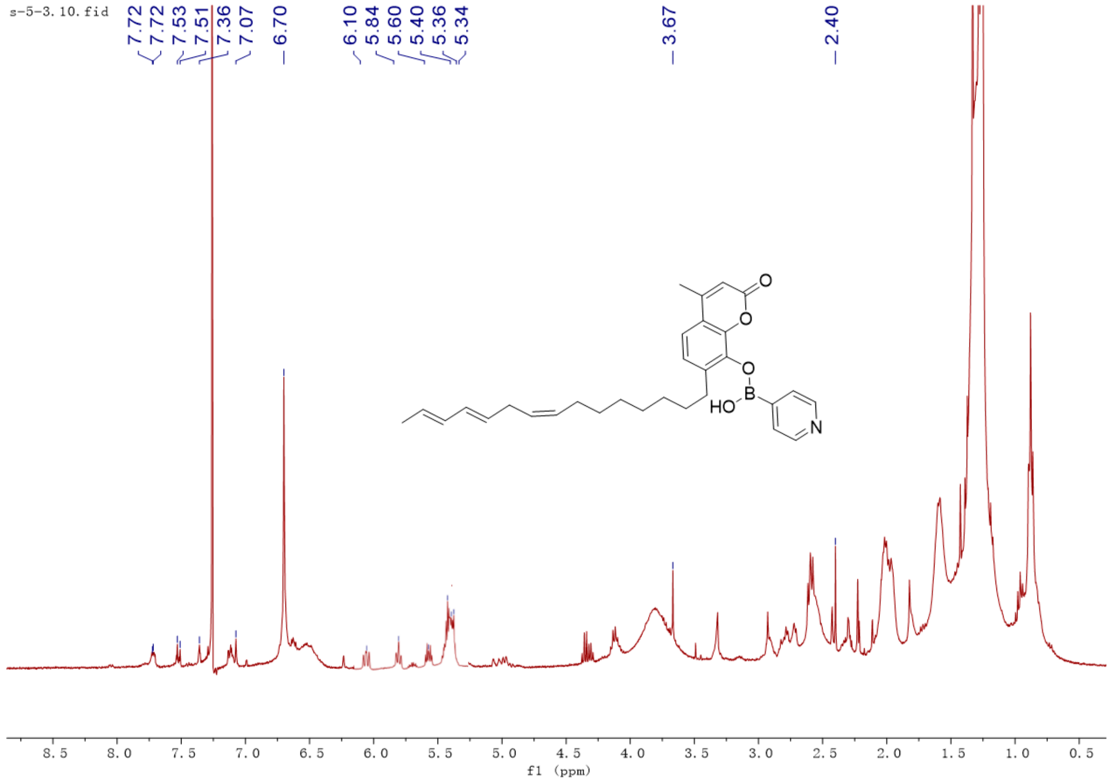

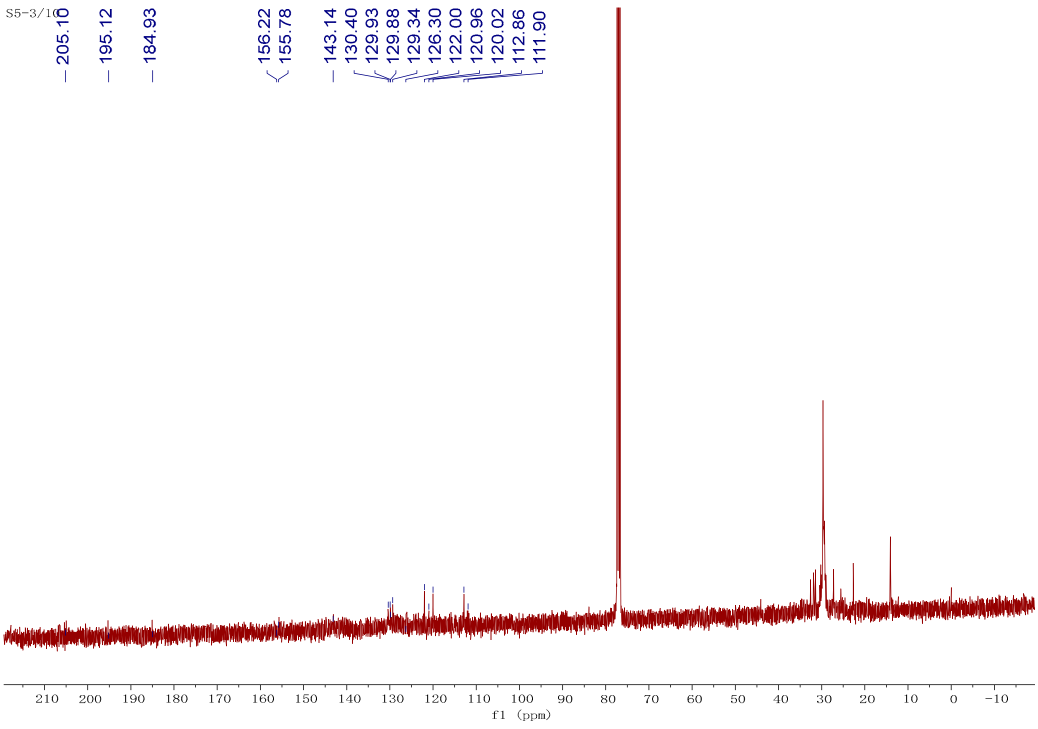

^{1}H/^{13}C NMR Compound 10.

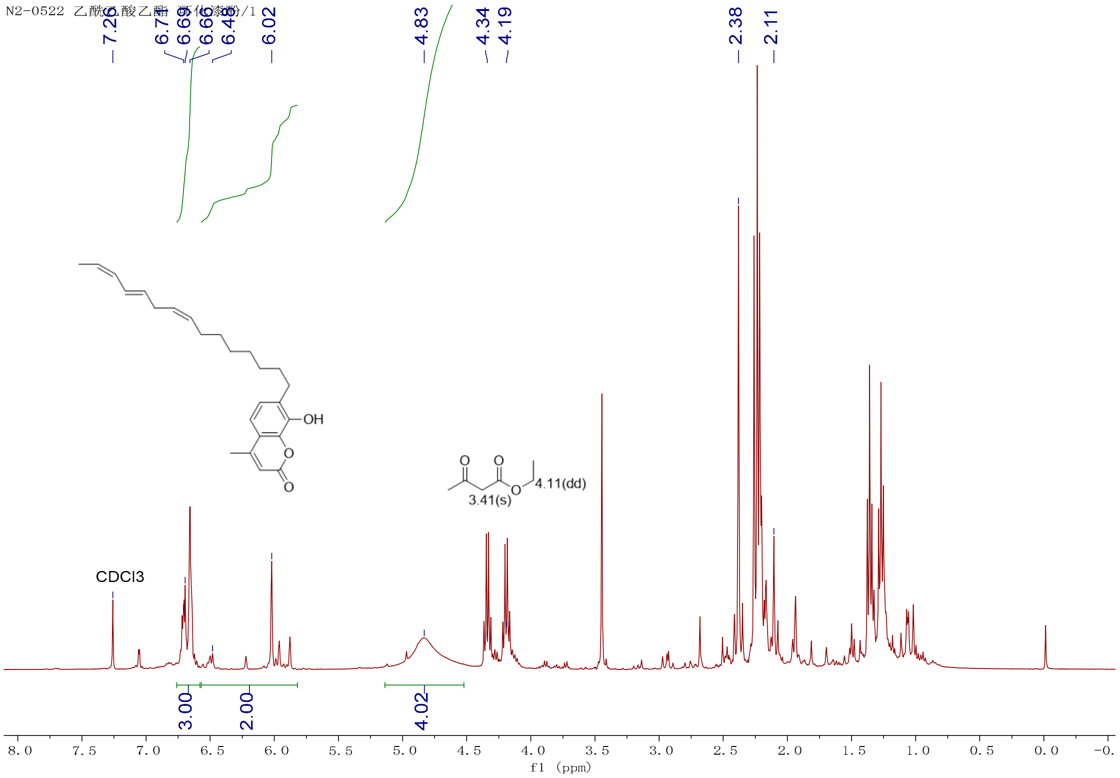

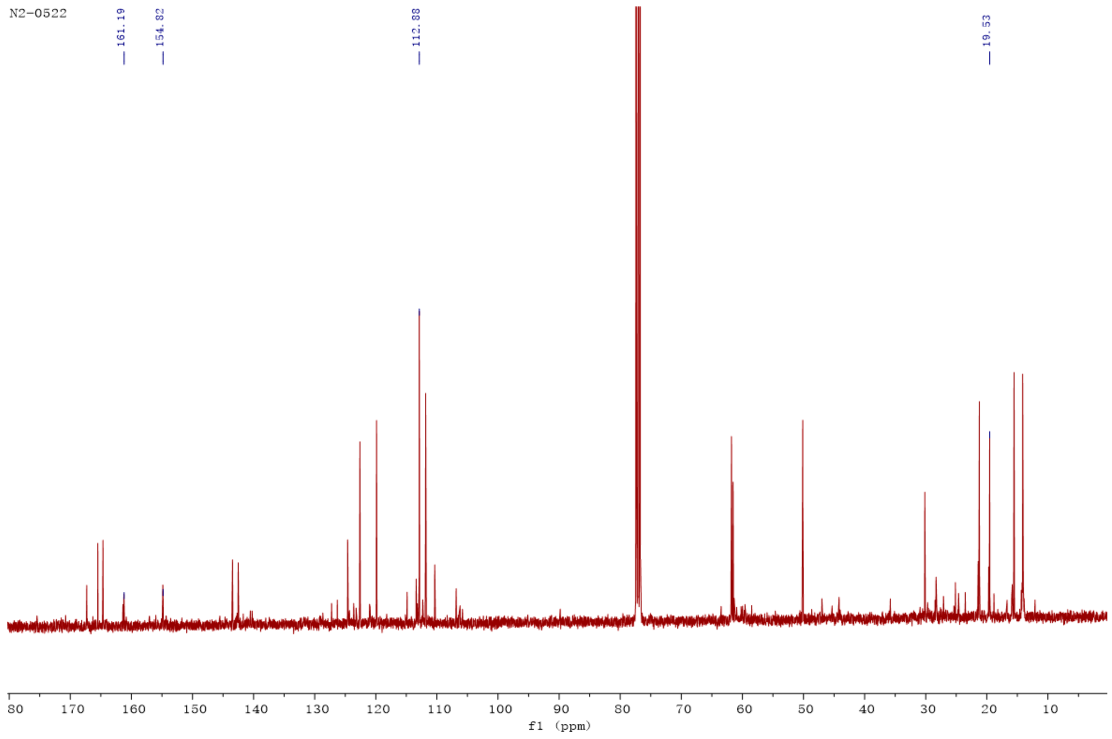

^{1}H NMR Compound 11

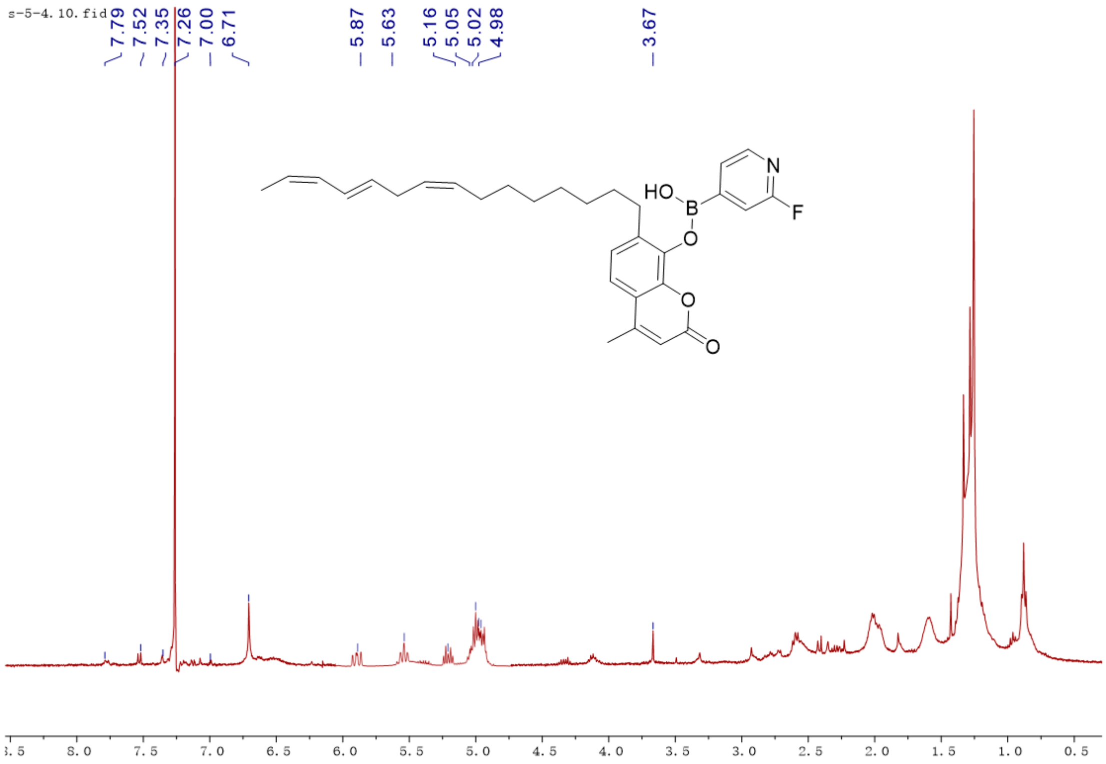

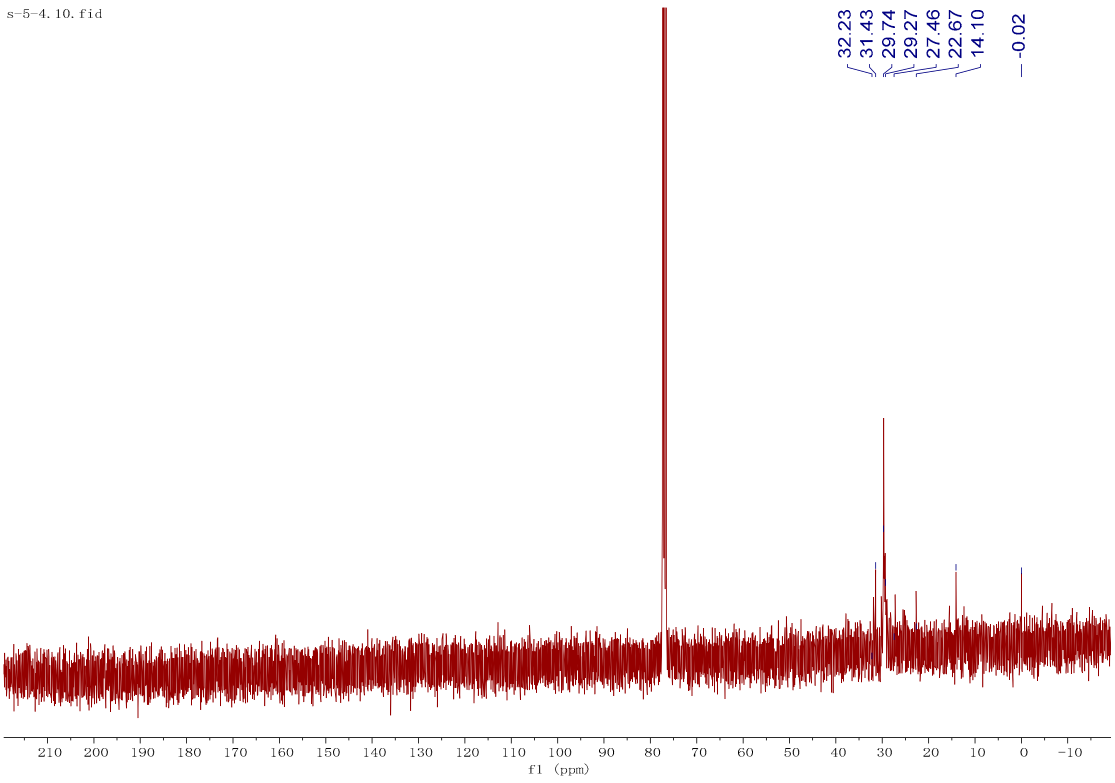

^{1}H NMR Compound 12

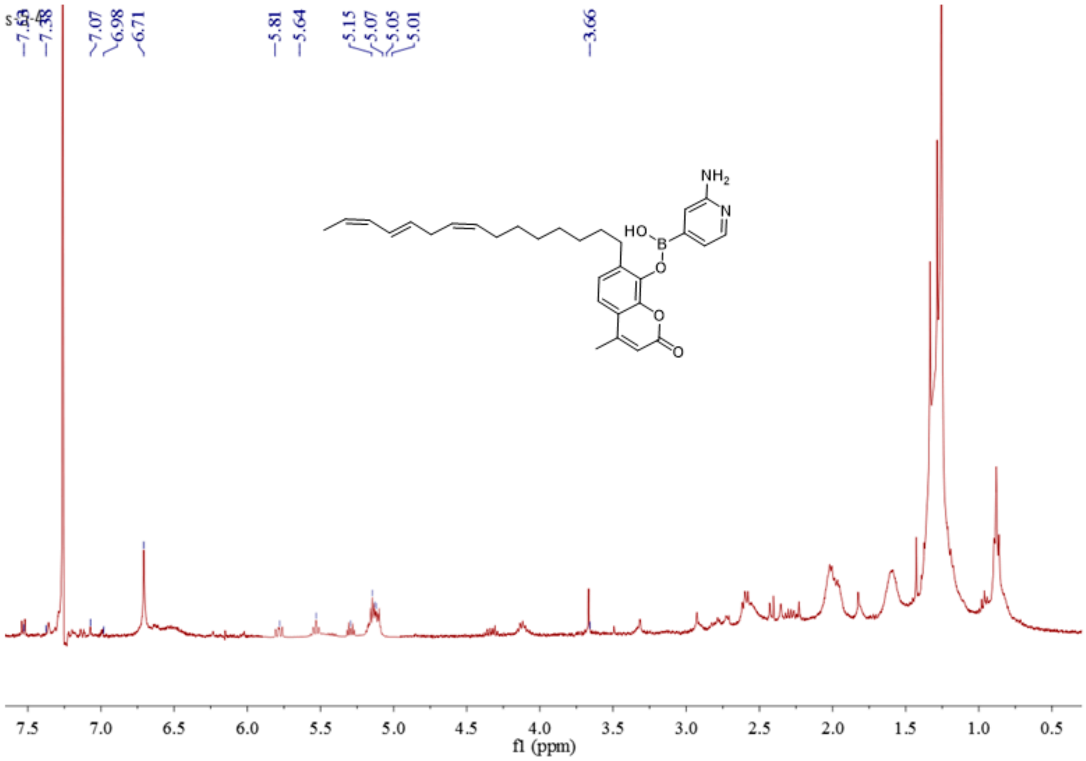

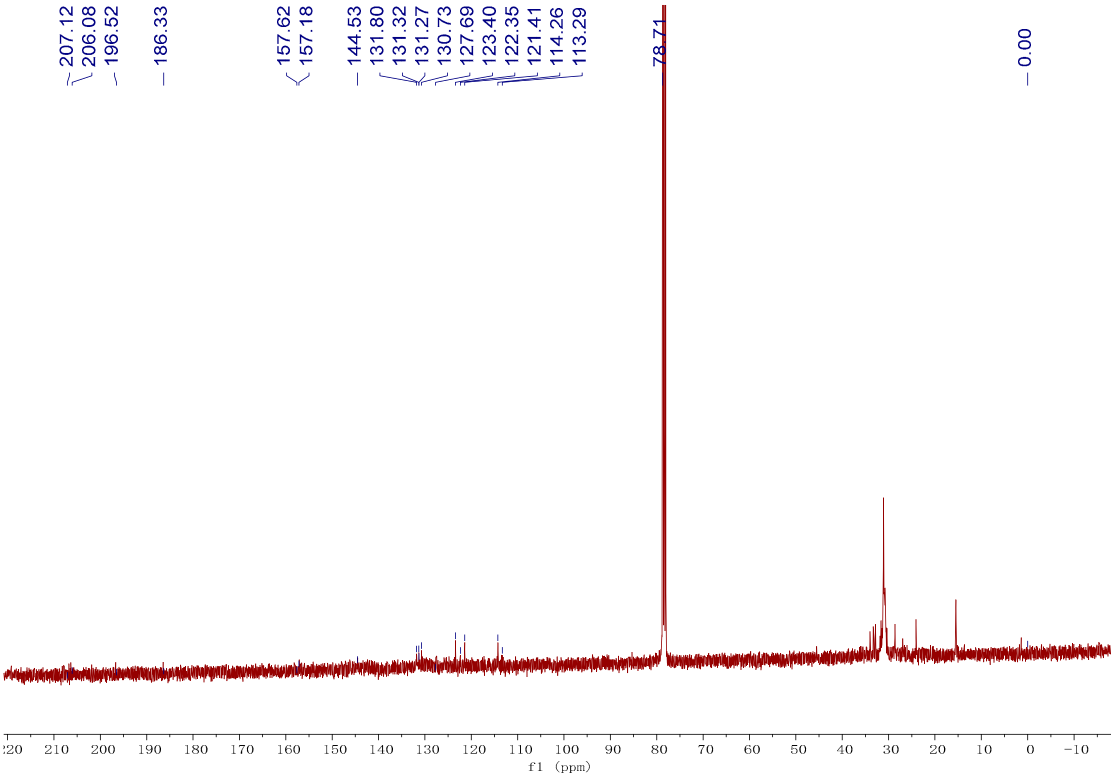

^{1}H/^{13}C NMR Compound 13: diene C15 urushiol NMR

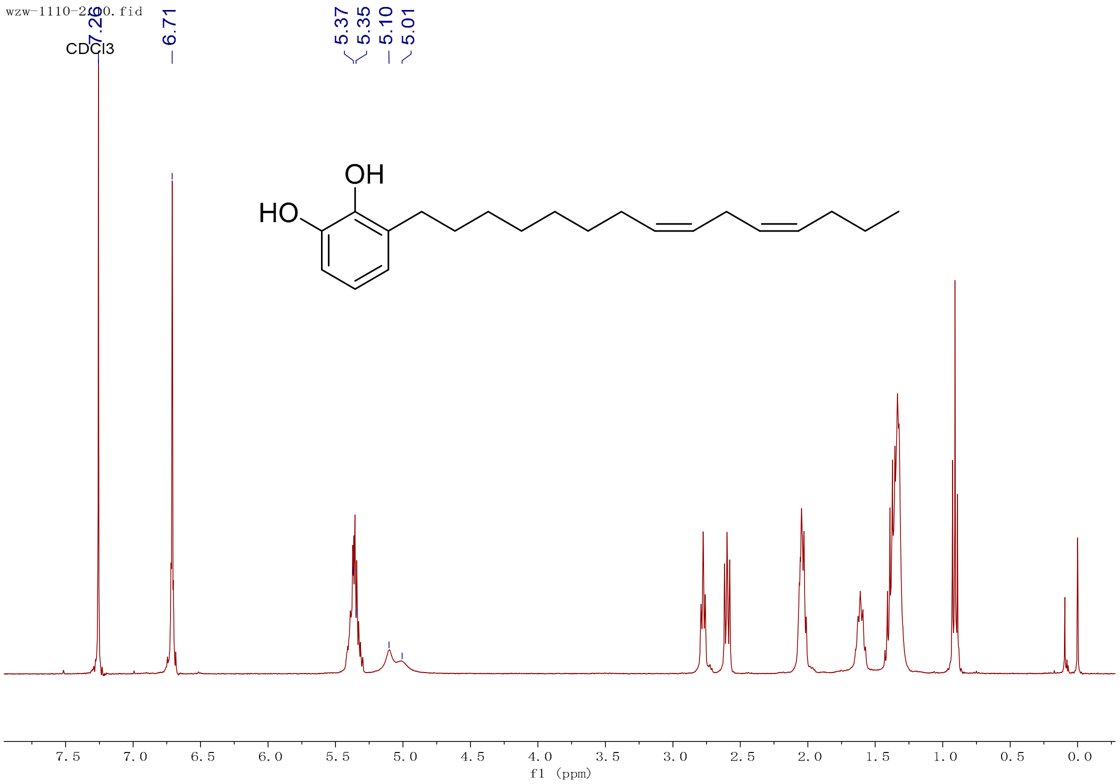

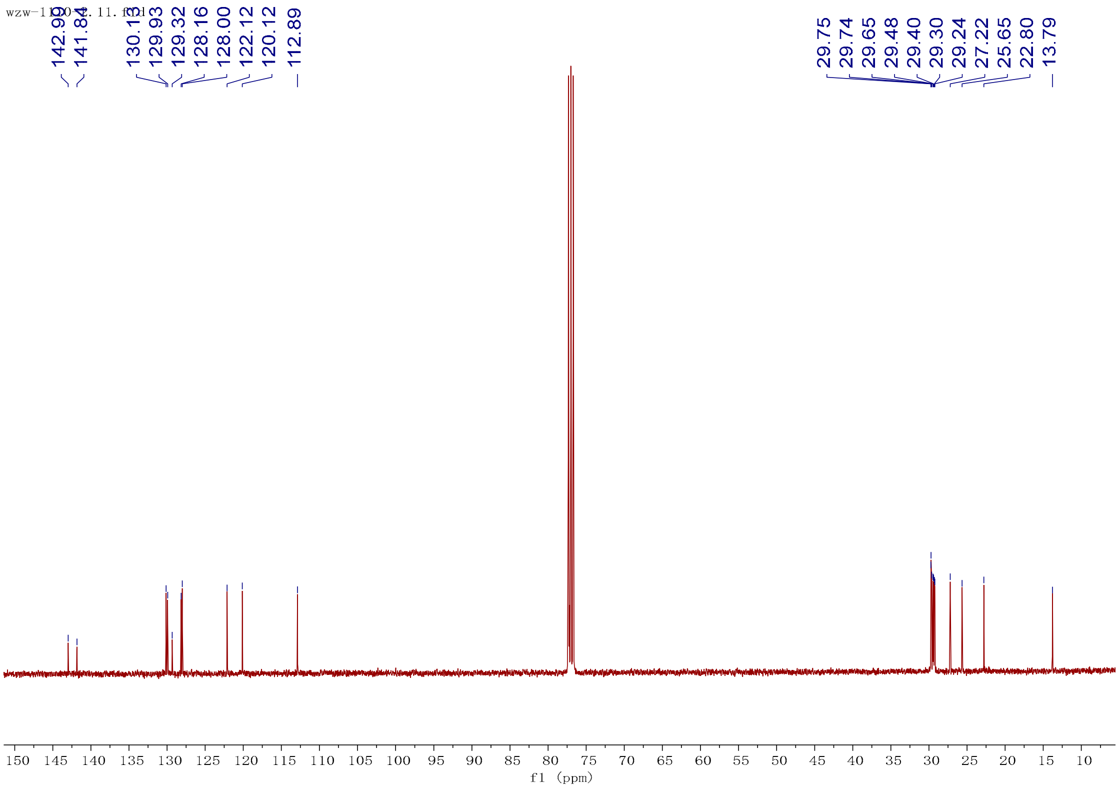

$^1H/^{13}C$ NMR Compound 14: triene C15 urushiol NMR

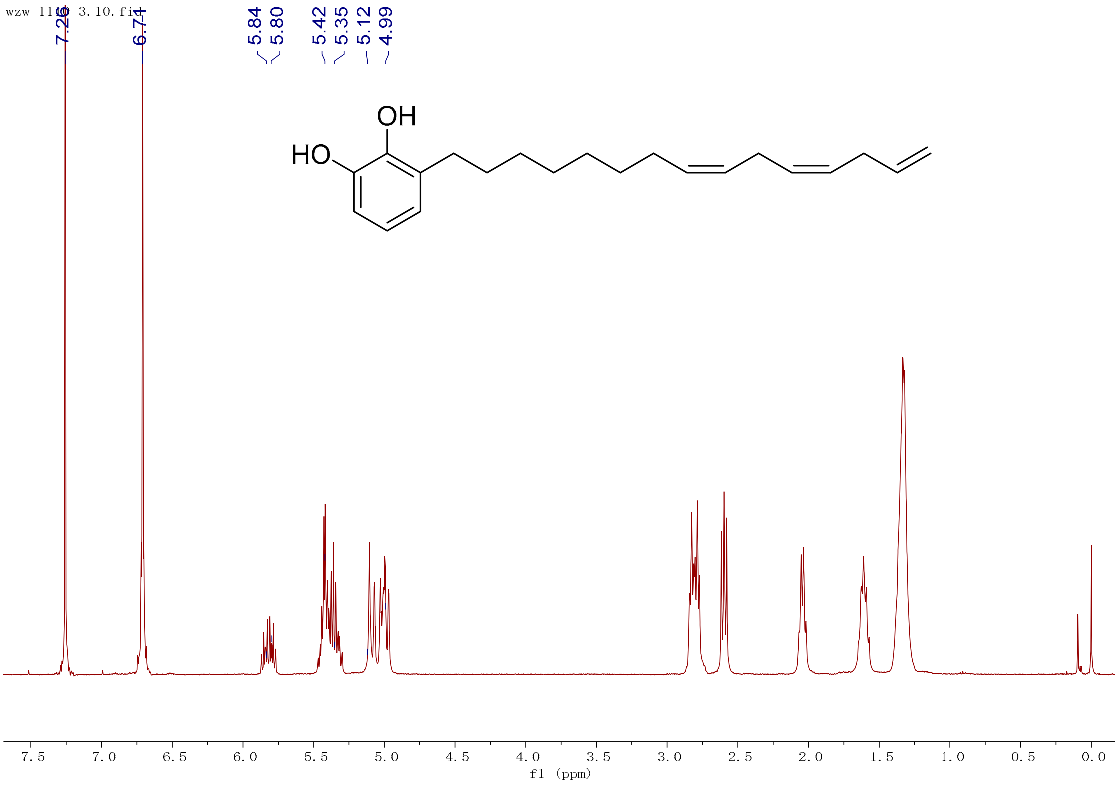

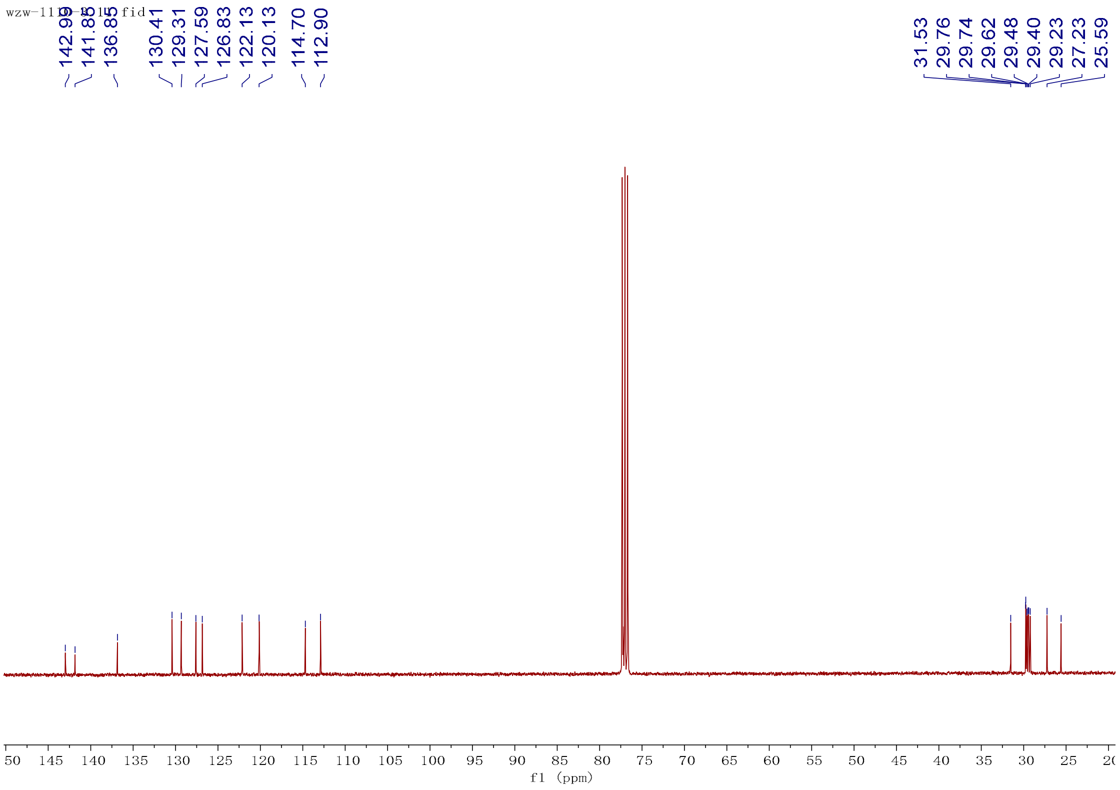

Compound 15 NMR

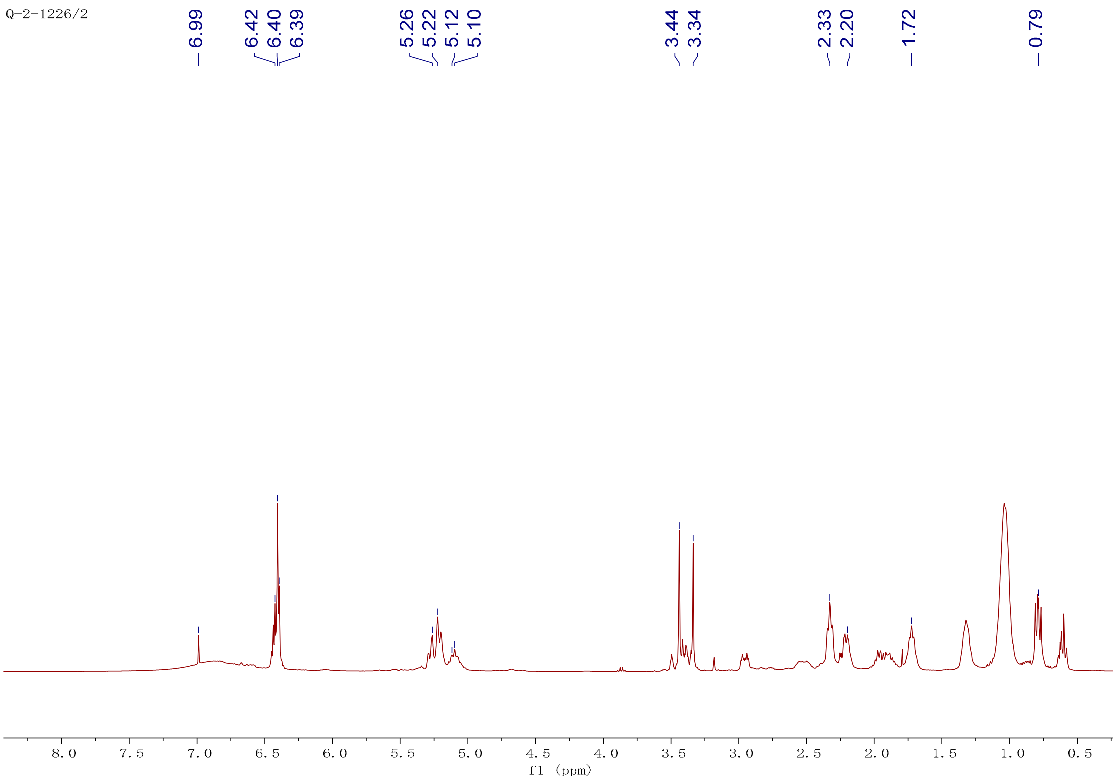

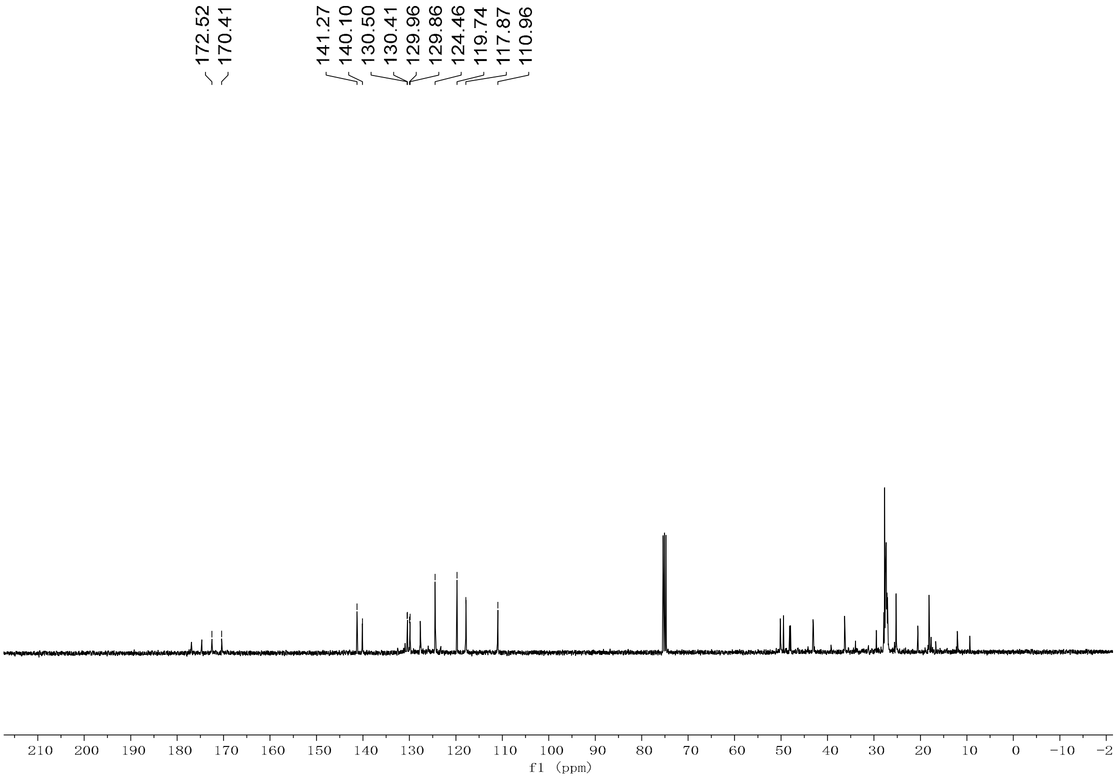

Compound 16 NMR

1a NMR 1H NMR (600 MHz, CDCl3) δ 6.72(m, 2H), 6.34(m, 1H), 5.99(m, 1H), 5.68(m, 1H), 5.37(m, 2H), 2.62(m, 2H), ESI-MS 503.2 [M+Na+]

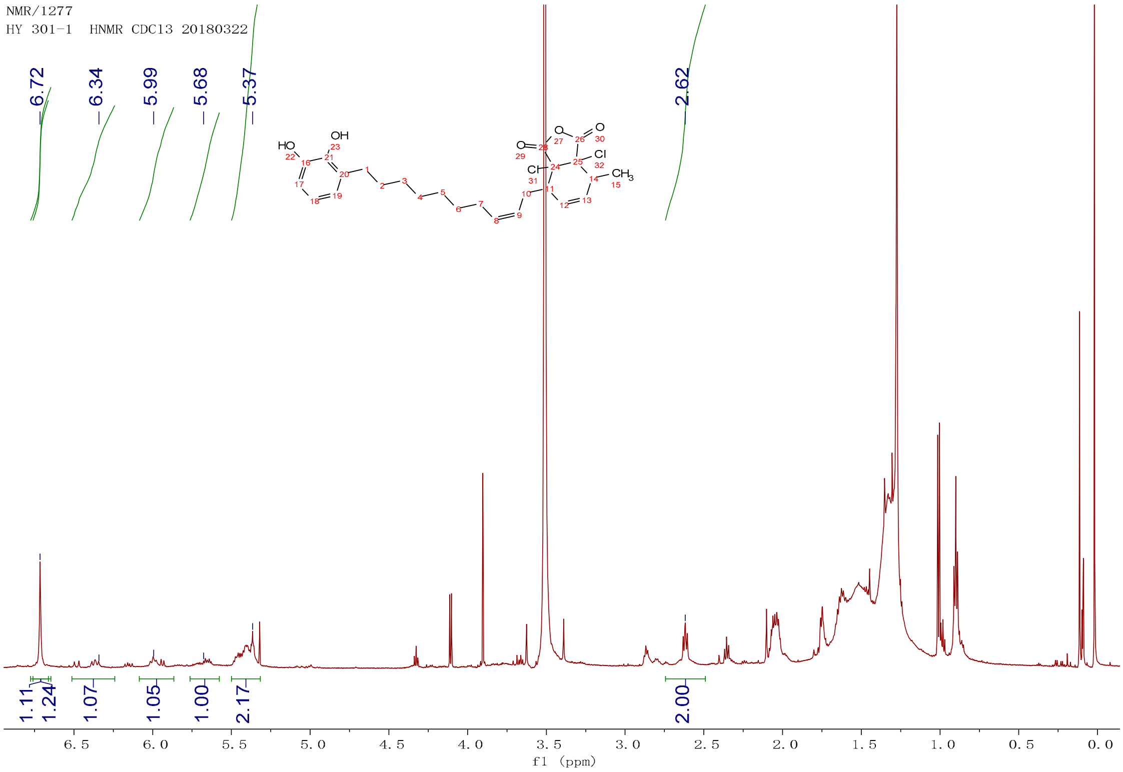

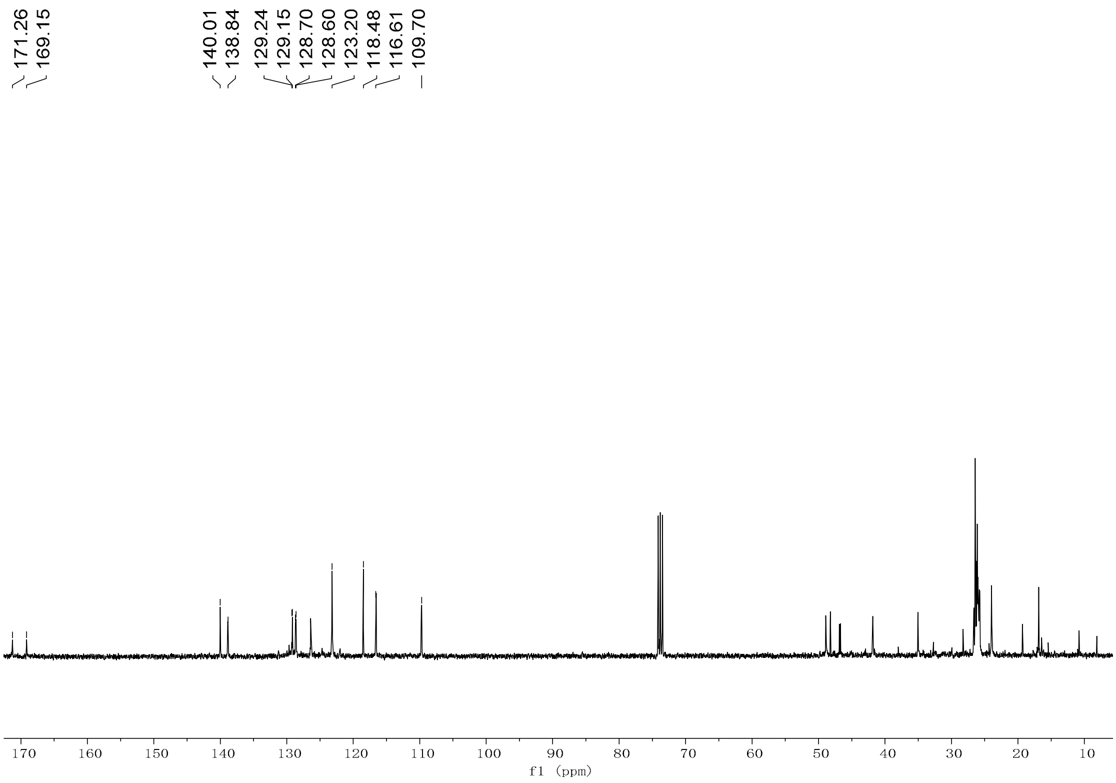

Compound 17 NMR

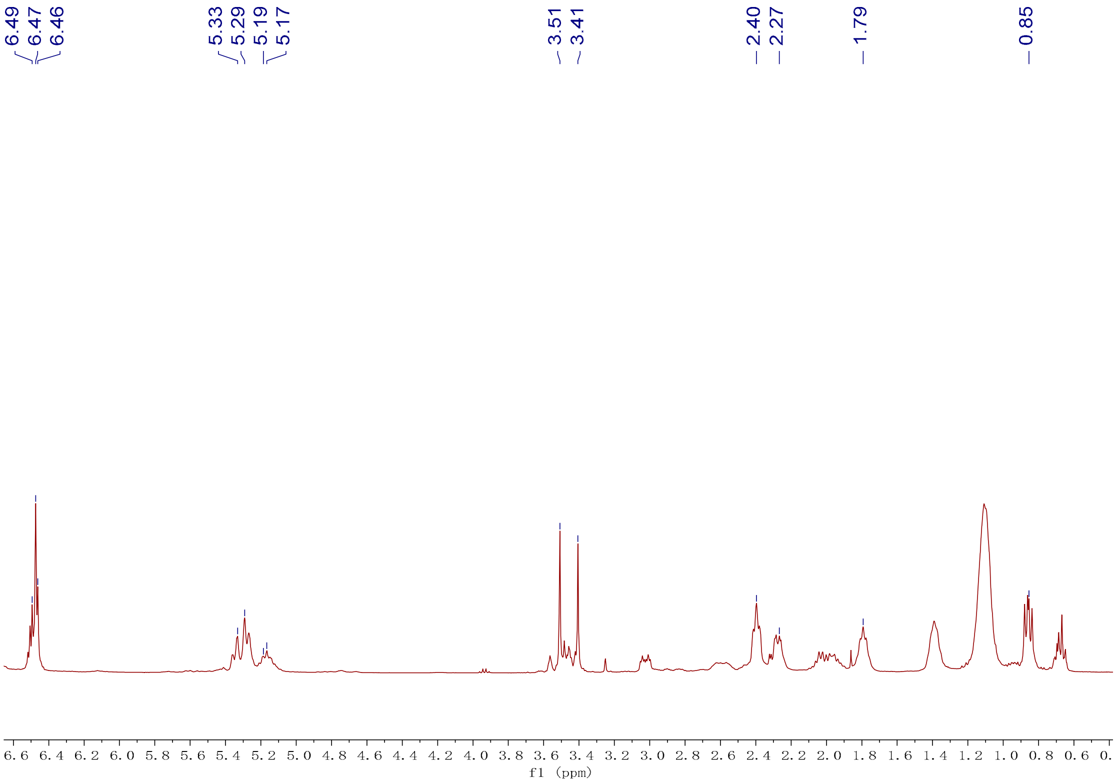

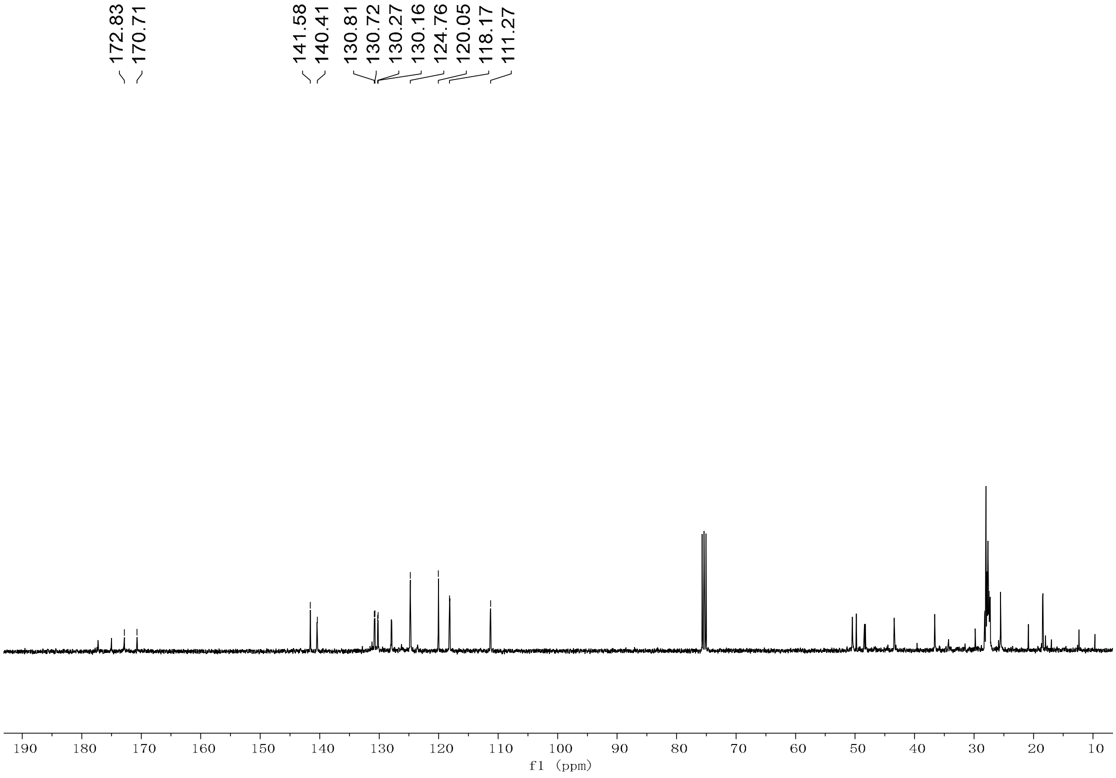

Compound 18 NMR

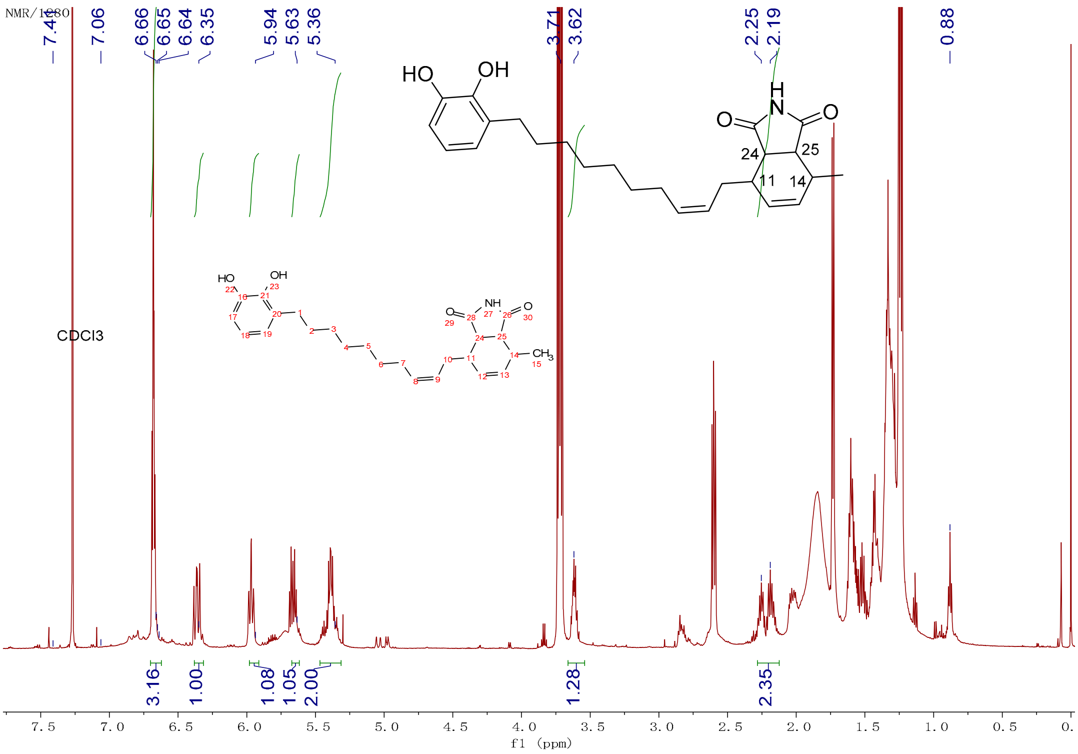

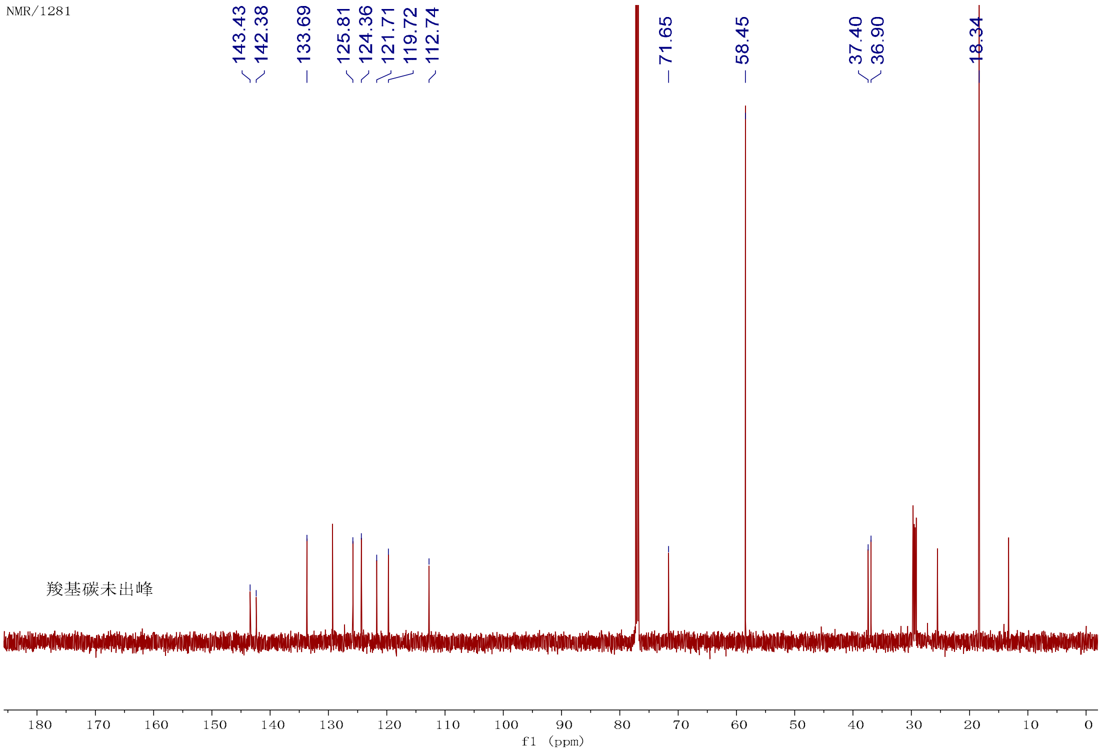

Compound 19 NMR

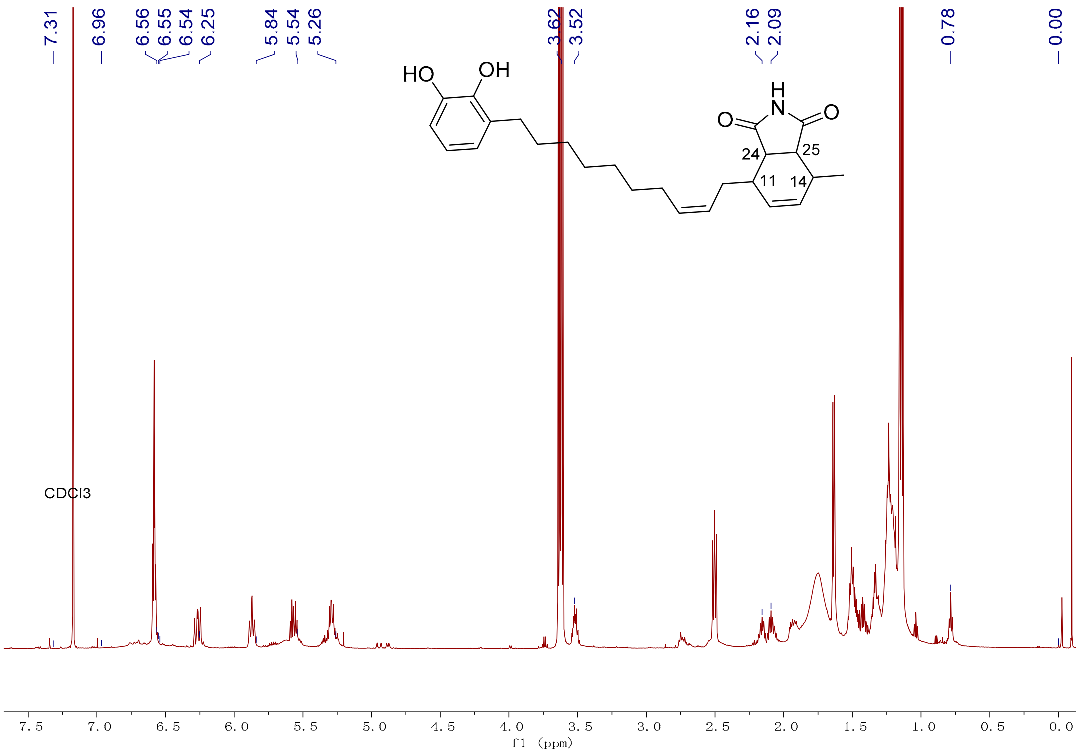

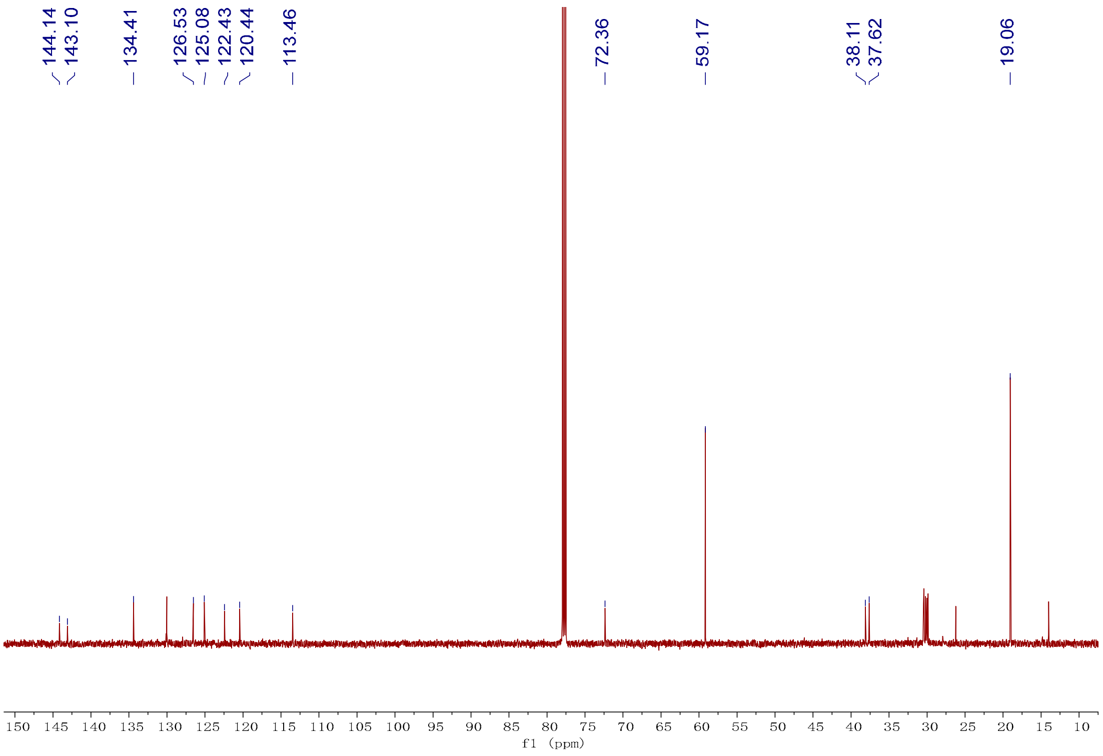

Compound 20 NMR

^{1}H NMR (600 MHz, $CDCl_3$) δ 9.12(-OH), 6.67 (d, *J* = 6.3 Hz, 2H), 6.33(m, 1H), 5.96(m, 1H), 5.64(m, 1H), 5.38(m, 2H), 2.59(m, 2H).

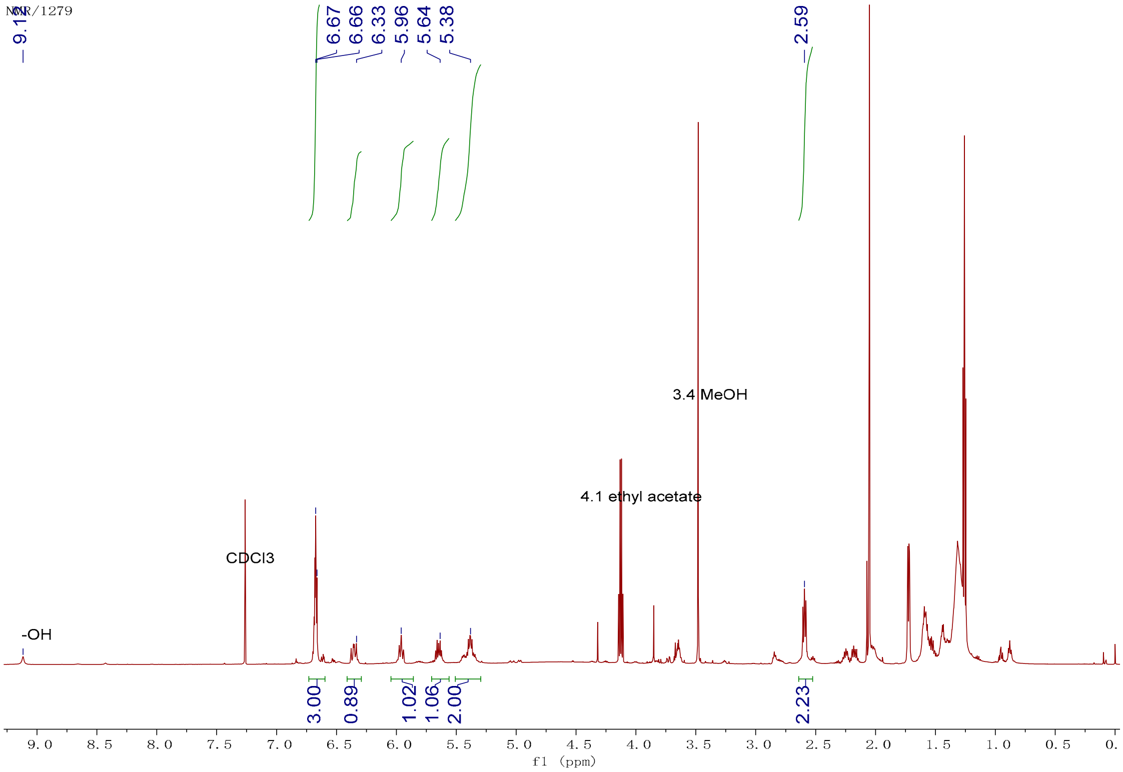

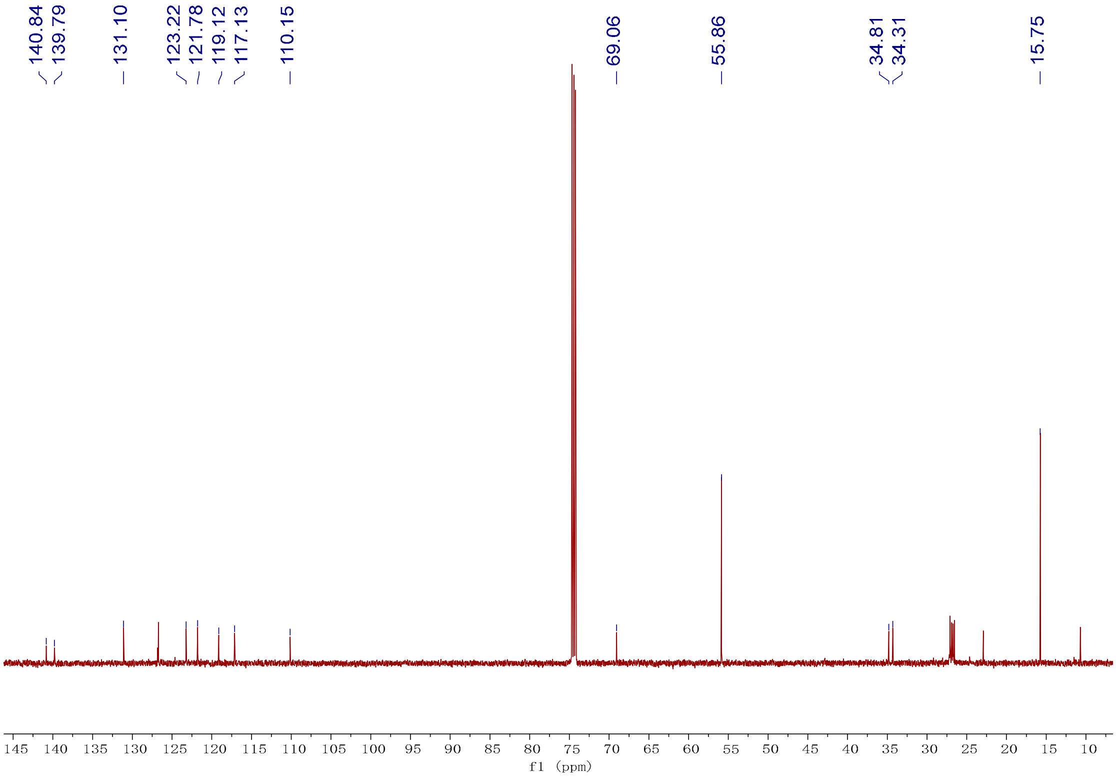

^{1}H/^{13}C NMR Compound 21

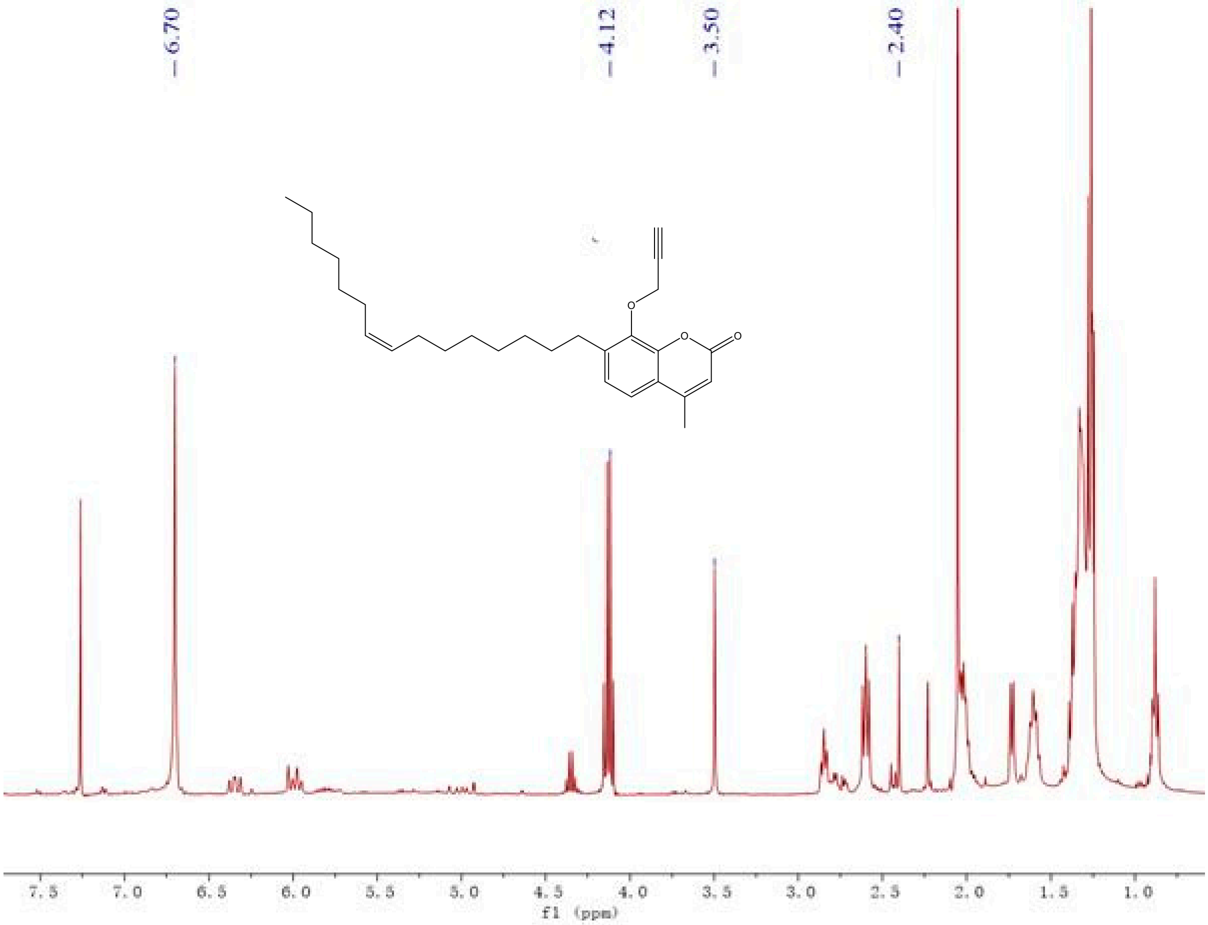

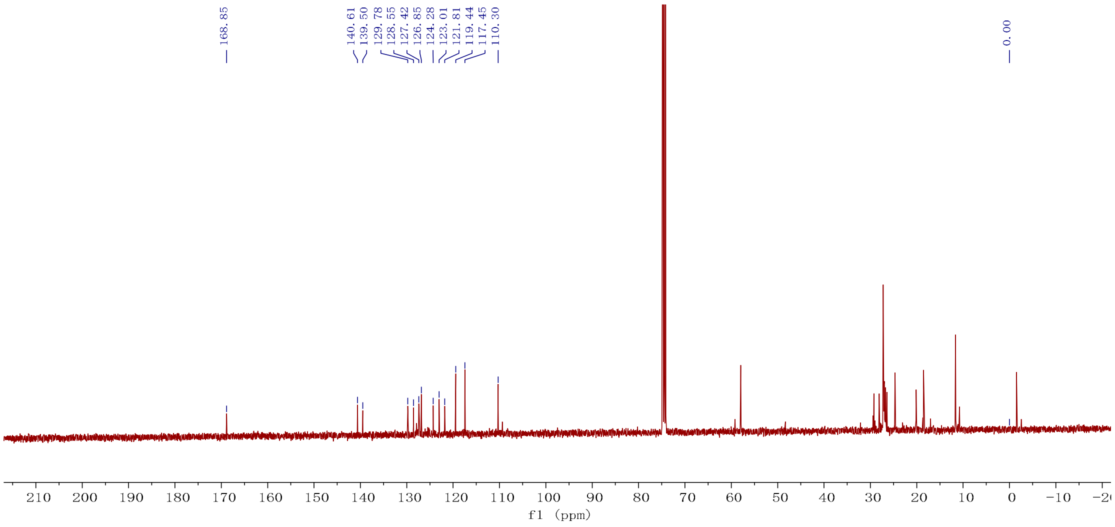

^{1}H/^{13}C NMR Compound 22

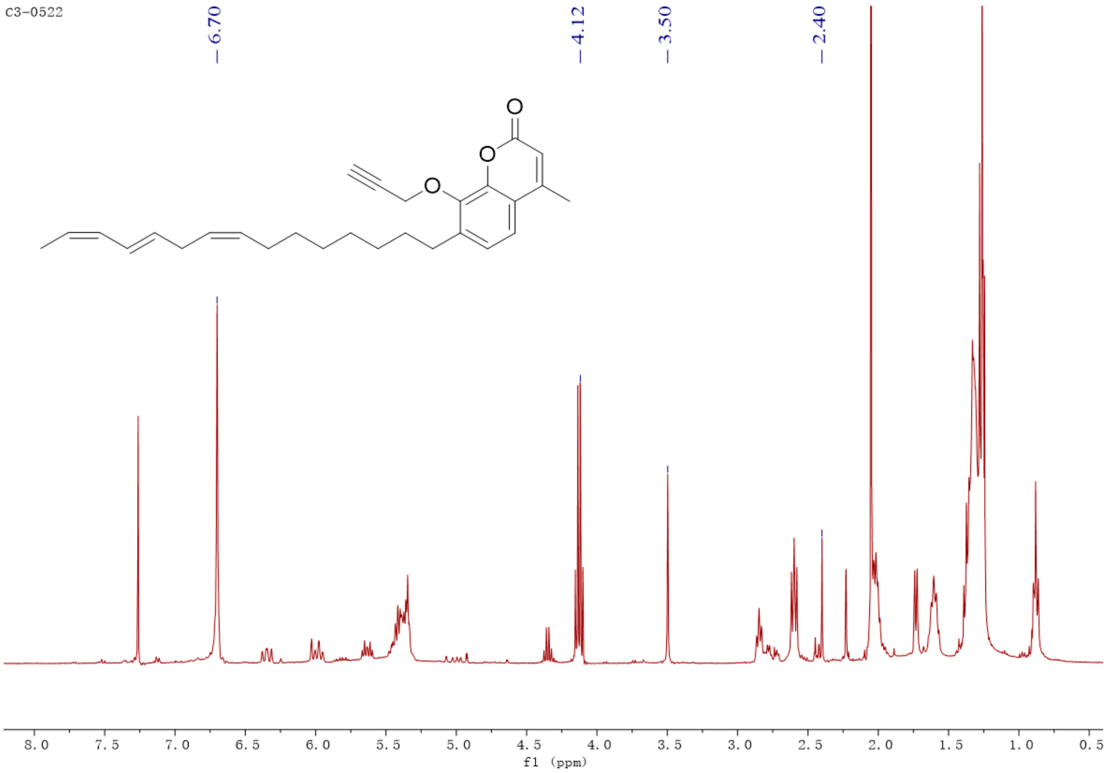

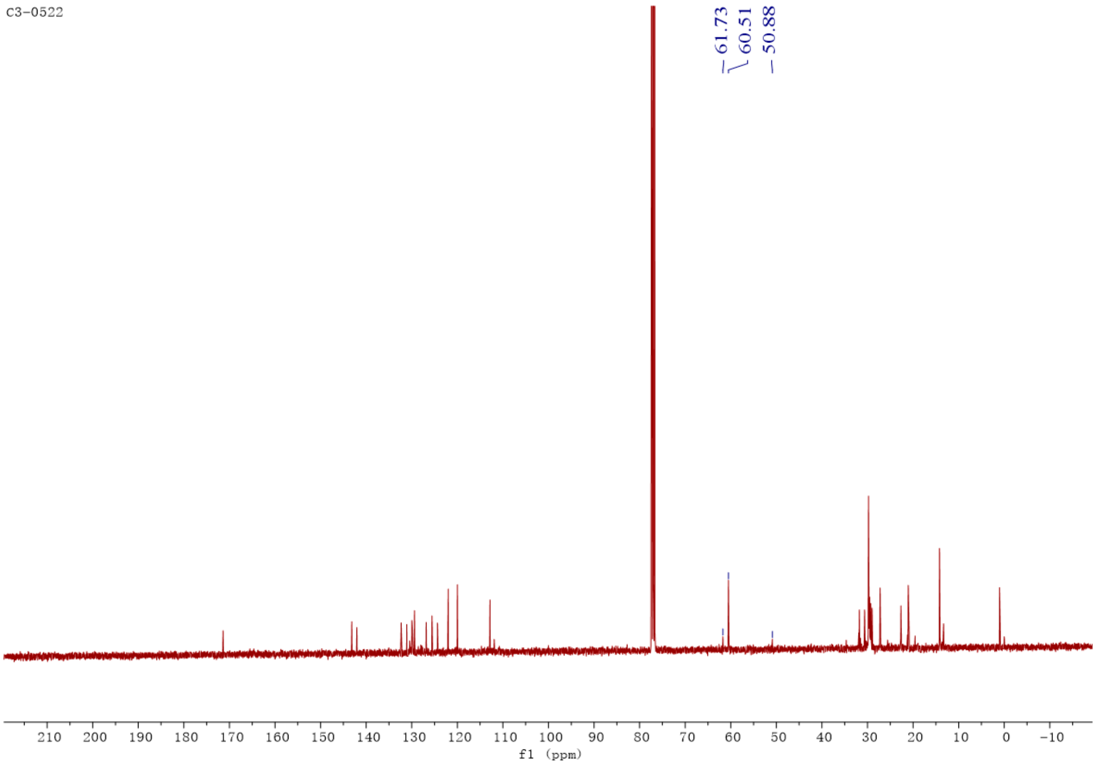

MS Compound 1

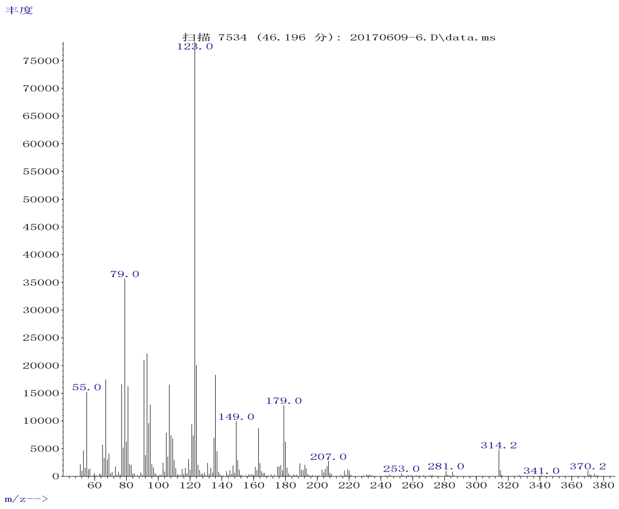

MS Compound 2

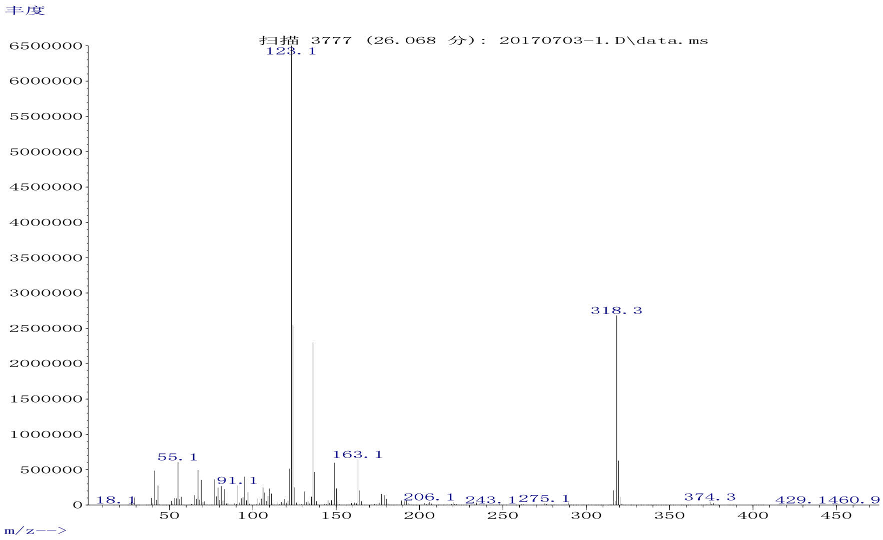

MS Compound 3

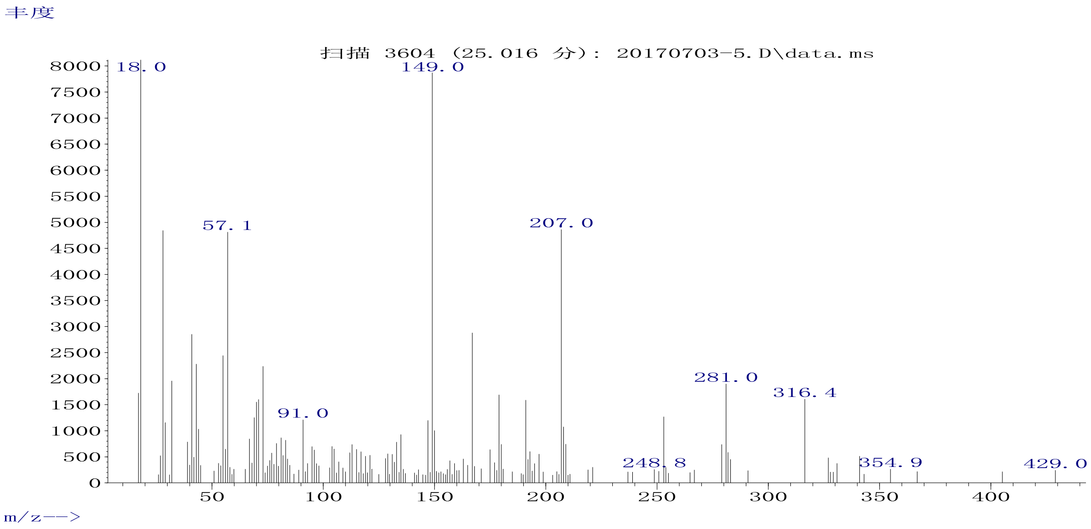

MS Compound 4

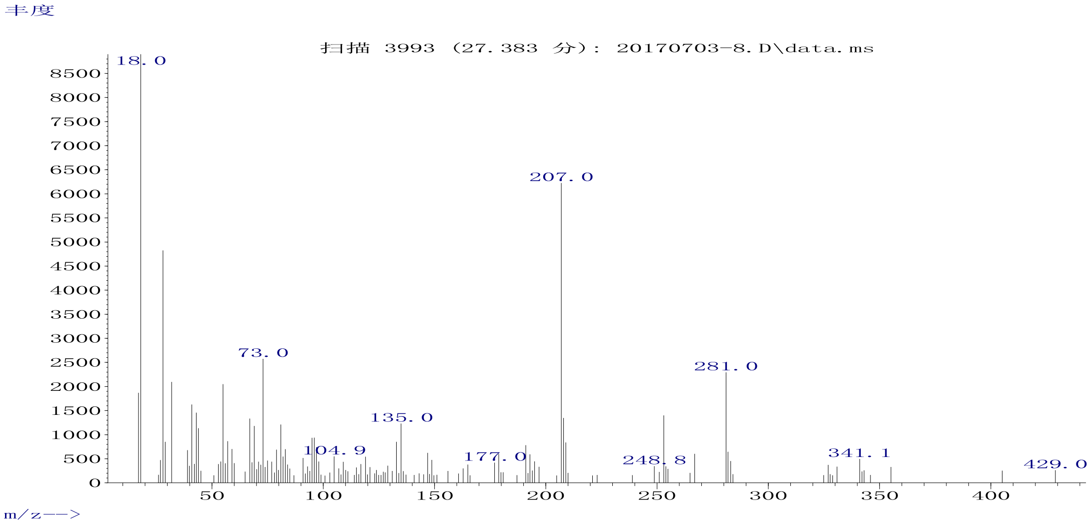

MS Compound 5 Levopimaric Acid

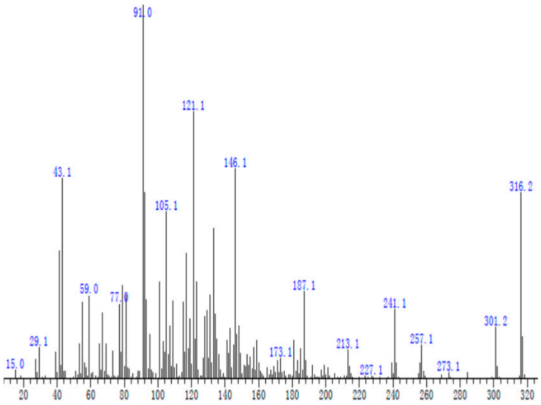

MS Compound 6

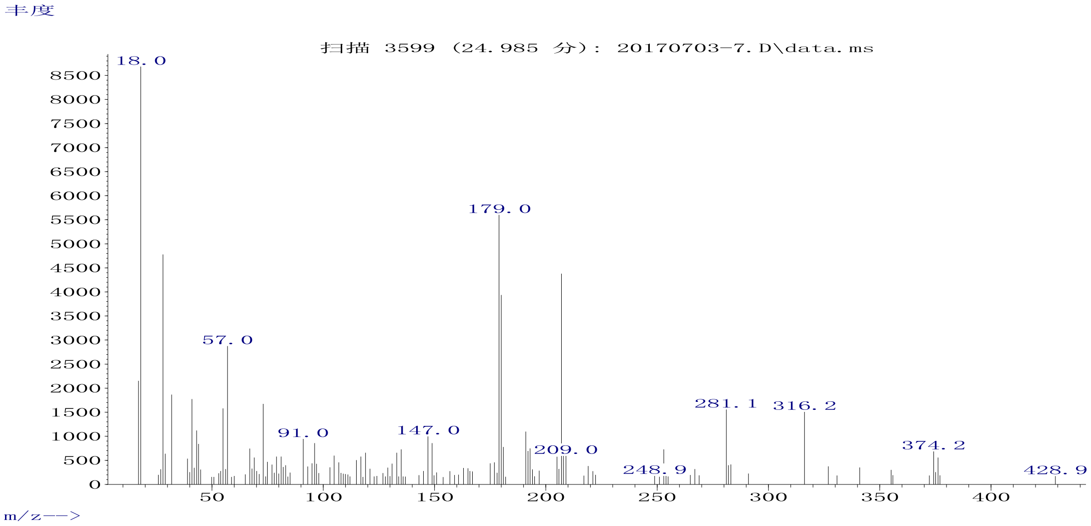

MS Compound 7

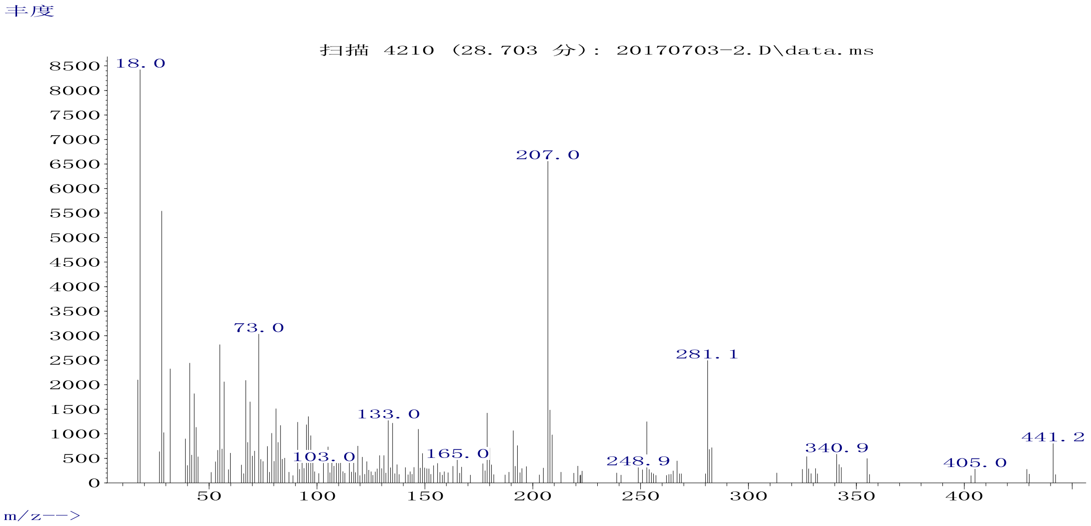

MS Compound 8

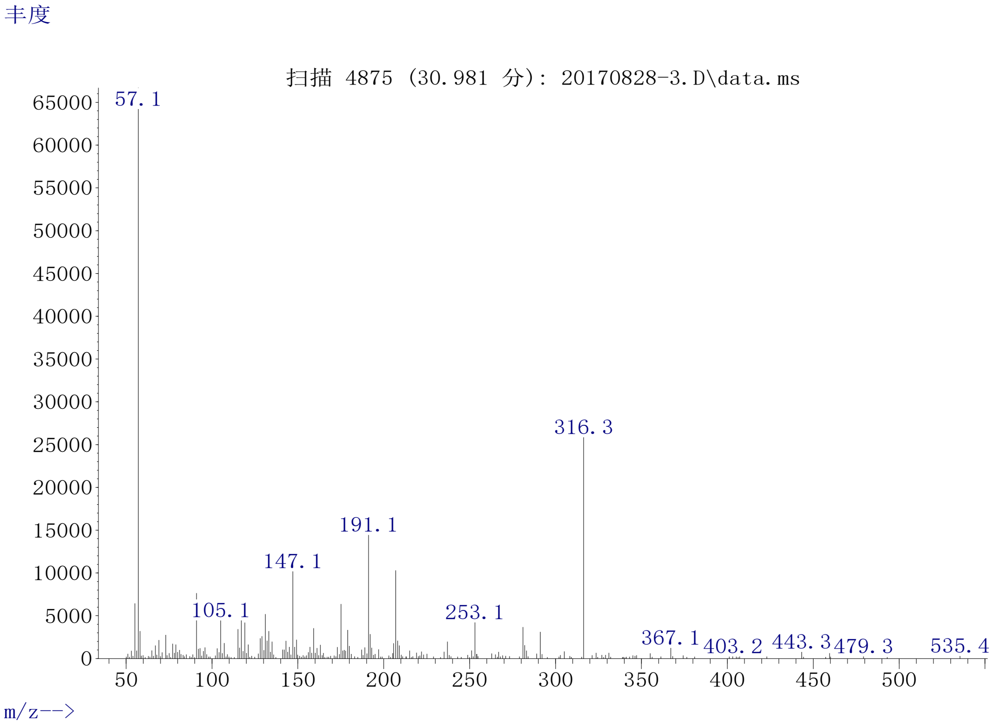

MS Compound 9

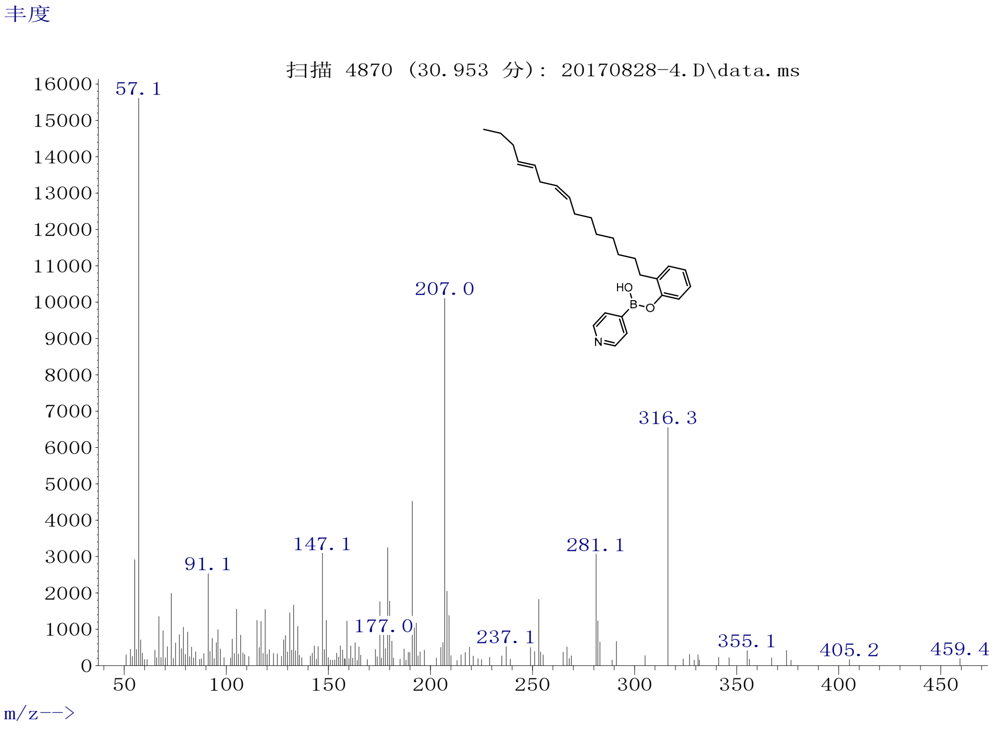

MS Compound 10

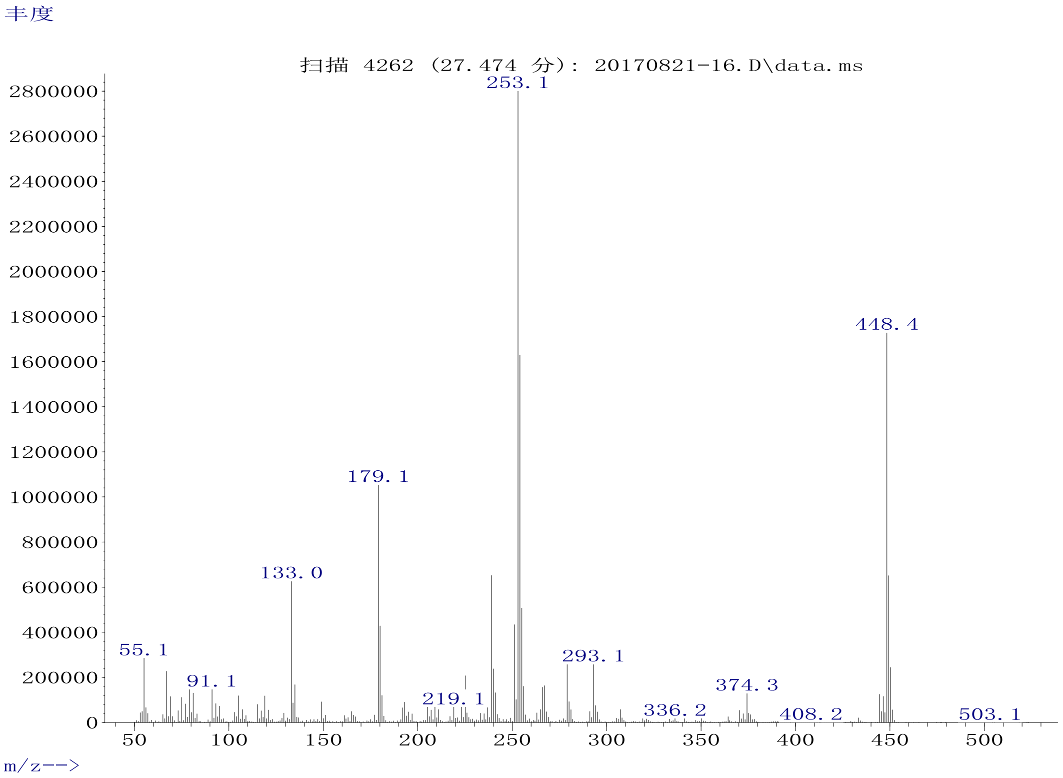

MS Compound 11

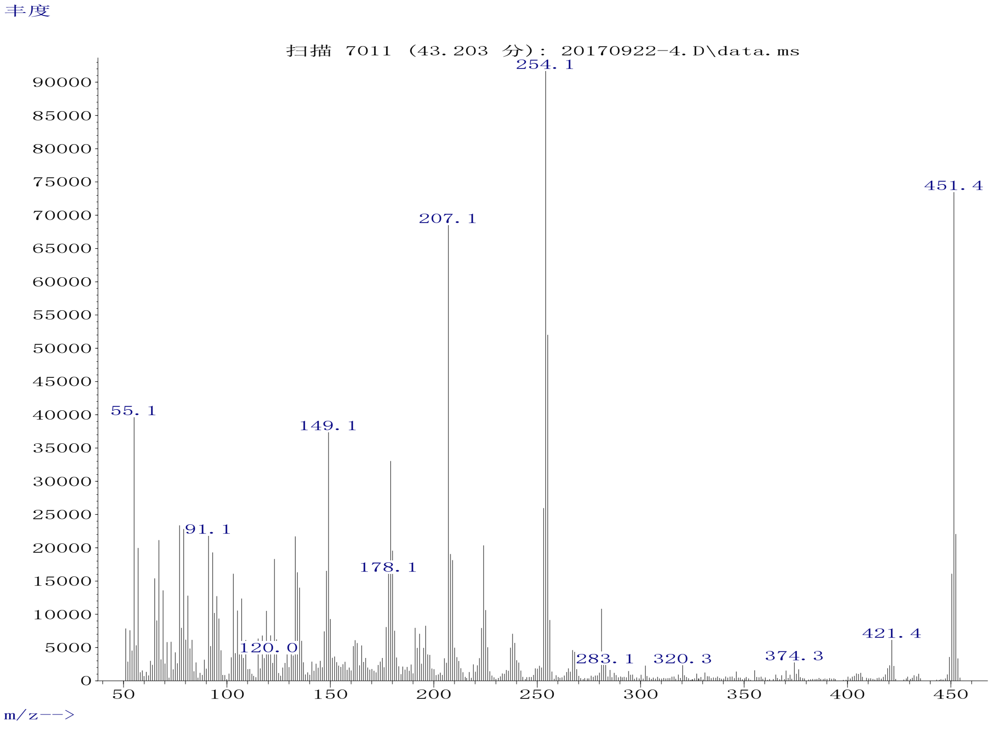

MS Compound 15, 16

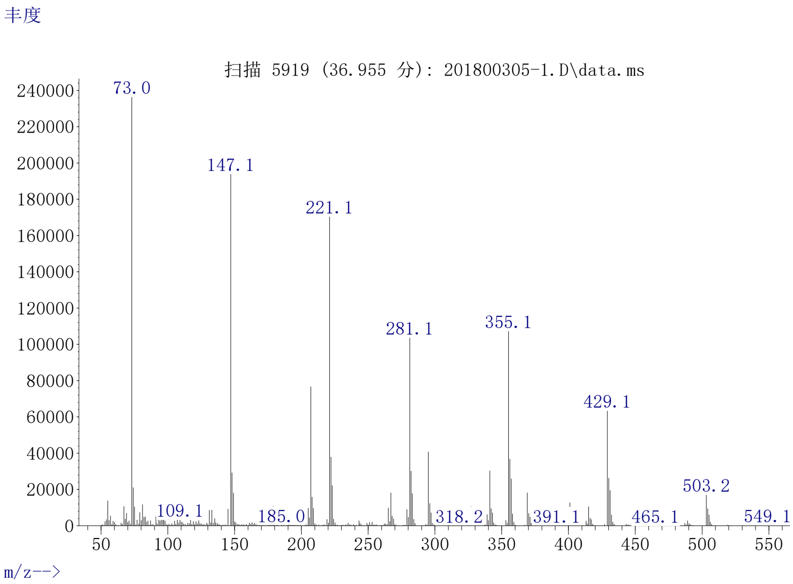

MS Compound 17

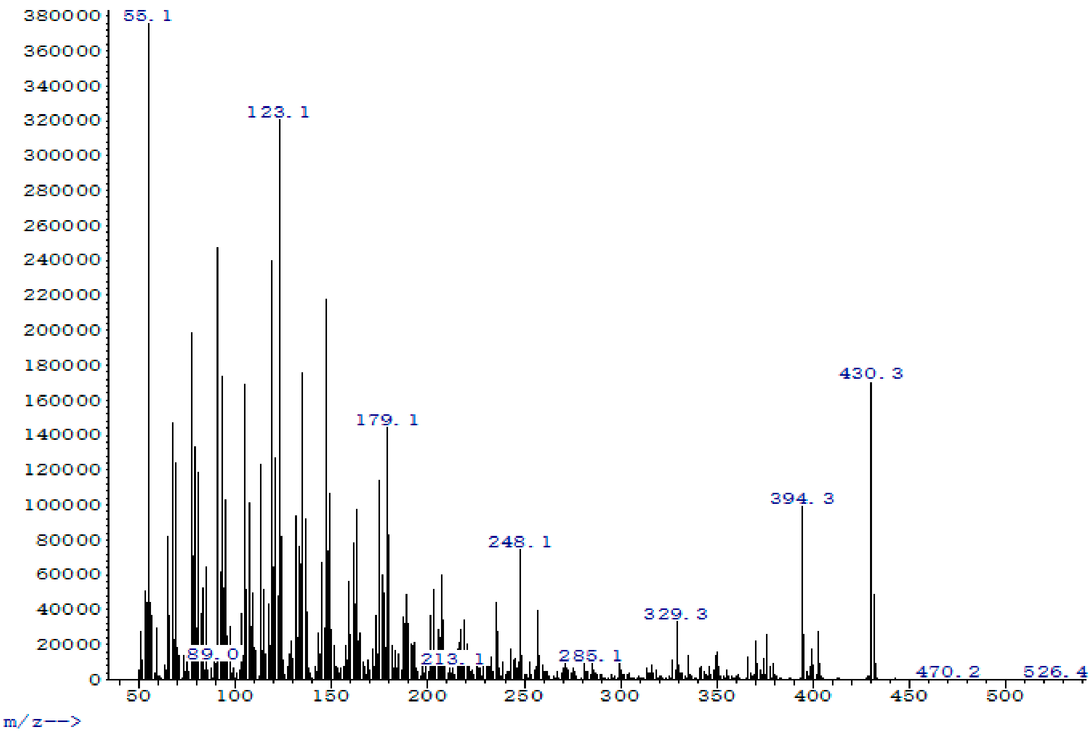

MS Compound 18

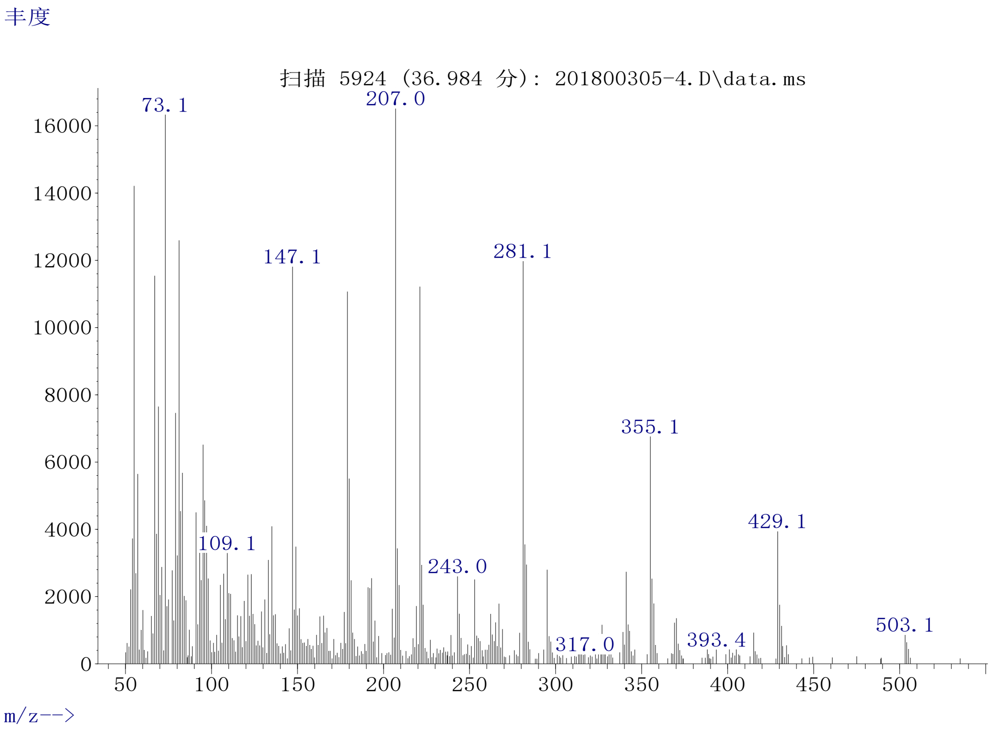

MS Compound 19, 20

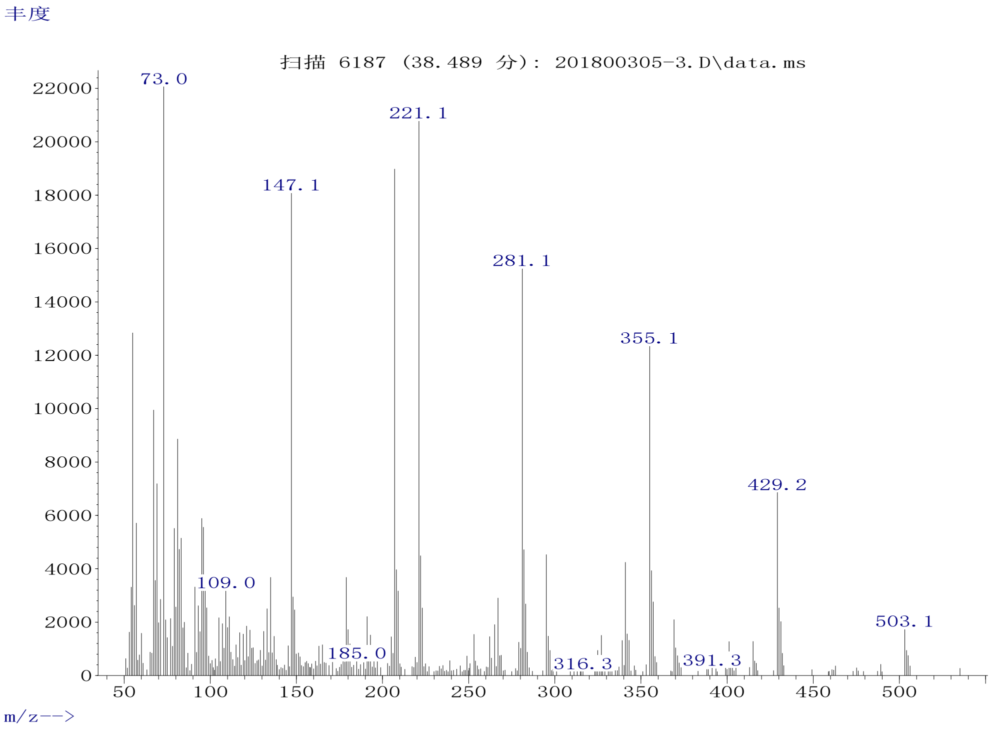

MS Compound 21

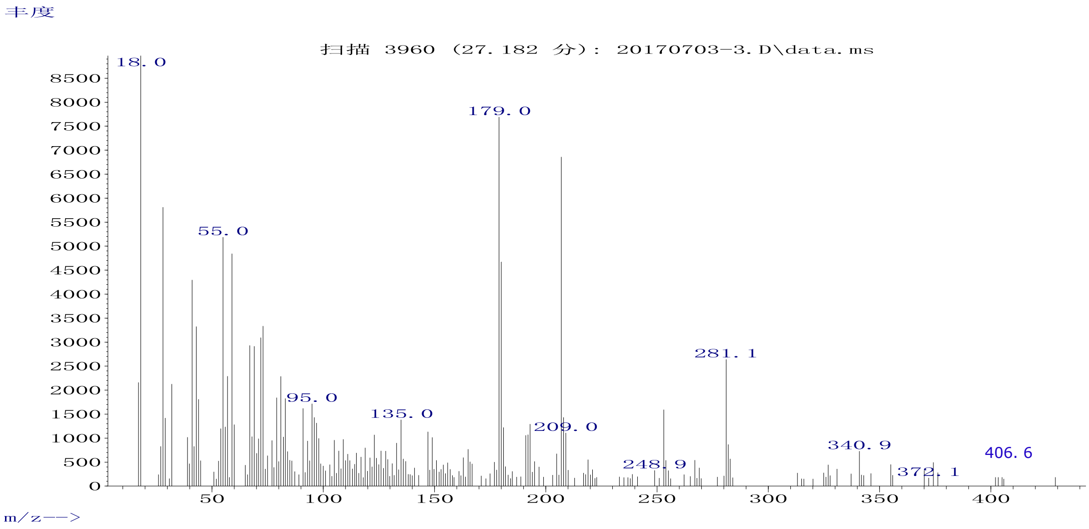

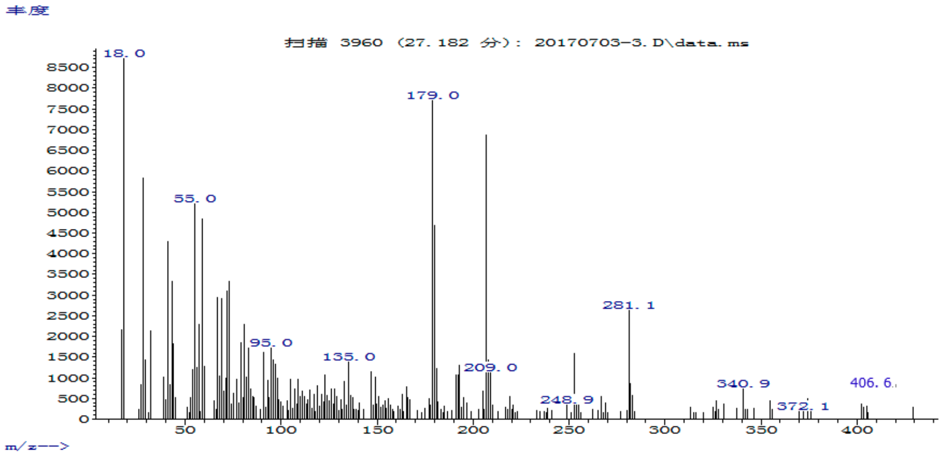

MS Compound 22

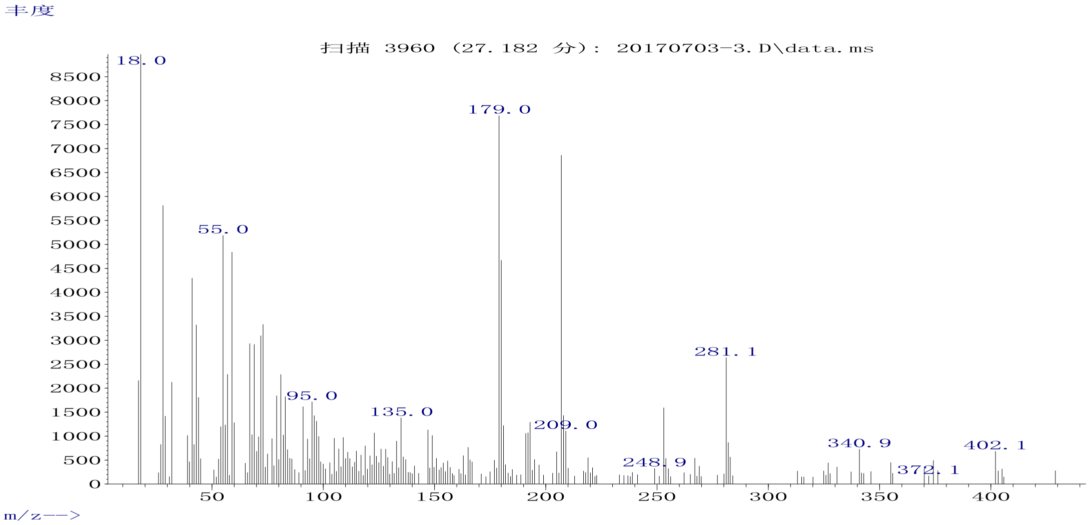

Supplement: Supplementary file 1 [file ijms-19-03184-s001.pdf]
